# The Peculiar Landscape of Repetitive Sequences in the Olive (*Olea europaea* L.) Genome

**DOI:** 10.1093/gbe/evu058

**Published:** 2014-03-26

**Authors:** Elena Barghini, Lucia Natali, Rosa Maria Cossu, Tommaso Giordani, Massimo Pindo, Federica Cattonaro, Simone Scalabrin, Riccardo Velasco, Michele Morgante, Andrea Cavallini

**Affiliations:** ^1^Department of Agricultural, Food, and Environmental Sciences, University of Pisa, Italy; ^2^Institute of Life Sciences, Scuola Superiore Sant'Anna, Pisa, Italy; ^3^Research and Innovation Center, Fondazione E. Mach di San Michele all’Adige, Italy; ^4^Institute of Applied Genomics, Udine, Italy; ^5^Department of Crop and Environmental Sciences, University of Udine, Italy

**Keywords:** genome landscape, *Olea europaea*, repetitive DNA, tandem repeats, retrotransposons, assembly of NGS reads

## Abstract

Analyzing genome structure in different species allows to gain an insight into the evolution of plant genome size. Olive (*Olea europaea* L.) has a medium-sized haploid genome of 1.4 Gb, whose structure is largely uncharacterized, despite the growing importance of this tree as oil crop. Next-generation sequencing technologies and different computational procedures have been used to study the composition of the olive genome and its repetitive fraction. A total of 2.03 and 2.3 genome equivalents of Illumina and 454 reads from genomic DNA, respectively, were assembled following different procedures, which produced more than 200,000 differently redundant contigs, with mean length higher than 1,000 nt. Mapping Illumina reads onto the assembled sequences was used to estimate their redundancy. The genome data set was subdivided into highly and medium redundant and nonredundant contigs. By combining identification and mapping of repeated sequences, it was established that tandem repeats represent a very large portion of the olive genome (∼31% of the whole genome), consisting of six main families of different length, two of which were first discovered in these experiments. The other large redundant class in the olive genome is represented by transposable elements (especially long terminal repeat-retrotransposons). On the whole, the results of our analyses show the peculiar landscape of the olive genome, related to the massive amplification of tandem repeats, more than that reported for any other sequenced plant genome.

## Introduction

Large genomes are filled with repetitive sequences, especially in plants (Morgante et al. 2007). Although some repeats appear to be nonfunctional, others could have played a role in the evolution of a species (see e.g., Britten 2010), acting as independent, “selfish” sequence elements (Hua-Van et al. 2011), or creating novel functions (Morgante et al. 2005), or modelling the regulatory patterns of genes that result in phenotypic variation (Knight 2004).

Repeats arise from a variety of biological mechanisms that result in extra copies of a sequence being produced and inserted into the genome. They can be widely interspersed repeats, tandem repeats, or nested repeats, and occur even in millions of copies, ranging in size from one to two bases to thousands of bases. In some cases, only a few repeat families account for the majority of genomic DNA (in the human genome, e.g., the family of Alu repeat elements cover ∼11% of the genome, Rowold and Herrara 2000); in other large genomes, no prominent repeat families are found, but many low redundant repeat classes account for the majority of genomic DNA (Cavallini et al. 2010). Generally, the most redundant sequences in plants are transposable elements, especially retrotransposons (REs) belonging to *Gypsy* and *Copia* superfamilies, which transpose via a copy-and-paste mechanism (Wicker et al. 2007). For example, they cover ∼80% of the maize genome (Schnable et al. 2009). In certain cases, as in the short-lived fish *Nothobranchius furzeri,* another class of sequences, the tandem repeats are prominent (21%) in the genome (Reichwald et al. 2009).

Tandem repeats are commonly known as satellite DNAs (Schmidt and Heslop-Harrison, 1998), because they were initially isolated from satellite bands in experiments with gradient centrifugation, due to the difference in A+T content from the rest of genomic DNA (Szybalski, 1968). Such sequences are arranged in tandem repeating units, where individual copies lie adjacent to each other. They are found preferentially at specific positions of the chromosomes, such as the pericentromeric, subtelomeric, telomeric, or intercalary regions (Kubis et al. 1998). Tandem repeats from *Secale cereale* were among the first satellite DNAs isolated, representing more than 6% of the rye genome (Bedbrook et al. 1980).

Families of tandem repeats show different homology, redundancy, and distribution pattern between related species of a plant genus or family, exhibiting species-, genome-, and even chromosome specificity (Wang et al. 1995). For example, within the plant genus *Cucurbita*, one satellite was detected differing in copy number among species, and another was present in a similar number of copies (King et al. 1995). By contrast, dramatic variation in copy number is reported for all satellites in all species within the tribe Triticeae (Vershinin and Heslop-Harrison 1998). In three species of the genus *Chironomus*, beside copy number variation, chromosomal localization of the same tandem repeat is also detected (Ross et al. 1997). Within a species, a satellite DNA shows sequence variability that depends on the ratio between the mutation and homogenization rates (Dover 1986). In this sense, each satellite DNA can be regarded as an independent evolutionary unit (Ugarkovic and Plohl 2002).

Many basic questions about the evolution of plant genomes remain unanswered, especially regarding the occurrence of similar patterns of evolution among species. Genome evolution is based on the equilibrium between genome size increase by polyploidy and retrotransposon amplification and decrease by retrotransposon-mediated DNA loss (Morgante et al. 2007; Proost et al. 2011). The role of satellite DNAs in this respect is still largely unknown. Next-generation sequencing (NGS) procedures can also be conveniently used to study such dynamics by performing a global survey of the genome in species whose genome has not been sequenced yet (Swaminathan et al. 2007; Treangen and Salzberg 2012).

The olive genome is largely uncharacterized, despite the growing importance of this tree as oil crop. Olive (*Olea europaea* L.) has a medium-sized haploid genome of 1.4–1.5 Gb (Loureiro et al. 2007). Concerning repeated sequences, the best characterized are four tandem repeats isolated from genomic libraries and, in some instances, localized by cytological hybridization on olive chromosomes (Katsiotis et al. 1998; Bitonti et al. 1999; Minelli et al. 2000; Lorite et al. 2001; Contento et al. 2002). Also putative retrotransposon fragments have been isolated and sequenced (Stergiou et al. 2002; Natali et al. 2007), but a comprehensive picture of repeat elements landscape in the olive genome is still lacking.

In the frame of a project aiming to obtain the complete sequence of the olive genome, we performed a deep analysis of the repetitive component of this genome, using NGS techniques (454-Roche and Illumina). In this work, we used different computational procedures to isolate and characterize olive-repeated sequences. These data were used to determine the structure of the genome and the composition of its repetitive fraction. The results indicated that olive genome structure is peculiar among plant genomes, with a very large percentage of satellite DNA, related to a few tandem repeat families.

## Materials and Methods

### Illumina and 454 Sequencing

Genomic DNA was extracted starting from young leaves of *O. europaea* cv “Leccino” following the nuclei extraction protocol of Zhang et al. (1995), modified for small volumes.

Paired-end libraries were prepared as recommended by Illumina (Illumina Inc., San Diego, CA) with minor modifications. Illumina reads were preprocessed to remove Illumina adapters using Cutadapt (Martin 2011) with default parameters but -O 10 -n 2 -m 50. An internally developed Perl script was used to remove unpaired reads. In order to trim low-quality regions, reads were further processed with ERNE-FILTER (erne.sourceforge.net, last accessed April 1, 2014) using default parameters but –min-size 50.

For 454 sequencing, two random shotgun “genomic” libraries were generated via fragmentation of 500 ng each of genomic DNA employing the GS FLX+ Series XLR70 and XL+ Rapid Library preparation kit following the manufacturer’s recommendations (Roche, Indianapolis, IN).

Low-quality bases, empty reads, and adapter sequences were removed using CLC-BIO Genomic Workbench, version 5.1 (CLC-BIO) and ERNE-FILTER (Del Fabbro et al. 2013).

With Illumina technology, we obtained 157,049,970 paired-end reads, with mean read length, after trimming for base quality, of 98.6 nt. From these reads, we produced two sets of sequences. The first set included 28,875,848 paired-end reads, corresponding to 2,847,904,818 nt and 2.03 genome equivalents, and was used for assembly. The second set included 151,945,027 paired-end reads that were trimmed at 75 nt in length, corresponding to 11,395,877,025 nt and 8.1 genome equivalents, and was used for mapping-based estimation of sequence redundancy. With 454 technology, we obtained 8,079,610 single reads, with mean read length of 407 nt, corresponding to a total of 3,275,110,538 nt and 2.3 genome equivalents.

### Graph-Based Clustering of Sequences

The graph-based clustering method (Novák et al. 2010) was not feasible on the full data set because of computational requirements. Two reduced sets of randomly selected genomic Illumina and 454 reads (1× coverage for each set) were separately subjected to clustering using RepeatExplorer website (http://repeatexplorer.umbr.cas.cz/, last accessed April 1, 2014). The output of RepeatExplorer was also used to prepare two in-house libraries of olive repetitive sequences, the first containing tandem repeat sequences (characterized by sequence similarity search and structural analysis using DOTTER, Sonnhammer and Durbin 1995) and the second containing all contigs belonging to clusters identified by RepeatExplorer as retrotransposons, DNA transposons, rDNA, by similarity search against internal databases of known repeats (RepeatMasker, protein domains). These libraries were used for the annotation of assembled sequences (see next section).

### Sequence Assembly Procedure

Illumina (2.03 genome equivalents) and 454 (2.3 genome equivalents) reads were assembled using CLC-BIO Genomics Workbench, version 5.1 (CLC-BIO). Initially, we performed a simple assembly of Illumina reads and obtained 1,788,026 contigs that were further assembled using Minimus 2 assembler (Sommer et al. 2007) using an overlap length cutoff of 40 bp and an overlap identity cutoff of 90%. Alternatively, the pool of Illumina reads was split into 16, 64, 256, or 512 subpackages and assembled by CLC-BIO separately into contigs, based on unambiguous overlapping (indicated as split 0, 16, 64, 256, and 512, respectively); for each splitting, the resulting contigs were assembled on their turn using Minimus 2 assembler with an overlap length cutoff of 40 bp and an overlap identity cutoff of 80%.

Also 454 reads were assembled using CLC-BIO. Possible contaminants resembling organellar sequences were removed by all assemblies masking contigs against an in-house olive organellar sequence database using RepeatMasker (Jurka 2000; http://www.repeatmasker.org/, last accessed April 1, 2014). A conservative threshold of 1% similarity was used for excluding any contamination of organellar sequences in the nuclear data set.

Finally, all Illumina supercontigs and single contigs were assembled with 454 contigs to produce a whole genome set of assembled sequences (WGSAS) in which single contigs longer than 1,000 nt were also included.

### Estimation of Sequence Abundance

Redundancy of each supercontig or individual contig in the WGSAS was estimated by mapping a large Illumina sequence read set (total coverage 8.1×) onto the WGSAS. To obtain uniformly long reads, all bases exceeding 75 nt were cut. Mapping was performed using CLC-BIO, which distributes multireads randomly; hence, the number of mapped reads to a single sequence is only an indication of its redundancy. On the other hand, if all sequences of a sequence family or class are taken together, the total number of mapped reads (in respect to total genomic reads) reveals the effective redundancy of that family or class.

To establish the mapping parameters, 16 olive DNA sequences were selected, whose copy numbers per haploid genome were reported in the literature or were established by slot blot and hybridization experiments previously performed in our lab (supplementary material S1, Supplementary Material online). Mapping on sequences with known redundancy was performed using CLC-BIO with diverse parameters (mismatch cost, deletion cost, insertion cost, length fraction, similarity) and the significance of the correlation between copy number (as determined by slot blot) and average coverage for all 16 sequences was calculated for each set of parameters (see supplementary material S2, Supplementary Material online).

The parameters determining the largest correlation were selected to be used in the subsequent mapping of the WGSAS. After mapping, the WGSAS was subdivided into two classes of redundancy, redundant contigs (RCs) and nonredundant contigs (NRCs), using an arbitrary threshold of 16.2. RCs were further subdivided into highly and medium redundant (HR and MR, respectively) according to their average coverage (>1,620 and comprised between 16.2 and 1,620, respectively).

### Annotation of RCs

Annotation of supercontigs and individual contigs of the WGSAS was performed in two steps. In the first, sequences were masked by RepeatMasker (using as parameters –s –x –no_is –nolow) against the two libraries produced by the graph-based clustering method (see above) and against the RepBase database (Jurka 2000). In the second step, the remaining supercontigs and individual contigs were searched for homologies using the National Center for Biotechnology Information (NCBI) BLAST with an e value cutoff of 1 e^−6^.

In rare cases of ambiguity (i.e., supercontigs containing both tandem repeats and transposons fragments), the supercontigs were removed.

### Preparation, Sequencing, and Annotation of a Small Insert Library

Five micrograms of olive genomic DNA were sheared by Hydroshear (Genomics Solutions) in fragments between 1.5 and 3 kb and the inserts cloned using pPCR-Script Amp SK(+) (Stratagene) according to the manufacturer’s instructions. One microliter of the ligation mix was electroporated into *Escherichia coli* ElectroMAX DH10B Cells (mcrA, mcrB, mcrC, mrr; Invitrogen), using the BioRad GenePulser II electroporator, in a 0.1-cm cuvette at the conditions of 2.0 kV, 200 Ω, 25 µF. The average insert size after cloning was ∼2 kb, and inserts from 3,213 clones were selected for sequencing from both directions.

DNA for sequencing was prepared from selected transformants using the Montage Plasmid Miniprep Kit (Millipore). DNA sequencing was performed using an Applied Biosystems 3730 DNA Analyzer, the BigDye Terminator v3.1 Cycle Sequencing Kit (Applied Biosystems), and standard M13 forward and M13 reverse primers.

All sequences were annotated as above. Then, all sequences were compared with each other to detect additional repetitive sequences that did not show significant similarity to known repeated sequences but did overlap to each other by using the CAP3 sequence assembler (Huang and Madan 1999) using parameter settings of 90% sequence identity and 40 bp minimum overlap.

### Sequence Analyses of Tandem Repeats

One hundred sequences for each of the five main repeat families plus the Oe51 family were selected from 454 reads as follows: a preliminary consensus sequence of each tandem repeat type was deduced by dot plot analysis of the contigs assembled by RepeatExplorer and the subsequent alignment of the repeat units using CLC-BIO with default parameters. Then, a large set of 454 reads (1.0 genome equivalent) were subjected to BLASTN (with an e-value cutoff of 10^−10^) against the consensus, and the 100 most similar reads (i.e., “real” sequences) were selected for each type. Whenever more than one repeat was found within a read, only the most similar to the consensus was selected, that is each selected unit belonged to a different read, which represent a different locus.

Selected sequences were aligned using ClustalX 2.1 (Thompson et al. 1997) with default parameters. Then, 100 versions of the original multialignment were generated, and a distance tree was produced by neighbor joining analysis. The tree was visualized using FigTree (http://tree.bio.ed.ac.uk/software/figtree/, last accessed April 1, 2014).

Alignments were also used to perform statistics of intraspecific polymorphism within tandem repeat type, using the DnaSP program version 3.51 (Rozas and Rozas 1999). Nucleotide diversity, that is, the average number of nucleotide differences per site (π, Nei 1987) and its sampling standard deviation were calculated. The adopted procedure should exclude bias in the selection of sequences that could have affected the level of heterogeneity within each family, having in each case selected the 100 sequences most similar to the consensus.

### Reconstruction and Analysis of Full-Length LTR-Retrotransposon Sequences

Near-complete consensus sequences of long terminal repeat (LTR)-retrotransposons belonging to different clusters (as produced by RepeatExplorer using a set of 454 reads and annotated as *Gypsy* or *Copia* retrotransposons) were obtained, whenever possible, aligning contigs belonging to the very same cluster. For each cluster, the largest contigs and those showing the maximum read depth were subjected to a further CAP3 assembly, and the assembled supercontigs were analyzed to isolate putative full length elements.

The resulting sequences were subjected to dot-plot analysis to survey the occurrence of direct repeats, corresponding to putative LTRs. Sequences were also submitted to BLAST analysis (with an e-value cutoff of 10^−6^) against NCBI nonredundant database and to Pfam website (http://pfam.sanger.ac.uk/search, last accessed April 8, 2014) to identify retrotransposon domains. Putative retrotransposons were then annotated to separate LTRs from inter-LTR regions. Illumina reads were mapped to these retrotransposons, separately for LTR sequences from the respective inter-LTR region, and average coverage was calculated using CLC-BIO.

## Results

### Graph-Based Sequence Clustering of Olive Genome

The repetitive component of olive genome was initially studied using a sample of Illumina and 454 reads each corresponding to 1.0 genome equivalent, using RepeatExplorer (Novák et al. 2010). This tool allows de novo repeat identification, based on finding and quantifying similarities between individual sequence reads. This experimental approach produces separate clusters of frequently connected reads, automatically annotated, that represent individual families of repetitive elements.

**Fig. 1.—:**
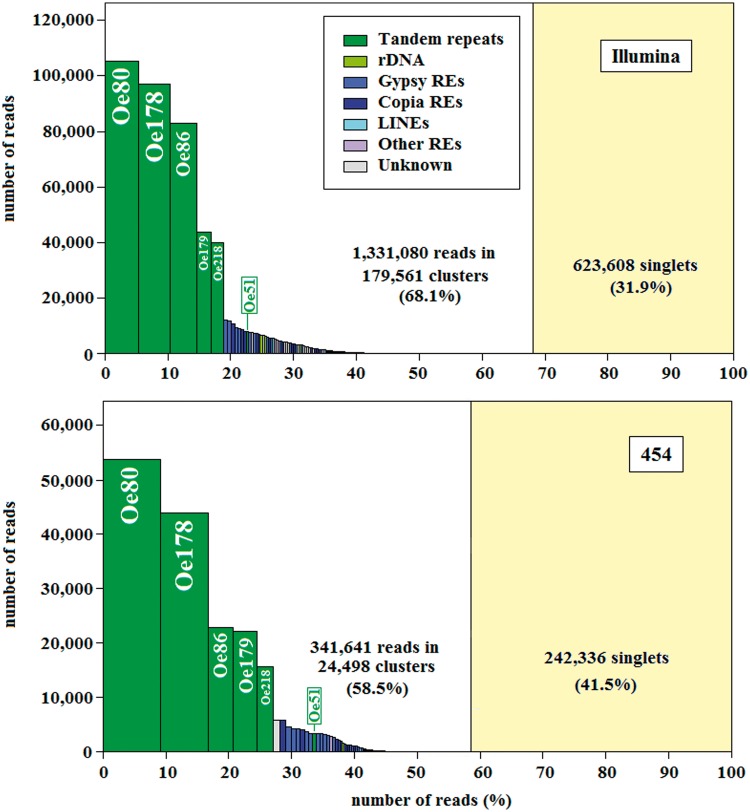
Repeat abundance based on one genome equivalent of Illumina (top) and 454 reads (bottom) clustered using RepeatExplorer (see Materials and Methods). Each bar in the histograms shows the individual size (height) of each cluster and the size relative to the total (width). The composition of each cluster is indicated by color, and single-copy, unclustered sequences are reflected to the right of the vertical bar. For the most redundant clusters, the annotation is reported within the bar.

A representation of the abundance of the clusters obtained by this analysis is reported in figure 1, keeping separated the Illumina and the 454 sets of sequences. The frequency of singletons should represent the low copy fraction of the genome, which resulted in 31.9% and 41.5% for Illumina and 454 reads, respectively. Both histograms clearly show five major clusters corresponding to five repeat families (fig. 1). Analyzing cluster graphs and contigs belonging to these families clearly indicated that they contained tandem repeats. The repeat unit of four of these families (Oe80, Oe86, Oe178, and Oe218) were already identified as tandem repeats (see the Introduction). The remaining family (Oe179) and a sixth minor family (Oe51) were also identified as tandem repeats by performing dot-plot analyses on 454 sequencing reads (i.e., “real” sequences, see supplementary material S3, Supplementary Material online). Besides clusters of tandem repeats, a number of minor clusters related mostly to *Gypsy* and *Copia* LTR-retrotransposons occurred (fig. 1). Different proportions of the various clusters were observed between the two sets of reads.

Based on graph clustering (fig. 1), we produced two sets of olive-repeated sequences. The first set contained tandem repeat units, isolated from contigs of the clusters corresponding to Oe80, Oe178, Oe86, Oe218, Oe179, and Oe51. One hundred sequences were collected for each cluster.

The second set was made of all contigs (3,152) included in the 192 clusters produced by RepeatExplorer and annotated by this tool as retrotransposons, DNA transposons, rDNA, and other repeat classes. Both sequence sets were used in subsequent annotation of olive-assembled sequences.

### De Novo Assembly of Genomic DNA

**Fig. 2.—:**
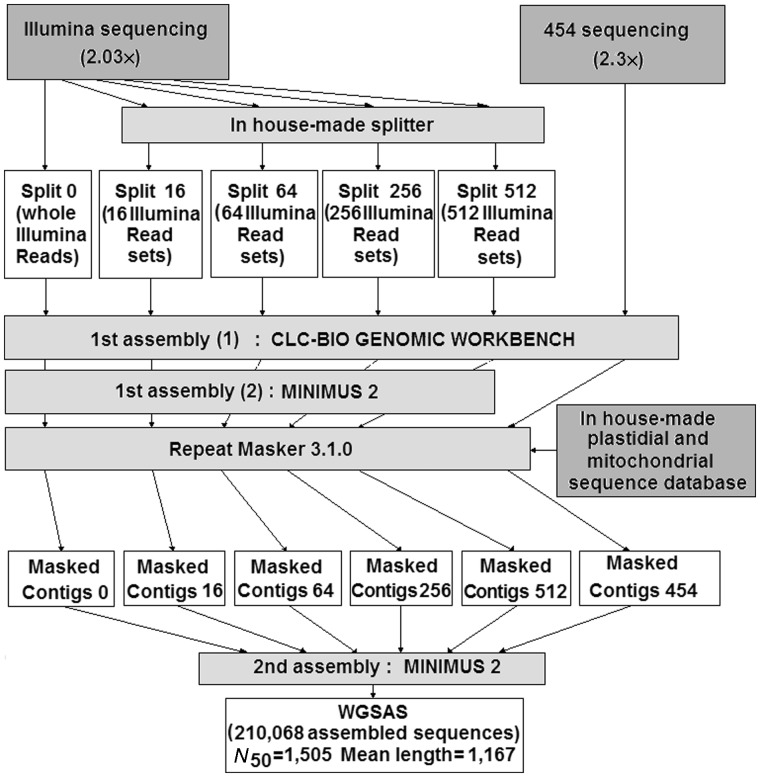
The assembly pipeline followed in these experiments to obtain a WGSAS.

A de novo assembly procedure was used to produce a large set of genomic sequences from Illumina (with or without splitting of read packages) and 454 reads. The assembly pipeline is reported in figure 2. Downsizing the Illumina package of reads resulted in the production of a lower number of contigs; however, major splittings allowed recovering the most redundant sequences (table 1).

**Table 1:** Characteristics of Assembled Sequence Sets Obtained by CLC-BIO Genomic Workbench and Minimus 2 Assemblies after Different Splitting of Illumina Reads

Split	Number of Subpackages	Subpackage Coverage	Number of Assembled Supercontigs^a^	Mean Length	Mean Number of Mapped Reads	Average Coverage	*N*_50_
0	1	2.03×	106,364	281.6	852.7	293.5	315
16	16	0.13×	104,468	255.6	924.7	288.3	266
64	64	0.03×	56,610	281.8	1601.0	645.9	299
256	256	0.008×	29,825	301.1	2809.9	708.3	342
512	512	0.004×	28,343	278.3	2873.9	779.6	313

^a^Supercontigs are contigs (as assembled by CLC-BIO) assembled to other contigs by Minimus 2.

Because of the large differences in average coverage among the various assembled sequence sets, a further assembly was performed to produce a comprehensive genomic sequence set for olive, including supercontigs and contigs obtained for each Illumina package (table 2).

**Table 2:** Statistics of Partial Assemblies and of Final Assembly (WGSAS)

Assembly	Number of Assembled Supercontigs and Contigs^a^	Mean Length (nt)	Contig Length Range (nt)	*N*_50_
Illumina (Split 0)	1,564,223	177.3	80–30,891	190
Illumina (Split 16)	592,971	155.2	80–22,320	164
Illumina (Split 64)	354,698	159.9	80–7,439	174
Illumina (Split 256)	289,536	162.6	80–4,768	181
Illumina (Split 512)	287,453	159.8	80–4,529	178
Illumina (Total)^b^	1,949,661	195.6	80–30,891	214
454	1,096,975	445.0	80–12,778	723
454 + Illumina	123,849	798.4	80–12,208	1,180
WGSAS	210,068	1,167.0	80–30,891	1,505

^a^Supercontigs are contigs (as assembled by CLC-BIO) assembled to other contigs by Minimus 2; contigs are sequences assembled by CLC-BIO that resulted as singleton after Minimus 2 assembly.

^b^Made by assembling contigs obtained with differently sized packages of Illumina reads.

The assembly of 454 reads produced 1,096,975 contigs (table 2). The final assembly produced 123,849 contigs (table 2). To obtain a more complete genome data set, also singletons longer than 1,000 nt were included. The resulting WGSAS was composed of 210,068 sequences (table 2).

### Estimation of Average Coverage of Assembled Contigs

Assuming that Illumina sequence reads in our experiments are sampled without bias for particular sequence types, mapping Illumina reads onto the WGSAS provides a method of estimating the redundancy of any genomic sequence in the data set (Swaminathan et al. 2007; Tenaillon et al. 2011; Natali et al. 2013).

**Fig. 3.—:**
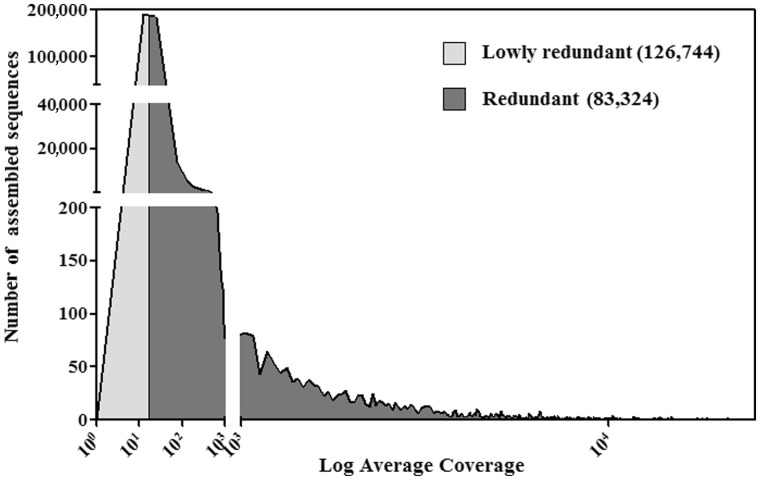
Distribution of mapped reads in the final assembly of the olive whole-genome database. Sequences were subdivided into redundant (average coverage > 16.2) and nonredundant (average coverage < 16.2).

A total of 8.1 genome equivalents of Illumina reads were mapped onto the WGSAS, assigning reads that map to multiple contigs randomly to one of the possible contigs. The coverage distribution of the whole set of contigs is reported in figure 3. The expected average coverage for a single copy contig was 8.1. Consequently, using 2-fold, this average coverage as an arbitrary threshold, the WGSAS was subdivided into two classes, RCs (83,324 sequences that constitute a collection of olive-repeated sequences, hereafter called OLEAREP), and NRCs (126,744 sequences).

### Analysis of the Repetitive Fraction of WGSAS

**Fig. 4.—:**
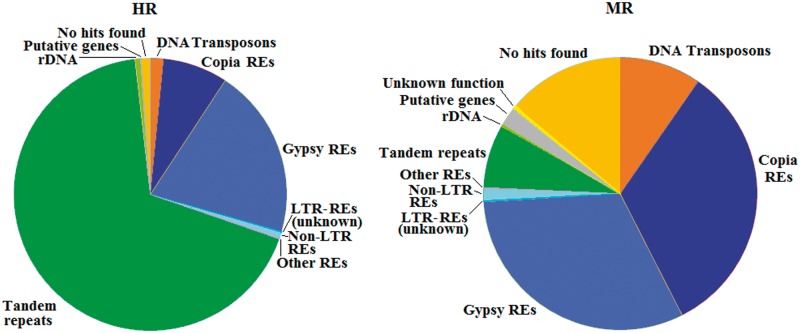
Sequence composition of the OLEAREP database (HR and MR sequences).

The frequency distribution of different sequence types in OLEAREP is reported in figure 4, in which RCs were further subdivided into two fractions, according to their average coverage, highly repeated (HR, 5,744 contigs), and medium repeated (MR, 80,727 contigs).

On the whole, 13.4% of RC did not find any hits in the graph-based clustering libraries and in the publicly available databases. Concerning the HR fraction, tandem repeats are the largest component, accounting around two-third of these contigs (fig. 4).

LTR-retrotransposons are also represented in the HR fraction, with *Gypsy* REs being more abundant in this fraction than *Copia* ones. Other classes of repeats (DNA transposons, rDNA, and putative genes) are present, though accounting only for minimal portions of HR set. Unclassified repeats represent only 1.1% of this genome fraction.

By converse, the MR fraction is mainly composed of LTR-retrotransposons (66.1%), with *Gypsy* and *Copia* REs showing similar percentages (fig. 4). Non-LTR retrotransposons are poorly represented, as frequently observed in plant genomes. Putative DNA transposons account for 9.65% of the MR fraction and represent a larger portion within MR than within HR fraction. All types of plant DNA transposons were found. Putative hAT and mutator elements resulted by far the most redundant in this class, followed by putative helitrons and CACTA elements. Tandem repeats are much less represented in this genome fraction than in HR. Finally, unclassified repeats account for 14.4% of the MR fraction.

Interestingly, a total of 1,759 contigs belonging to the repetitive fraction of the WGSAS showed similarity to putative protein encoding sequences. Of these, 1,747 sequences showed similarities to 685 different genes, and 12 were classified as encoding hypothetical proteins. Many of these protein encoding sequences occur in a few copies per haploid genome. In all those cases in which contigs showing similarity to protein encoding sequences have high average coverage, it can be possible that a gene or a gene fragment lies close to an unknown repeated sequence and they occupy the same contig, which consequently results redundant. In these cases, the redundancy should be related to sequences adjacent to those genes. By converse, when similarity to one and the same gene is found for a number of RCs, that gene should be really redundant. This was observed for 13 gene families, which showed sequence similarity with at least 15 sequences in the RC fraction (table 3).

**Table 3:** The Largest Gene Families Represented in the Olive WGSAS

Protein Encoded by the Gene Family	Number of Sequences
NBS-LRR disease resistance protein	176
Protein kinase domain-containing protein	75
Serine/threonine protein kinase	54
Pentatricopeptide repeat-containing protein	31
Cytochrome P450	25
NB-ARC disease resistance protein	22
Ankyrin	21
Tyrosine kinase	20
ABC transporter F family	20
WD40 repeat-containing protein	20
Myb transcription factor	17
Glycosyltransferase	15
Glycosyl hydrolase	15

The most redundant genes encode the nucleotide-binding-site-leucine-rich repeat (NBS-LRR) class of proteins, receptors that recognize highly variable pathogen effectors; their encoding genes are redundant in all sequenced plant genomes. Another large family is that encoding cytochrome P450, mostly related to catalyze the oxidation of organic substances and widespread in both prokaryotes and eukaryotes. Other proteins encoded by redundant gene families are the ATP-binding cassette (ABC) transporters that represent one of the largest and most ancient families in all extant phyla; pentatricopeptide repeats containing proteins, possibly involved RNA editing (Kotera et al. 2005); karyopherins, involved in transporting molecules between the cytoplasm and the nucleus of a eukaryotic cell.

Other redundant gene families found in OLEAREP are very heterogeneous; for example, those encoding proteins that share a common domain like protein kinases, involved in the transduction of signals to binding factors, centromeres, and other effectors; and glycosyltransferases, enzymes that are responsible for the biosynthesis of disaccharides, oligosaccharides, and polysaccharides. In all these cases, it is presumable that the gene regions encoding conserved domains, and not the entire gene sequences, are to be considered as redundant.

### The Composition of the Olive Genome

Being the WGSAS obtained by assembling Illumina and 454 reads, the simple composition of the database cannot offer a picture of the genome composition, because repeated sequences are assembled together and hence are underestimated. Consequently, we estimated olive genome composition in terms of redundancy by counting the number of reads that mapped to each sequence. Mapping results are summarized in table 4.

**Table 4:** Statistics of Mapping of Illumina Reads to the Whole-Sequence Data Set

	Sequence Data Set		Number of Reads	% of Genomic Reads
Matched genomic reads			124,445,343	89.70
	RC	HR	53,587,657	38.62
		MR	47,388,283	34.16
	NRC		23,469,403	16.92
Not matched genomic reads			14,296,611	10.30
Total genomic reads			138,741,954	100.00
Organellar reads			13,203,073	
Total			151,945,027	

On the basis of their similarity to the sequences in the organellar DNA database, we estimated that around 13.2 million reads were of organellar DNA origin. Considering the nuclear reads, 10.3% did not map onto any assembled sequence, indicating that the WGSAS does not cover the whole genome, as expected, having assembled only a maximum of 2.3 genome equivalents. It can be presumed that missing sequences are single or low copy-number sequences that the used genome coverage did not allow assembling such loci. In fact, the WGSAS is made of 245,159,848 nt, that is, around 16% of the length of olive haploid genome (1.5 Gb), although WGSAS is matched by 89.70% of the Illumina reads. On the other hand, it is also possible that stringent assembly procedures and shorter reads affecting alignment stringency and insufficient overlap have contributed to an increase in the number of unaligned reads. Moreover, some of the unmapped reads could also represent low-quality sequences containing a high proportion of errors that have not been trimmed adequately.

We considered the percentage of Illumina reads that match to a sequence class as an indicator of the proportion of that sequence class in the olive genome. So, it was estimated that the percentage of HR sequences in the *Olea* genome is very high, amounting to 38.62% (see table 4) at least. MR sequences account at least for 34.16% of the genome, and NRC sequences represent only 16.92% of the olive genome.

Olive genome composition was also estimated in terms of repeat types by mapping Illumina reads onto WGSAS as described above. The frequencies of each repeat type are reported in table 5. Tandem repeat sequences (excluding rDNA) account for 31.16% of the reads matching the WGSAS. LTR-retrotransposons amount to 38.84%, with *Gypsy* elements prevailing over *Copia* ones. DNA transposons and non-LTR retrotransposons show low percentages.

**Table 5:** Percentage Distribution of Repeat Classes in the Olive Genome

Sequence type	Order	Superfamily		Number of Contigs	Number of Matched Reads	Percentage
Retrotransposons	Unclassified			42	34,017	0.025
(Class I)	LTR	*Copia*		54,110	24,725,640	17.821
		*Gypsy*		47,920	28,884,342	20.819
		Retrovirus		101	74,960	0.054
		Endogenous retrovirus		4	6,314	0.005
		Solo-LTR		52	18,355	0.013
		Unknown		189	174,016	0.125
	LINE	L1		2384	1,739,119	1.253
		RTE		453	123,845	0.089
		Unknown		38	20,591	0.015
	Short-interspersed elements	tRNA		268	64,093	0.046
	Total					40.265
DNA transposons	Unclassified			67	32,668	0.024
(Class II, subclass I)	TIR	Tc1-Mariner		217	74,711	0.054
		hAT		7,187	2,784,674	2.007
		Mutator		5,790	3,335,678	2.404
		PiggyBac		1	34	0.000
		PIF-Harbinger		754	250,771	0.181
		CACTA		1,212	496,957	0.358
	Crypton	Crypton		7	2,054	0.001
(ClassII, subclass II)	Helitron	Helitron		1,297	672,682	0.485
	Total					5.514
Tandem repeats				11,260	43,233,770	31.161
rDNA				356	1,932,081	1.393
Unknown				308	179,225	0.129
No hits found				74,292	14,584,090	10.512
Total reads excluding organellar ones					138,741,954	

### Analysis of a Sanger-Sequenced Short Insert Library

A short insert library was sequenced using the Sanger procedure. It was composed of 6,408 sequences for a total of 5,793,980 bp, corresponding to 0.004 genome equivalents, with an average GC content of 37.5%.

Despite its limited size, this library can be considered as a sample of the olive genome. Its composition confirms the results obtained assembling the NGS reads on the abundance of olive repetitive fraction (table 6). Tandem repeats amounted to 24.16% of the sample, that is, they confirmed as a major component of the olive genome. Probably, the discrepancy between the percentage value of tandem repeats and that found in NGS experiments is an effect related to the small size of the sample used or to different biases either in the cloning procedure or in the Illumina procedure.

**Table 6:** Composition of the Sanger-Sequenced Small Insert Library

Sequence Type		Order/Superfamily	Number of Sequences	Percentage
DNA Transposons	Unclassified		4	0.06
	Subclass I		321	5.16
	Subclass II		49	0.79
	Total		374	6.01
Retrotransposons	Unclassified		1	0.02
		LTR/*Copia*	1,110	17.83
		LTR/*Gypsy*	1,277	20.51
		LTR/retrovirus	32	0.51
		LINE	59	0.95
	Total		2,479	39.82
Tandem repeats			1,504	24.16
rDNA			103	1.65
Similarity to genes			513	8.24
Unknown repeats^a^			80	1.31
Unknown			36	0.56
No hits found			1,137	18.26
Total nuclear genomic sequences			6,226	
Chloroplast			149	
Mitochondrion			33	
Total sequences			6,408	

^a^Unknown sequences that are assembled using CAP3 (see text).

Approximately 40% showed similarity to known transposable elements, mostly belonging to class I retrotransposons. Within this class, LTR-retrotransposons dominated with a prevalence of *Gypsy*-type elements over *Copia*-type ones, confirming results obtained by mapping Illumina reads. Non-LTR elements such as long-interspersed elements (LINEs) accounted only for 0.95% of all sequences. Class II elements, that is, DNA transposons, corresponded to 6.01% of the sequences in the library. Ribosomal DNA repeats amounted to 1.65% of the library.

A number of sequences (80, 1.31%) were recognized as repetitive by virtue of their similarity to at least another uncharacterized olive sequence within the short insert library but did not show similarity to previously described repetitive elements and were therefore classified as unknown repeats. These sequences raised the total fraction of sequences that can be classified as repetitive using a computational approach to 72.95% (in this sample).

Among the remaining nuclear genomic sequences of the short insert library, 8.24% showed significant similarity to previously described genes and 18.81% could not be classified into any of the previously described classes.

The Sanger-sequenced small insert library was also used for evaluating the assembly quality of the WGSAS, comparing a number of sequences of the small insert library to contigs of the WGSAS. Many reads of the small insert library were found in contigs with minor DNA sequence variations (supplementary material S4, Supplementary Material online). Alignments cover 14,807 nt, of which only 312 (2.11%) are mismatches, and 148 (1.00%) are indels.

### Analysis of Tandem Repeats

**Fig. 5.—:**
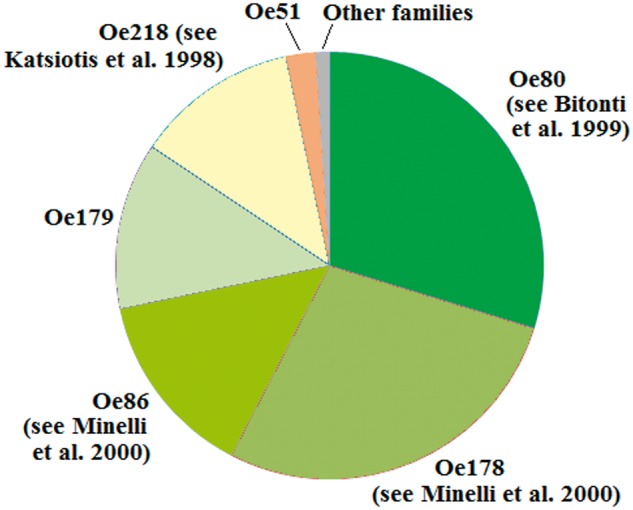
Composition of the tandem repeat class in the olive genome, based on the number of Illumina reads that map to the OLEAREP database.

Olive tandem repeats belong to six major families, defined according to their sequence and length (table 7 and fig. 5). The first three families (Oe80, Oe178, and Oe86) correspond to the OeTaq80, OeTaq178, and OeGEM86 families described by Bitonti et al. (1999) and Minelli et al. (2000) and account for ∼72% of tandem repeats. The fourth family (Oe179) was for the first time identified in this survey: it represents 12.6% of the tandem repeats and the most common repeat unit is 179-bp long; within this family, a number of repeats resulted truncated, with a variable length. In some cases, truncated elements were also arranged in repeat arrays, suggesting that the truncation has occurred while Oe179 was still replicating, with the truncated units that have continued their amplification.

**Table 7:** Characteristics of the Main Tandem Repeat Families Observed in the Olive Genome

Repeat Family	Already Known as	Length (nt)	GC Content (%)	Estimated % in the Genome^a^
Oe80	OeTaq80	80	45.4	10.33
Oe178	OeTaq178	178	43.2	9.69
Oe86	OeGEM86	86	36.0	4.91
Oe179	Not known	179	36.0	4.39
Oe218	pOS218	218	41.8	4.29
Oe51	Not known	51	33.5	0.78

^a^According to the number of matching Illumina reads.

The fifth family is Oe218, already described by Katsiotis et al. (1998), and accounting for 12.3% of tandem repeats. The sixth major family was observed for the first time in this survey, representing only 2.2% of the tandem repeats; the repeat unit is 51-bp long, and analysis performed on sequences of the short insert library (that are longer than Illumina and 454 reads) showed that this tandem repeat is usually linked to a *Gypsy* retroelement.

We have calculated the mean GC content of the whole genome as based on 454, Illumina and Sanger sequencing (35.0%, 38.0%, and 37.5%, respectively). Considering tandem repeats, Oe80, Oe178, and Oe218 constitute GC-rich, heavy satellites, having a GC content of 45.4, 43.2, and 41.8%, respectively. By converse, Oe51 shows a GC content of 33.5%, constituting a light satellite. The GC contents of Oe86 and Oe179 (36.0 for each type) are similar to the mean GC content.

All repeat families are present in multiple distinct contigs, indicating that distinct subtypes and higher-order structures of these sequences are present in the olive genome.

**Fig. 6.—:**
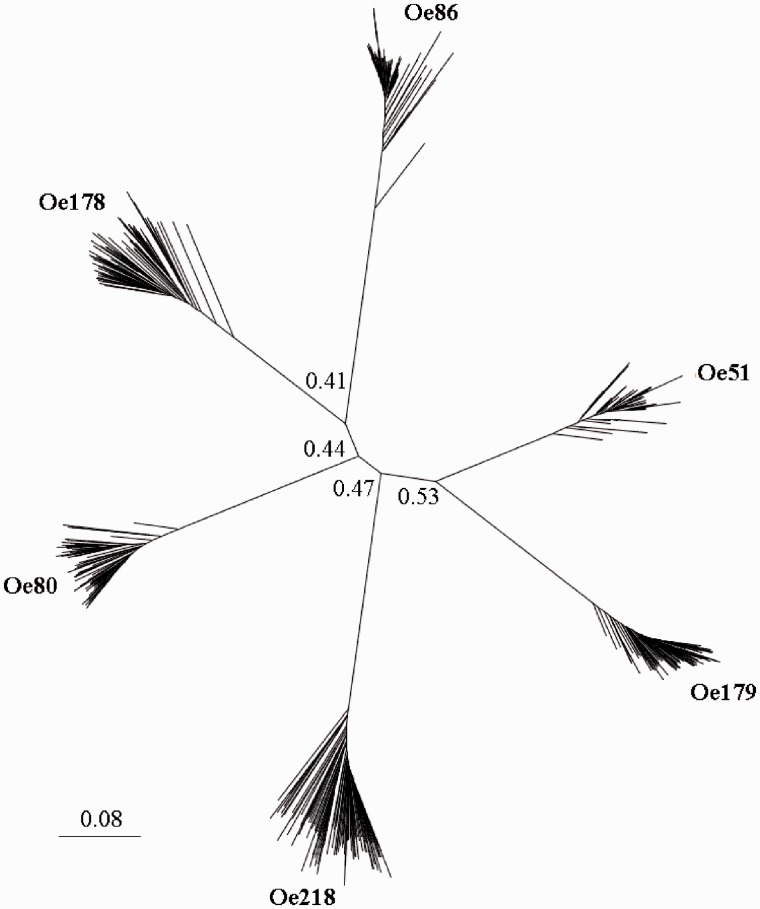
Distance tree of olive tandem repeats (100 sequences per family); bootstrap values higher than 0.4 are shown. Bar represents the nucleotide distance.

**Fig. 7.—:**
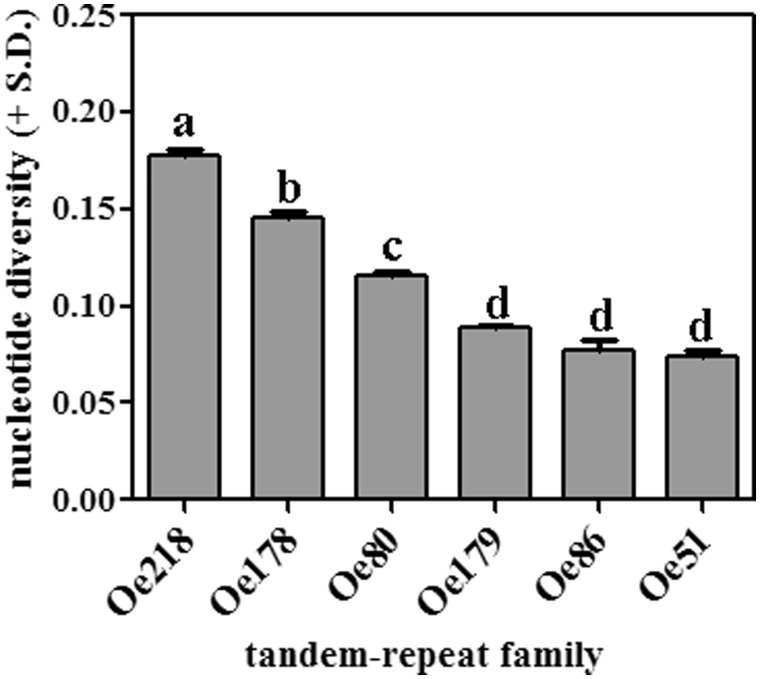
Nucleotide diversity (the number of nucleotide substitutions per site) of six tandem repeat families, calculated aligning 100 “real” sequences per family (the 100 sequences most similar to the consensus). Histograms labeled with the same letter are not significantly different (*P* > 0.05).

A distance tree was constructed using 100 sequences for each of the six repeat types, to evaluate the relationship among tandem repeat families (fig. 6). The tree shows that tandem repeat families are quite separated. For each tandem repeat family, nucleotide diversity (the number of nucleotide substitutions per site) was calculated. Figure 7 shows that Oe 218 is the most variable, followed Oe178, and Oe80; minor variations are observed within Oe179, Oe86, and Oe51.

### Analysis of LTR-Retrotransposons

Concerning the two main superfamilies of LTR-retrotransposons, *Copia*-related contigs are more represented than *Gypsy* ones in the WGSAS, being 54,110 versus 47,920 (table 5). Mapping analysis showed, however, that *Gypsy* elements are more redundant than *Copia* ones, being mapped by 28,884,342 and 24,725,640 reads, respectively.

To estimate the equilibrium between retrotransposon replication and retrotransposon loss, we produced a sample of 26 reconstructed LTR-retrotransposons of olive (see supplementary material S5, Supplementary Material online) belonging to different clusters as obtained using RepeatExplorer. For each retrotransposon, the putative LTRs and all (or many) protein domains were identified. In some cases, only one complete LTR could be isolated, probably because the similarity between LTRs determined the assembly of reads to only one end of the retrotransposon. We mapped Illumina reads to this sample of reconstructed LTR-retrotransposons, keeping separated LTR sequences from the respective inter-LTR region. The results of mapping are reported in table 8.

**Table 8:** Average Coverage of a Sample of Olive LTR-Retrotransposons Measured Separately on LTR and Inter-LTR Regions

Superfamily	Cluster Number	Average Coverage		LTR to Inter-LTR Ratio
		LTR	Inter-LTR	
*Copia*	24	1320.5	3816.5	0.346
	39	7107.8	5380.4	1.321
	48	3161.2	3119.2	1.013
	63	1451.9	1668.7	0.870
	66	2874.1	2186.8	1.314
	72	3068.2	1570.2	1.954
	86	1557.3	2444.8	0.637
	90	418.3	1475.4	0.284
	102	1422.8	1348.1	1.055
	108	507.1	1101.8	0.460
	112	1414.5	917.7	1.541
	114	1306.8	1211.7	1.078
	142	1098.0	1096.2	1.002
	165	744.8	797.5	0.934
	172	409.1	561.3	0.729
	178	1148.4	652.8	1.759
	212	983.4	520.4	1.890
	213	674.7	450.8	1.497
	239	509.9	497.6	1.025
	262	343.4	418.3	0.821
	Mean			1.077
*Gypsy*	45	5434.3	3318.0	1.638
	69	3669.1	1455.0	2.522
	146	10393.6	914.3	11.368
	149	1338.2	2626.0	0.510
	157	38338.0	869.2	44.107
	180	1208.5	658.0	1.837
	Mean			10.330

It can be noted that the ratios between LTR and inter-LTR average coverage ranged from 0.284 to 44.107. If all retrotransposons belonging to one and the same family were intact, that is, composed of two LTRs and one inter-LTR region, the ratio should have been 2. Only for 3 out of 26 analysed LTR-Res, the ratio was higher than 2, indicating the occurrence of solo-LTRs only for a small number of RE families in the genome. Many LTR-REs had a ratio lower than 2, that is, the inter-LTRs region was more represented in the genome than the LTR. This result suggests the presence of different families that share, at least in part, the inter-LTRs region and show a higher level of sequence conservation of the pol protein coding domains. Interestingly, analyzing separately *Gypsy* and *Copia* elements, the mean ratio between LTR and inter-LTRs average coverage was higher than 2 only for *Gypsy* elements (three elements over six analyzed, table 8), indicating that unequal recombination has affected especially this retrotransposon superfamily.

Obviously, this analysis is limited to only 26 retroelements and only to six *Gypsy* retrotransposons, which makes it difficult to derive general rules. Future availability of the olive complete genome sequence will allow us to verify this larger attitude of *Gypsy* elements to unequal recombination.

## Discussion

### Production of a Set of Olive Repetitive Sequences

The amount of sequencing data used in our experiments cannot be sufficient for whole-genome assembly, but it enables representative sampling of elements present in a genome in multiple copies.

We used two computational methods for assembling reads, the graph-based clustering procedure by Novák et al. (2010), keeping separated Illumina and 454 reads, and an assembly of Illumina and 454 sequence reads by using CLC-BIO and Minimus 2 as assemblers. Because of the relatively low genome coverage of sequencing, most of the contigs that were obtained by both methods do not represent specific genomic loci; instead, they are probably composed of reads derived from multiple copies of repetitive elements, thus representing consensus sequences of genomic repeats (Novák et al. 2010). Although the exact form of this consensus does not necessarily occur in the genome, this representation of repetitive elements has been shown to be sufficiently accurate to enable amplification of the whole length repetitive elements using polymerase chain reaction (PCR) (Swaminathan et al. 2007). Moreover, the comparison with available Sanger sequences indicated a good correspondence between virtual and real sequences.

Graph-based clustering showed the occurrence of five major clusters that we identified as tandem repeat families by sequence similarity search and graph and structural analysis. Four out of five tandem repeat families were already described in the literature; the remaining family, Oe179, was for the first time identified in this work. One minor repeat family Oe51 that accounts for 0.78% of the genome was also identified in this work.

Illumina and 454 reads gave similar results with regard to the identification of the five major clusters, but different results were obtained as to their redundancy in the genome that—for the first five clusters—amounted to 27.1% and 18.9% of the reads, respectively. Moreover large differences were observed for the minor clusters. These discrepancies between Illumina and 454 sequences can be attributed to the different length of the reads. The short Illumina reads seem most suitable for the quantification of the redundancy of a cluster, while the relatively long 454 reads should allow a more precise assembly, reducing the occurrence of chimeric sequences.

In the first step of the assembly procedure, we used two different packages of reads (Illumina and 454) that, in the case of Illumina reads, were subdivided into subpackages, with different coverages, before assembling them, a procedure already used for a study on the sunflower genome (Natali et al. 2013). The analysis of the assembled contigs clearly showed that splitting the original packages of Illumina reads into a number of subpackages resulted in the production of more repetitive contigs, although the number of assembled contigs was lower than that obtained by the assembly of the whole set of reads. Splitting the packages of reads did not apparently affect the mean length and the *N*_50_ of the assembled contigs.

The different features of the assembled sequence sets obtained by using read packages of different size, suggested us that the simultaneous assembly of split and unsplit packages could provide a more complete picture of the genome and of its components.

The assembly of 454 reads produced longer contigs compared to Illumina reads, as expected, because of the superior length of reads. In fact, in longer sequences, the occurrence of multireads is naturally reduced. In this case we did not proceed to a preliminary splitting of the read set; we preferred to perform a further assembly using both Illumina and 454 assembled sequences, obtaining a WGSAS. The quality of the assembly procedure was assessed by comparing sequences of the WGSAS to a Sanger-sequenced short insert library, in which sequences were real; despite the necessarily limited number of sequences in the small insert library, we found many sequences with high similarity to assembled sequences of the WGSAS.

We annotated the repetitive component of the WGSAS according to sequence similarity by searching in public databases and in two libraries based on sequences clustered and annotated by RepeatExplorer. This allowed annotating 86.6% of the repetitive component of the genome, a percentage larger than expected, dealing with poorly sequenced species.

The OLEAREP database, made of 83,324 repetitive contigs, gives a precise characterization of the repetitive component of the *O. europaea* genome. It includes all already known olive repetitive sequences but also new, unknown sequences with high redundancy, which might represent new repeats to be still identified and characterized.

### The Structure of Olive Genome

The olive genome shows the occurrence of DNA satellites in the form of tandemly arranged repeats that account for ∼31% of the olive genome, according to the mapping of a large set of Illumina reads on the WGSAS. When the frequency of tandem repeats in the genome was established by other methods (graph-based clustering of Illumina and 454 reads, and sequencing of a small insert library), this frequency resulted lower (27.69%, 19.30%, and 24.16%, respectively).

The short insert library, accounting for only 0.004 genome equivalents, can be subjected to sampling errors. On the other hand, the occurrence of large amounts of satellite DNAs is a limiting factor for graph-based clustering (Novák et al. 2013). In fact the number of reads that can be processed depends on the number of similarities they produce, because all read overlaps are to be loaded into the computer memory during the graph-based clustering process. In the case of satellite DNAs, whose repeated units have highly conserved sequences and occur in the genome in millions of copies, this results in a rapid saturation of the computer memory and, consequently, a minor precision in producing and quantifying clusters (Novák et al. 2013). For this reason, we concluded that the frequency of tandem repeats obtained by mapping Illumina reads to the WGSAS is probably the best estimation of the occurrence of satellite DNAs in this genome.

Major tandem repeat families identified in the olive genome show low sequence similarity, suggesting an independent origin from each other. The occurrence of different units in many distinct contigs (11,260 in total), that could not be assembled in one unit, shows the diversification of repeat unit in one and the same family, a common feature of tandem repeats that was already highlighted for Oe80. Bitonti et al. (1999) calculated only 76% sequence similarity among OeTaq80 repeat units. Tandem repeats are characterized by large instability, depending on the repeat unit length, on the purity (i.e., similarity) of repeats, on the base composition, on external factors such as biotic and abiotic stresses (Wierdl et al. 1996; Rosenberg 2001; Gragg et al. 2002; Schmidt and Mitter 2004; Legendre et al. 2007; Gemayel et al. 2012). Moreover, the mutation rate in tandem repeats is estimated between 10^−3^ and 10^−^^6^ per cellular generation (Verstrepen et al. 2005). Such a high mutation rate should be related to the hypermethylation of these sequences (see e.g., Hu et al. 2012). The different tandem repeat families of olive showed different sequence heterogeneity; analysis of nucleotide diversity indicates that Oe218, Oe178, and Oe80 are the least uniform.

The large fraction of genome formed by tandem repeats is a peculiar feature of the olive genome. In many studies on genome assembly, tandem repeats are preliminarily removed, representing a negligible fraction of the genome and facilitating the assembly procedure (see e.g., for the sunflower genome, Staton et al. 2012). Until today, the largest fraction of tandem repeats found in a plant genome was estimated around 23% in the genome of cucumber (Huang et al. 2009).

Different models describe the mechanisms by which tandem repeats expand or reduce in a genome (Tachida and Iizuka 1992; Paques et al. 1998; Richard and Paques 2000). Strand-slippage replication, also known as slipped-strand mispairing, or DNA slippage occurs during replication of the tandem repeat DNA when there is mispairing between the template and nascent DNA strands (Gemayel et al. 2010). Another mechanism involves DNA strand-breakage repair (Paques et al. 1998; Verstrepen et al. 2005), but the precise molecular mechanism of slippage remains unclear (Gemayel et al. 2010, 2012).

It is hypothesized that tandem repeats have a role in the genome. Besides their structural role in participating in centromeres and telomeres (Gemayel et al. 2010), tandem repeats can accumulate and generate intercalary heterochromatic regions. For example, in maize, tandem repeats form chromosomal knobs that reduce recombination rate in adjacent regions (see Ghaffari et al. 2013).

On the whole, the olive genome is made of ∼70% repeated sequences, largely represented by just five tandem repeat families. The other repeated sequences are mostly LTR-retrotransposons.

The ratio between *Gypsy* and *Copia* retrotransposon frequencies amounted to 1.17. This ratio is generally species specific. *Gypsy* to *Copia* frequency ratio is high in papaya (5:1, Ming et al. 2008), *Sorghum* (4:1, Paterson et al. 2009), rice (3:1 International Rice Genome Sequencing Project 2005), and sunflower (2.3:1, Cavallini et al. 2010) genome. In grapevine, an opposite trend was found, with *Copia* elements 2-fold more represented than *Gypsy* ones (Jaillon et al. 2007). Finally, in maize (Meyers et al. 2001) and poplar (Cossu et al. 2012), a similar abundance of the two superfamilies was observed as in olive.

In olive, *Gypsy* elements are slightly more abundant than *Copia* ones, in terms of frequency (20.82 vs. 17.82%), although a larger number of *Copia*- than *Gypsy*-related sequences was assembled in the WGSAS (54,110 *Copia* against 47,920 *Gypsy*). A larger number of *Gypsy*-related assembled sequences are found in the HR fraction of the genome, compared with *Copia*-related sequences, indicating that some families of *Gypsy* REs have undergone massive amplification during olive genome evolution.

This hypothesis will be tested when the complete genome of olive (or at least long sequences, as those of a bacterial artificial chromosome [BAC] library) will be available. In fact, the availability of complete LTR-retrotransposons allows dating retrotransposon insertion in the genome based on sequence divergence between LTRs (SanMiguel and Bennetzen 1998). The retrotransposon sequences reconstructed in our analyses are actually “virtual” sequences that could not correspond to specific loci, impeding their use for dating.

The frequency of retrotransposons in a genome depends not only on their amplification rate but also on their loss (Devos et al. 2002; Ma et al. 2004; Grover et al. 2008). DNA rearrangements, illegitimate recombination, and unequal homologous recombination drive DNA removal in plants by a number of mechanisms, as the repair of double-strand breaks (nonhomologous end-joining) and slipstrand mispairing (Kalendar et al. 2000; Ma and Bennetzen 2004; Neumann et al. 2006; Ammiraju et al. 2007; Hawkins et al. 2008; Morse et al. 2009).

To evaluate the extent of DNA loss occurring in the olive genome because of unequal recombination between LTRs, we estimated the frequency of LTRs and inter-LTR regions by mapping the large Illumina read set onto a sample of 26 full-length LTR-retrotransposon sequences reconstructed by alignment of contigs belonging to single clusters obtained by graph-based clustering (Novák et al. 2010). The results of mapping indicated that solo-LTRs do not appear to be an important fraction of any of the 20 tested *Copia* families. Even if only six *Gypsy* elements could be reconstructed and tested, the LTR/inter-LTRs ratios of these are generally higher than those of *Copia* elements. Two out of six *Gypsy* elements show very high ratios (44.11 and 11.37). This result strongly suggests the occurrence of numerous solo-LTRs for these two *Gypsy* families, although the occurrence of REs sharing LTRs but having different internal regions cannot be ruled out and could lead to an overestimation of solo-LTR frequencies.

Solo-LTRs are typically produced by unequal homologous recombination. Our data suggest that the high number of retrotransposons observed in the genome is obviously due to massive amplification of these elements. Genome size increase was however partly counterbalanced by substantial DNA loss, especially related to *Gypsy* elements, although in other studies solo-LTRs have been found especially in *Copia* elements (e.g., Cavallini et al. 2010; Staton et al. 2012). It is obvious that the availability of the complete genome sequence and consequently of a very large number of intact retroelements will allow us to validate this hypothesis.

In conclusion, our findings on olive genome evidenced the peculiarity of genome evolution in this species, with a very large fraction of the genome produced by tandem repeats amplification. The occurrence of a large and highly variable germoplasm for this species will allow to explore genetic variability concerning this genome fraction, possibly enabling to clarify the mechanisms by which such sequences have been produced and maintained during evolution and their function.

## Supplementary Material

Supplementary materials S1–S5 are available at *Genome Biology and Evolution* online (http://www.gbe.oxfordjournals.org/).

Supplementary Data
