# Supplementary material for: The Peculiar Landscape of Repetitive Sequences in the Olive (Olea europaea L.) Genome
Source: Genome Biol Evol. 2014 Mar 26;6(4):776–91. doi: 10.1093/gbe/evu058 (PMC4007544; doi:10.1093/gbe/evu058)
Supplement: Supplementary Data [file supp_6_4_776__index.html]

The peculiar landscape of repetitive sequences in the olive (Olea europaea L.) genome — The Peculiar Landscape of Repetitive Sequences in the Olive (Olea europaea L.) Genome — Supplementary Data 

# The Peculiar Landscape of Repetitive Sequences in the Olive (*Olea europaea* L.) Genome

## Supplementary Data

files

**Files in this Data Supplement:**

- Supplementary Data - pdf file
- Supplementary Data - pdf file
- Supplementary Data - pdf file
- Supplementary Data - pdf file
- Supplementary Data - pdf file
