# Supplementary material for: The Peculiar Landscape of Repetitive Sequences in the Olive (Olea europaea L.) Genome
Source: Genome Biol Evol. 2014 Mar 26;6(4):776–91. doi: 10.1093/gbe/evu058 (PMC4007544; doi:10.1093/gbe/evu058)
Supplement: Supplementary Data [file supp_evu058_Barghini_et_al_SupplMat_S1.pdf]

Supplementary Materials S1. Multifasta file with 16 olive sequences whose redundancy in the olive genome was previously determined by slot blot and hybridization by Natali et al (2007), Stergiou et al. (2002), and Giordani (unpublished).

```
>Oec01|AJ620478.2|#RLC
ACGACAGGTAGTGGCAGAACAATTGCTATTGTTAGAGAGATTCATACAGAGAAGAATCCA
ATTGATATGATGAAAAATTTAGTGACCGGAGAAAAGTTTGAGCTTTGTACCGAATTGGCT
GGCATGGATTCTAACTAATTTGTTAGAAAATAATTTCCTTCCTCAATGGGCTAGAGGGGA
AAATTGTTATAATGATCCAGCCCACTAAGCCTATTATAAGAATTAATTGGCCCAAATTTT
ATATTGTTTAATTAGATTAAAAATGGTGGTTATATATAAAAATACATCCCTCTCTCTCTT
GGCTGTTTTGTGTGAGAGAAATCTAATGGTCAACAACGGCAGCTAGAAGTTTTGAAAAAG
TTGTGGCTGTTGAGTGGAAATAGAGAGAGGAAGGACCGGCTGAAAGAGAAGAAAAATTGG
TGGCTGTTGGGGAATAAACTATTGCTACTAGAAAGAATTTTTTTTATTGAGGTGTGGAAT
CACAAATGTTATTTTTCTTCTCCATAATATATAAATTGGGAATTTCTCTAAAATATATTT
TGGATTTGTATTCAAAGTAGGTTCTTACAAGGTTGATCAAATTGCTGATTTGTTGTAAGA
TAAATCGTTGTCTTAGAGATAGATTATTGTAGTATTTCCAATTTGTGGAGAATTCAAGTT
TTGAGGCATAATCTTCAATATTGTAATCGATTTTAATTATTATAGTAGATTTTGACCAGG
GTTGCCCCGTGATTTTTTTCCCTTTACATTGGAGGGTTTTTTCCACGTAAAATTTGGTGT
TGCGTTTTCTTTTTTTTTGCTTGGATTATTTATTTACATAGTTGATCGTTCTAATTAATT
GCTTGGATTGGTAAATTTATTCTTGCAATAATTGAAATTGCCCAACAGCTCATTCCATTA
AGAAGTAATGTGTTGGTTAAGCAATTGATTAAATCAATGCTACCATTTTCTCAAAAGAAA
TTAAAGAAGGAAGAAGACATTTGATTCCAGGTAAATGGATAGTCTTCTTTTATTCAAAAT
TAAGCATGTGATGGCATTACAGAACTTTTTTGAAAAGAATCATCATTAAGTTTTCCTCTT
GAAAAAAATGGCAACTGGAAGTAATTGAATCGATTTATTTAAATTTCTTACCAGTTCTTG
CGGTTGTTTAATTTTCTGTGCCTTTGAATTGTCTCCTTACCAGTGGTTTACATGATGAAC
AAGAATAAAAATATTAGTTCCCCTTTTGATGGAAATCACACGTGATATTTTTCCTGACAG
AAACAAAAAGAAATATTTCTTGAACAATTTAATATGACGTAAAATTGGTTGATACGATTT
AGATTTTCATTTTCTTCAATTACTGTATGTCTTTGATCAAGCTAAAGAACAAACAAGAGG
TAGGTCGANTGGGCAATCCTAGAATTTTTTNATGAACCTGAATAATGGAATATTTTATTA
TTTGAGGGGTAAAAACAAGATTAGATGGGACTAGAGGAGGTACAACTGACTGAAGGCATT
GTATTGAAGATTTAATCCTTCAGTTTTATTATACAAGTTGCATTTTGTTTAAGCAAACGG
ATAAGAATTGCAGCTAGGGCTGCCTAGTGTAGGATTAGANAGGTGGACAATGGCTCCACC
TAACTTGCCCAGTCACATAACCCATGTTNGGCCAGACCAGGCATACAGGCAAATCCTNTC
CCTGTAGTGTTAAANAGTAAAATTTTACATTGTTTACATGCGAAAATCAAAATCAACATA
TAAAAGGCAAGACCAATCGAGTTGGTTTCATATAATGGCATGACCAATNTATATGAACTC
GAAGGTTTNTTGTAACTNTCAGAAAAAGAAGTGGCTTCATTCCATGTCTCATTTNTCTNT
TGTACAGGTTGTGGTATTGTAGCTTTACT

>Oec02|AJ620479.2|#RLC
ACGACAGGTAGTGGCAGAACAATTGATATTGTTAAGAAGATTCATAAAGAGAAGAATCCA
ATTGATACGATGACAAAGGTTGTGACCAACCAAAAGCTTGAGCTTTGTGCCAGACTGGCC
GGCATGAATTCTAACTAATTTGTTAAAGCATAGTCTCCTTCCTTAGTGGGCTGGAGGGGG
AGATTGTTATATGGGCTAACTTGGTCCAGCCCATGATGAAGACTGGCCCAAGCCAATATA
TTATTTAATTAAGTCCCACACACACACACACACACACACACACAAAGGCATTTTGCAACATG
TAAAAGGCAATATTCATTAGTTACATGTTGCTAAAAGGGGTCAATTGCAACATTCAGCAA
GTCTACGTTGCTATTGATTCACTAAATTGAGTTTGACGACATTTTTTTAATTAATGATGC
TAATAATGCTAATACACCCTTTTGCAATGTTCGGTGTCCATTGTCGTTAAATTTTATTTT
ATTATGGCAACGTGCAATGCATGTTCCCGTTAATTAGTTTTAGCAACAGGCAATGTACGT
TGCTATTAGATGTTTTACCAACAAACATTTGAACGCTGTAATTAGTGTTTTAGCTATGTG
CACTGCGCGTTGCCGTTAACCATTTTAACAACGCGCATATGAATGTTGCCATTAATCTTT
TTGCANCCTTTCCCTCAGTGTAATACAAAGGCAAAACAACTGGCAGAAANCCTAACTCTG
ATTT
```

```
>Oec04|AJ620480.2|#RLC
CTCGGACGAAGCACATTGATGTCAGGAATCATTTTGTGCGTGATGTTATAGAGAAAGGCG
ATATTTCACTCTCGAAGGTACACACAAATGAGAACCCAGCTGACATGTTAACCAAAGTGG
TGACGGGTAGCAAGTTTCAACATTACTTGGACTTGCTTCACATTAGTCCGTGTTGATATG
CCTGATATGCCCGAGAGGGCATGGCCCTTCGGGGCGAGGTTGGAATGAGGTGGAGAGTAT
ATGAAGGTGGAGGTGAACTACGGAGGCTTGATCCCGAGTTATGGGTACGTAGGCAATCAT
GGTTTGAATGATGCAGCCAAGTTGGTGCTTGCTACTTCTTCTTGTGTTCGTCCAAAATTT
GAGGTCTATCAAATGATGTACGAGGTGGAGAATTGTGAGAAGAGGCTGCTGGTTTCTGCT
GCTGGTTTTCTGCTGGGGCGCCCAGGCGCCTGGAGTGGGCGCCTAGGCGCCCCGGTAGGC
TATATATAGCCATT

>Oec05|AJ620481.2|#RLC
CTCGGACGAAGCACATTGATGTCAGATATACATTTGTTAGAGATAAAATTCTTGAAGGGA
AGTTGAAGATAAAATACATTCCATCATCAGACCAAACTGCAGATCTGTTAACCAAACCAC
TTGCTCAGTCCAAATTCTTGTTTCTCAAAGACAAGTTCGGAGTTAATGAATAAACTCCAG
CTTGATGAAAGATGTTAAAATGTTTACTAATAGCTGGTGACATACTCCAGTATGGGCGTG
TGTACTGGAATGGTCCTTTTACTTGTTTACCACTCTAGCACTTATGGAACCATAATTTAC
TGGTGTAACTGTACTTGTTCATCACATTTCTGTTTTTTATTTAATTTCAACTCTAAATGA
CGGTTTGTGATTTGACGCATTAATGTGATAGTTTTTTTTTTGCTTCATATATCAAATCAC
TAAATGTATGTGTATACTATGTACGGCGACCATGATAATGAGTGACTGCAAATAACATTA
CTCTTACCTATATTTACCTATATACGTGTTCTCTTCACCTCTCAAATTACTCCAATTATC
TCTTGAGTTAATGTATTATCGATGCTTATGTGAACTTTTCTGTCTCTTTCT

>Oec06|AJ620482.2|#RLC
CTCGGACGAAGCACATTGATGTCAGGTATCATATTGTGCGTGATGTTATAGAGAAAGGTG
ATATTTCACTCTCGAAAGTACACACAAATGAGAACCCAGCTGACATGTTAACCAAAGTGG
TGACGGGTAGCAAGATTCAACATCACTTGGACTTGCTTCACATTAGTCCGTGTTGATATG
CCTGATATGCCCGAGAGGGCATGGCCCTTCGGGGCGAGGTTGGAATGAGGTGGAGAGTAT
ATGAAGGTGGAGGTGAACTACGGAGGCTTGATCCCGAGTTATGGGTACGTAGGCAATCAT
GGTTTGAATGATGCAGCCAAGTTGGTGCTTGCTACTTCTTCTTGTGTTCGTCCAAAATTT
GAGGTCTATCAAATGATGTACGAGGTGGAGAATTGTGAGAAGAGGCTGCTGGTTTCTGCT
GCTGGTTTTTTCTGGGGCGCACAAACGCCTAGAGTGGGCGCCAAGGCGCCCCGGTAGGCG
ATAGATAGCCGACT

>Oec07|AJ620483.2|#RLC
CTCGGACGAAGCACATTGATGTCAGGTATATCATGTGCGTGATGTTATAGAGAAAGGTGT
TATCACACTCTCGAAGGTACACACAAACGAGAACCCAGCTGACATGATAACCAAAGTGGT
GACGGGTAGCAAGAATCAACATAACTCGGACTCGCTCCACATTAGACCGTGTTGATATGC
CTGATATGCCCGAGAGGGCACGGCCCTTCGGGGCGAGGTTTGGAATGAGATGGAGAGTAT
ATGAAGGTGGAGATGAACTACGGAGGCTTGATCCCGAGTTATGGGTACGTAGGCAATCAT
GGTTTGAATGATGCAGCCAAGATGGTGCTTGCTACTTCTTCTTGTGTTCGTCCAAAATTT
GAGGTCTATCAAATGATGTACGAGGTGGAGAATTGTGAGAAGAGGCTGCTGGTTTCTGCT
GCTGGTTTTTGCTTGGGCGCCCAGGCGCCTGAAGTGAGCGCCTACGCGCCCCGGTAGGCT
ATGTATAGCCATC

>Oec03|AJ536119.2|#RLC
ACGACAGGTAGTGGCAGAACAATTGATATTGTTGGAGAAGATTCACACTGAGAAAAATTC
AGTTGATATGATGACAAAGGTTGTGACTAGAGAAAAGTTTGAACTTTGTGCCGCACTGGT
CAGCATAGATTCTAACTAATCTGTTAGATGATAATATCCTTCTTCAATGGGTTGGAGGAG
GAGATTGTTATACGGGCTTGTCTAGCCCATGATGGAAAAAAGCCCAATCTTCTTTATTTT
AATTTAGGTCAACAAAAGTGGCCACACACACATTAAAAATCGTGGCTGGTTGTGCGAGAA
AGAGGCAAAAACAAGGACTGTTGTGAAGCAATTAAAAACTGTGGTTGCTATATTCATATG
```

GAAAAATGTTGACTAGGAAAAAGATAAAAGAAGAAGAAAACCTGGCTGCTCAAAAAAGAA
AAATAATTGGAAGGGAAGTGTTATTTATTTATTTAATTTTTTCAATCTTTAGGTGTGGGT
TCCCAAAAGGTCTCTCT

>Oec10|AJ620488.2|#RLC
ACGACAGGTAGTGGCAGAACAATAGACATTGTTAAGAAGATACATAAAGAGAAGAATCCA
ATAGATATGATGACAAAGGTAGTGACCAACCAAAAGCTTGAGCTTTGAGCCAGACTGGCC
GGCATGAATTCAAACTAATTTGTTAAAGCATAGTCTCCTTCCTTAGTGGGCCGGAGGGGG
AGATTGTTATATGGGCTAACTTGGTCCAGCCCATGATGAAGACTGGCCCAAGCCAATATA
TTATTTAATTAAGTCTCA

>Oec09|AJ620487.2|#RLC
ACGACAGGTAGTGGCAGAACAATAGAAATTGTTAAGAAGACACATAAAGAGAAGAATCCA
ATAGATACGATGACAAAGGCAGTGATCAACCAAAAGCTAGAGCTTTGTGCCAGACTGGCC
GGCATGAATTTAACTAATATGTTAAAGCATAGTCTCTTACTTAAGTGGGCTAAGGAAAAG
ATTGTTATATGGGCTAACTCGGTCCAGCCCATGATGAAGACTGGCCCAAGCCAATATATT
ATTTAATTAAGTTCTCACGCA

>Oec08|AJ620484.2|#RLC
ACGACAGGTAGTGGCAGAACAATAGATATTGTTAAGAAGATTCATAAAGAGAAGAATCCA
ATAGATATGATGACAAAGGATGTGACCAACCAAAAGCTTGAGCTTTGTGCCAGACTGGCC
GGCATGAATTCTAACTAATTTGTTAAAGCATAGTCTCCTTCCTTAGTGGGCTGGAGGGGG
AGATTGTTATATGGGCTAACTTGGTCCAGCCCATGATGAAGACTGGCCCAAGCCAATATA
TTATTTAATTAAGTCTCAC

>Toe15|AJ416432.1|#RLC
CACTGCGTTTCCTGCATGGGGACTTGGATGAGGAGATCTATATGCATCAACTTGAGGGCT
AACAAGGTTGAGGATAAGGAACGTCAGGTATGTCGCTTGAGAATGATCTATATGGATTGA
AGCAATCTCCTCGGCAGTGGTACAAGCGATTTGACTCCTTCATGCTAAAGCATGGTTTCT
CCAGAAGTGAGTATGACTGTTGTGTTTATATTCAAAAACTTCGGGAAGGTAACTATATCT
ATCTTTTTTTGTATGTAGATGACATGAT

>OeTaq178|AJ297958.1|#tandem_repeat
AAAGAATGAGCACTTTTATATTTGGAACGCTTGGATAGTCGGCGTGCGAAGAATTGAGCG
TAACTTTTGCACACGGCGTCCGTTTTGGGCCCATAAGTACTTTTTGGAAATGTAGCGACC
GTTTTGGATTGAACTCACAACTTCTTCGTGACGTTCGAGGGATTTTCGACCATTTTTCG

>OeGEM86|AJ297959.1|#tandem_repeat
TTTGTTCGTTTTTGGCGAATTGCTAGTCTAATTCGATTGGAATCGAGCCAATGAAAAGAG
ATTTTTGATTTCATGATAACTTCGGG

>OeTaq80|#tandem_repeat
GACAGGTTGATCGGGAACAAAAATCGCGCCGGGGGCAATTTCGTCATTTTTCTCGACTGT
GTCGGAATTGGCTCAAATTT

>actin mRNA|AY788899.1|#actin
CTGGCTGGTCGTGACCTTACTGATCACTTGATGAAAATCCTCACAGAGCGTGGTTACTCT
TTCACCACCTCTGCAGAGCGGGAAATTGTGAGAGACATGAAAGAAAAGTTAGCTTACATT
GCCCTTGACTATGAGCAGGAGCTTGAGACTTCAAAAACTAGCTCTTCTGTGGAGAAGAGC
TACGAGTTGCCTGATGGGCAGGTTATTACCATTGGCGCTGAGCGTTTTCGATGTCCTGAG
GTTCTTTACCAGCCTTCAATGATCGGAATGGAAGCAGCAGGCATTCACGAAACCACATAC
AACTCCATCATGAAGTGTGATGTGGATATTAGGAAGGACCTATACGGTAATATTGTACTT
AGTGGCGGTTCGACCATGTTTCCAGGTATTGCTGACAGAATGAGCAAGGAAATTACAGCG

CTAGCTC

>rDNA|AJ865373.1|#rDNA
GAATTCACCAAGTGTTGGATTGTTCACCCACCAATAGGGAACGTGAGCTGGGTTTAGACC
GTCGTGAGACAGGTTAGTTTTACCCTACTGATGACAGTATCGCAATAGTAATTCAACCTA
GTACGAGAGGAACCGTTGATTCGCACAATTGGTCATCGCGCTTGGTTGAAAAGCCAGTGG
CGCGAAGCTACCGTGCGCTGGATTATGACTGAACGCCTCTAAGTCAGAATCCGGGCTAGA
AGCGATGCGCGCACCCGCCGTCCGCTTGCCGACCCGCAGTAGGGGCCTTACGGCCCCCAG
GGGCACATGTCGTTGGCCAAGCCCTCGCAGCGGACGTGCTGCGTTGGCCGCCTTGAAGTA
CAATTTCCATCGAGCGGCGGGTAGAATCCTTTGCAGACGACTTAAATACGCGACGGGGTA
TTGTAAGCGGCAGAGTGGCCTTGCTGCCACGATCCGCTGAGATTCAGCCCTTTGTCGCTC
CGATTCGTCCCTCCCCTTCCGTTCCTCCCAATCTAAATCTTCTCTATCCGAAAATGCACT
GAGGCTAGAACGCCCATGGTCCTTGAAACAACTTTTCTTTTCAATGCCCAAGTGTGGAGG
GACAAAAACCCATGTACAGCAAAGCGTAGCATCCGTAGCTGTTACCAGCCTCTGCGCTAG
AGTTCATGCAGTGGACATAAAATATGTGATGTTTGGGTGCTCGATCTGTCGCCTTGGTAT
GACTGTGATATGCCTTTGGGTACTTGTGATATGCCTTCGTGCTCCTGCATTGTACTTGAG
TTGCTCCATTATTGTGCATGCGACGTCCATCAGTTGCGCAATGCGGAATTCGGATGCATG
AGATGTCCCTTAACATGCCTGGTGTGCCTGCGATTAGCATAAGCACACGGTGCCCAGAAT
TGCCTGAAAATCAGGCTGCGTCCCTGCGGTGGCCAGGACGCGTCCCGGACATGGCCTGCA
CACGGTGCCCAGAATTGCCTGAAAATCAAGCTGCGTCCCTGCGGTGGCCAGGACGCGTCC
CGGACAAGGCCTGCACACGGTGCCCAGAATTGCCTGAAAATCAGGCTGCGTCCCTGCAGT
GGTCAGGACGCGTCCTAGACATGGCCTGCACACCGTACACAGAAACTGCCCAGAATTGCC
TGAAAATCAGGCTGCGTCCCTGCAGTGGCCAGGACGCGTCCCGGACATGGCATGTCCCTG
CAGTGCCCAGAATTGCCTGAAAATCAGGCTGCGTCCCTGCAGTGGCCAGGACGCGTCCCG
AACATGGCCTGCACACGGTGCCCAGAATTGCCTGAAAATCAGGCTGCGTCCCTGCGGTGG
CCAGGACGCGTCCCGGACATGGCCTGCACACGGTGCCCAAAATTGCCTGAAAATCAGGCA
GCGTCCCTGCAGTGGTCAGGACGCGTCCCGGACATGGCCTGCACACCGTGCACAGAAACT
GCCCAGAATTGCCTGAAAATCAGGCTGCGTCCCTGCAGTGGCCAGGACGCGTCCCGGACA
TGGCCTGCACTCGGTGCCCAGAATTGCCTAAAAATCAGGCTGCGTCCCTGCCGTGGCCAG
GACGCGTCCCCGACATGGCCTGCACACGGTGCCCAGAATTGCCTGAAAATCAAGCTGCGT
CCCTACGGTGGCCAGGACGCGTCCCGGACATGGCCTGCACACGGTGCCCAGAATTGCCTG
AAAATCAGGCTGCGTCCCTGCGGTGGCCAGGACGCGTCCCGGACATGGCCTGCACACGGT
GCCCAGAATTGCCTGAAAATCAGGCTGCGTCCCTGCCGTGGCCAGGACGCGTCCCGGACA
TGGCCTGCACACGGTGCCCAGAATTGCCTGAAAATAAGGTTGCGTCCCTGCCGTGGTCAG
GACGTGTCCCGGACATGGCCTGCACACGGTGCCCAGAAACTGCCCAGAATTGCCTGAAAA
TCAGGCTGCGTCCCTGCAGTGGCCAGGACGCGTCCCGGACATGGCCTGCACTCGGTGCCC
AGAATTGCCTAAAAATCAGGCTGCGTCCCTGCCGTGGCCAGGACACGTCCCGGACATGGC
CTGCACACGGTGCCCAGAATTGCCTGAAAATCAAGCTGCGTCCCTGCGGTGGCCAGGACG
CGTCCCGGACATGGCCTGCACACGGTGCCCAGAATTGCCTGAAAATCAGGCTGCGTCCCT
GCGGTGGCCAGGACGCGTCCCGGACATGGCCTGCACACGGTGCCCAAAATTGCCTGAAAA
TCAGGCAGCGTCCCTGCAGTGGTCAGGACGCGTCCCGGACATGGCCTGCACACCGTGCAC
AGAAACTGCCCAGAATTGCCTGAAAATCAGGCTGCGTCCCTGCAGTGGCCAGGACGCGTC
CCGGACATGGCCTGCACTCGGTGCCCAGAATTGCCTAAAAATCAGGCTGCGTCCCTGCCG
TGGCCAGGACGCGTCCCGGACATGGCCTGCACACGGTGCCCAGAATTGCCTGAAAATCAA
GCTGCGTCCCTGCGGTGGCCAGGACGCGTCCCGGACATGGCCTGCACACGGTGCCCAGAA
TTGCCTGAAAATCAGGCTGCGTCCCTGCCGTGGCCAAGACGCGTCCCGGACATGGCCTGC
ACACGGTGCCCAGAATTGCCTGAAAATAAGGCTGCGTCCCTGCCGTGGTCAGGACGTGTC
CCGGACATGGCCTGCACACGGTGCCCAGAATTGCCTGAAAATTAGGCTGCGTCCCTGCAG
TGGCCAGGACGCGTCCCGGACATGGCCTGCACACCGTGCACAGAAACTGCCCAAAATTGC
CTGAAAATCTGGCTGCGTCCCTGCAGTGGCCAGGACGCGTCCCGGACATGGCCTGCACAC
GGTGCCCAGAATTGCCTGAAAATCAGGCTGCGTCCCTGCCGTGGCCAGGACGCGTCCCGG
ACATGGCCTGCACATGGTGCCCAGAATTGCCTGAAAATAAGGTTGCGTCCCTGCCGTGGT
CAGGACGTGTCCCGGACATGGCCTGCACACGGTGCCCAGAATTGCCTGAAAATTAGGCTG

CGTCCCTGCAGTGGCCAGGACGCGTCCCGGACATGGCCTGCACACCGTGCACAGAAACTG
CCCAAAATTGCCTGAAAATCAAGCTGCGTCCCTGCAATGGCCAGGACGCGTCCCGGACAT
GGCCTGCACACGGTGCCCAGAATTGCCTGAAAATCAGGCTGCGTCCCTGCCGTGGCCAGG
ACGTGTCCCGGACATGGCCTGCACACGGTGCCCAGAATTGCCTGAAAATTAGGCTGCTTC
CCTGCAGTGGCCAGGACGCGTCCCGGACATGGCCTGCACACCGTGCACAGAAACTGCCCA
GAATTGCCTGAAAATCGGGCTGCGTCCCTGCAGTGGCCAGGACGTGTCCTGGACATGGCC
TGCACACGGTGCCCAGAATTGCTTGAAAATCAGGCTGCGTCCCTGCAGTTGCCTGGACGT
GTCCCGGACATGGCCAGCACACGGTGCCCAGAATTGCATGAAAATCAGGCTGCGTCCCTG
CAGTGCCCGGGACTCGTCCCGGACATGGCCTGCACACAGTGCCCAAAAATGCCTGAAAAT
GAGGCTGCGTCCCTGCCGCGGCCAGGACACGTCCCGGACATGGCCTGCACTCGGTGCCCA
TAAATGCCTGAAAATAAGCCTCGCGATATTTTATCGATGGATGGTCGTCGGGAACCTAAG
ATGATCGCTCGACAGAAGTTTATCGTTGGAATTTACTACATTTCGAGTTGTTTCCACCGC
ATTTCACATTCCTCCCGCCCCCGTCCCCTGTTTCCGGTCATGCATCAAAATACGTGTGAA
TCGAAATTGTTTTCGACAAAAGAGCAATTGCTCCTCAAATATTTGATGATAACGAGCCTC
CCGTGATTTTTGGGGATTTTTCCCCGTCCGTCTTCCATTTTTTCCGATTTTTCCCTATTT
TTAATATTTTTTTAAATAAAAAATAAAAATATTTTTCGGTCGAAAATATTTTTTTCGAGA
CCACATTCAATCATACTTTCATCCCCAAAAAGAATTTTCCAAAAAATCATCACCGAAAAT
CCTACAGAAAATGGAAAAGAAATAGTATGATTCCTCAGCCCTTTGATATCTACTCCTGTC
ACTTGGGTTTTTTCTCAGAAGTCTATTATAGGGGGGAGGTGGTGCATGAAAGGGAACAAG
CAGCATGTGCAAGCTGCTGCAGGGAGCAACTGCCTACGGGTGGCTTGCGTCCCTGCAAAC
TTCGGAGCCTTGGGGATCGGCACATGCCGGCCCGGGCAAGTTTCCCGTCCGACGATGGTG
CTGCTCCGGCGATACTCCGGCCGGTGACCCCCCCGCAGCACCCGGACGGAACGCATGCGT
GGGCACGAAATCGCGTTGCGCGTCGAGAGATGGCCTCGGATTTCCCGGCAATTGCCTATT
TCGGTCCAGAAACGGAGTAAAGACCCGACAAGAGTGGGAGGAACAATCCGGACGGAGGGC
ATGCGCGGGGGCAAGAAACCGCGCGACGTGTCGATCGATTGGCCTAGGCATAAGACGCAC
GTTGCAACGTAGTGCTCGCAACTTCCCAATGTCCGATCGGTTGGTACGAAATTGTCCCGA
ATCGAGACCGTCGTGAGGCACAACGATGCAGGACGGAGGGCATGCGTGGGGACGAAACCG
CGCGGCGTGTCGATCGATTGGCCTAGGCATAAGACGCACGCTGCAACGTAGTCCCCGTAA
CTTCCCAACGTCCGATCGGGAGGCGCGAAATCGTTCGGAAACGAGACCGTCGAAAGGCAC
GACGATGCAGGACGGAGGGCATGCGTGGGGACGAAACCGCGCGGCGTGTCGGTCGATTGG
CCTAGGCATAAGACGCACGCTGCAACGCAGCTCTCGCGAATGCACTCCTTCCGATCGGGT
TGCCTAAAAATCGATCGAAGCTCGTGCCTTGCATCCCGCGTTGCCTTTGGATCGTCTCGT
GCTTCGTTCGCACGACAGGAGGACCAAAACGTGGGCATCGAAGCAAGACGTTGCCGTGGG
CGAGTGCGTACTGTGCCCCTCGGGCGCGACGTGCGAGGCAACGCGTTTGGTGCTCGCAAT
TGTGACTGTCATCGCAGTGCGAACTCACGTTTCGACCCCTCGCCAACGCGGGCTTTCCGT
CCGTGTGCGACTGCGAATCTCGATCTTCCTCAAAAATCAGGCCGAAGCTAGATGCTTGCG
GCCTGCGTTGGCTCGGCACCGGATGCTGCTCGTTCGCAAGACGGGAGGACCAGAATGTGG
GCACCGAAAGAAACATTGTCGAGCGTGCGGCGTCCCCCGGGCGCGGCTCGCAGGACATTG
CAGTAAGTGCTCGCGACTGCGACCGTCATCGCACTGCGAACTTCGGCGGCACGATTCGGC
CCCTTGCCAACGCAGGCCTCTCGCTCGTGTGCCCGGAAGTGCCGAATCCGTCTCGGTCCT
TCCCCGATCCATCCCTCGCTTGCCTCCCGTGGGGGGATAAGCAGAACGTCGGCGCCGAAG
AAAACGTTGTTCGAAGCGTGCTGTGCCCCCGGGGCACGACGTGTCGGGCAACGCAGCGGG
CGCTCACGATCGCGACCATCGTCGCACGACAAAATTCGGTCGGTTTCTCGATAATCTCCT
ACCGTGCGTAGCGGGACATGCTCGGGCTCTGCTCGTCGCTTGTTTGCGAACTTTCGGGTT
GGCGGACATGCGGTGCGCTCTGCTTGGGCATCGTGTACGTAAAGCACGTGAGTTGCGATC
GGCGTGTTCGGGTACGTTGGATCCCTGCTCGGGCAGCGACAACGTTCCCCTCACGCCCGT
TCATTGGGTGCTCGAATCTCGCGTTCGGTCATCTCCGTCTCGGTTCCCTGTGCTGCATAC
CCGTTGCGACGGCATCTCCATGCCCAAAAAGACCCCGTTTCGCCCGAAGTCGCGCGTGAC
AACCGCGATCGAAGGCTGAACCGAAAAGCACGCGTCGCGTTCCACCCCGTTCGCAACACC
AGGTGCGGGACTCGGGCCGCGACGTCGCGCTCGAATTCTCGGATGCGGAACACCCAGCGG
GTACGAGGACGCGGATCCTCCGATCACCCCCGAACGAGCGTTCCGCGCTGCGTGCGAACG
ACGTTCTTCCTCGCCCCGGACCTCCGTCCGCGCGATGTCGACGTCGAAGAGGAATGCTAC
CTGGTTGATCCTGCCAGTAGTCATATGCTTGTCTCAAAGATTAAGCCATGCATGTGTAAG

TATGAATTAATTCAGACTGTGAAACTGCGAATGGCTCATTAAATCAGTTATAGTTTGTTT
GATGGTACCTGCTACTCGGATAACCGTAGTAATTCTAGAGCTAATACGTGCAACAAACCC
CGACTTCCGGAAGGGACGCATTTATTAGATAAAAGGTCGACGCGGGCTTCCGCCCGTTGC
TGCGATGATTCATGATAACTCGACGGATCGCACGGCCCTCGTGCCGGCGACGCATCATTC
AAATTTCTGCCCTATCAACTTTCGATGGTAGGATAGTGGCCTACTATGGTGGTGACGGGT
GACGGAGAATTAGGGTTCGATTCCGGAGAGGGAGCCTGAGAAACGGCTACCACATCCAAG
GAAGGCAGCAGGCGCGCAAATTACCCAATCCTGACACGGGGAGGTAGTGACAATAAATAA
CAATACCGGGCTCTCAGAGTCTGGTAATTGGAATGAGTACAATCTAAATCCCTTAACGAG
GATCC
