# Supplementary material for: The Peculiar Landscape of Repetitive Sequences in the Olive (Olea europaea L.) Genome
Source: Genome Biol Evol. 2014 Mar 26;6(4):776–91. doi: 10.1093/gbe/evu058 (PMC4007544; doi:10.1093/gbe/evu058)

Supplementary Material S2. The 5 sets of CLC-BIO parameters used for mapping of Illumina reads to 16 sequences with known redundancy and correlation between known copy number and average coverages (above) and the relation between copy number per haploid genome and the number of Illumina mapped reads for 16 sequences, using the most efficient set of parameters for mapping (Set # 1) (below).

Set	Parameters					Correlation (r^2)	Significance (*P*)
	Mismatch Cost	Deletion Cost	Insertion Cost	Length fraction	Similarity		
1	1	1	1	0.7	0.7	0.7809	0.0036
2	1	1	1	0.8	0.8	0.7794	0.0037
3	1	1	1	0.9	0.9	0.7552	0.0051
4	2	2	2	0.9	0.9	0.7642	0.0045
5	3	3	3	0.9	0.9	0.7484	0.0055

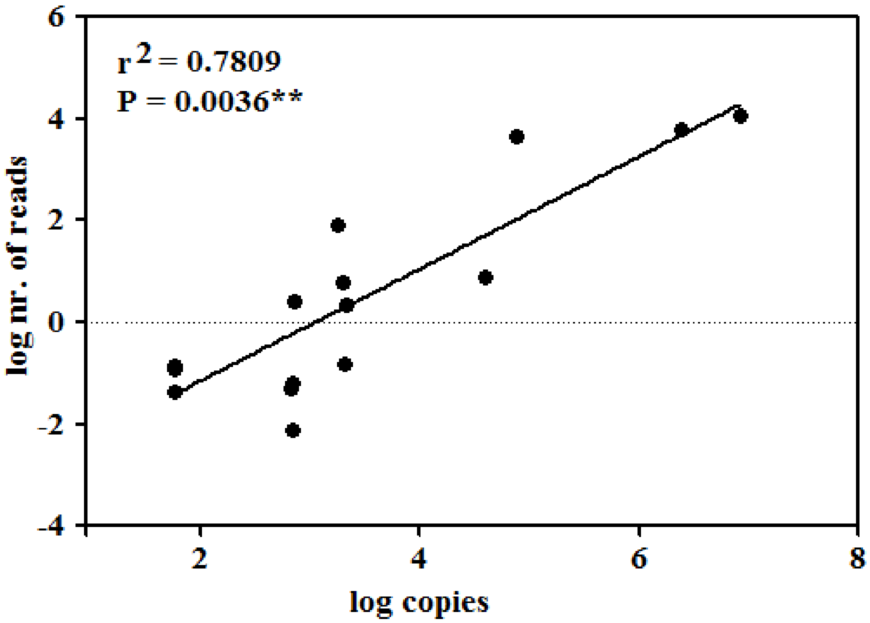

Supplement: Supplementary Data [file supp_evu058_Barghini_et_al_SupplMat_S2.pdf]
