# Supplementary material for: The Peculiar Landscape of Repetitive Sequences in the Olive (Olea europaea L.) Genome
Source: Genome Biol Evol. 2014 Mar 26;6(4):776–91. doi: 10.1093/gbe/evu058 (PMC4007544; doi:10.1093/gbe/evu058)
Supplement: Supplementary Data [file supp_evu058_Barghini_et_al_SupplMat_S3.pdf]

Supplementary materials S3. Determination of the repeat class to which Oe179 and Oe51 belong.

Clusters (obtained submitting a set of 454 or Illumina reads to RepeatExplorer), and indicated as Oe179 and Oe51, were analysed to determine the nature of these repeats. The graphs obtained for both clusters are reported below and their structures clearly indicate that the related sequences are tandemly duplicated in the genome (see Novak et al. 2010).

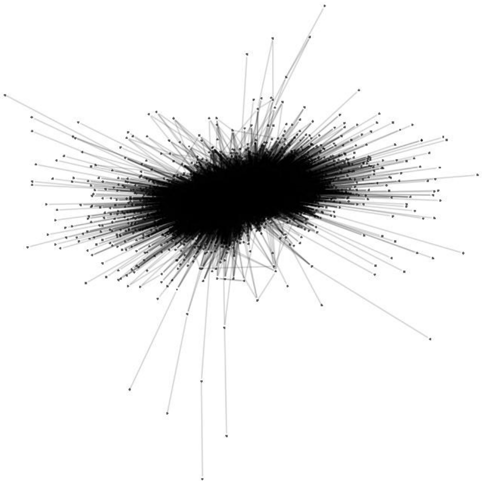

Cluster 4 = Oe179

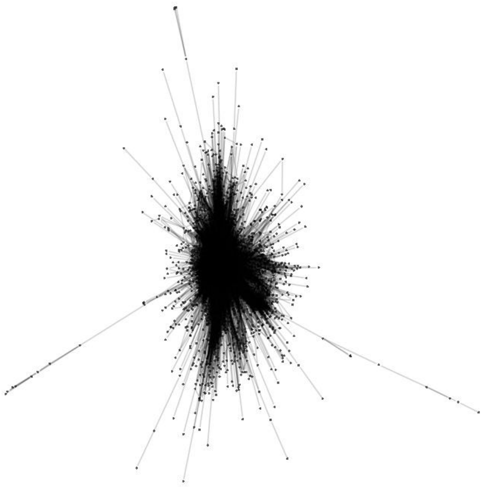

Cluster 13 = Oe51

For each cluster, contigs produced by RepeatExplorer were subjected to a further CAP3 assembly and assembled supercontigs were analysed. Dot-plot analysis showed that these supercontigs contained a number of tandemly arrayed repeats.
The consensus putative repeat unit of both clusters were isolated and subjected to BLAST against a set of 454 or Sanger "real" sequences longer than 400 nt. Dot plots of these sequences clearly confirmed that the repeat units were tandemly arrayed (one sample per cluster is reported below).

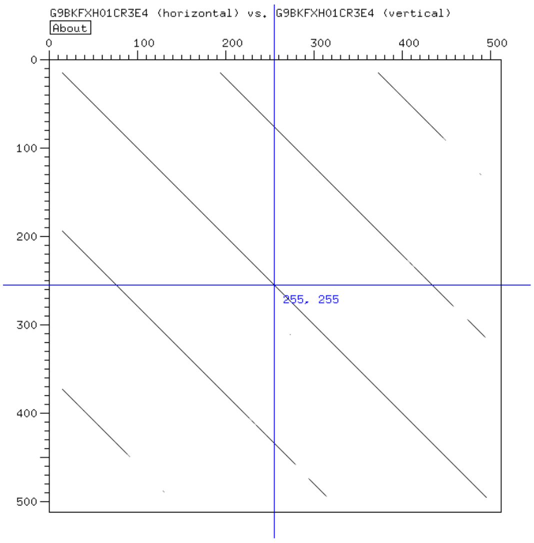

Oe179

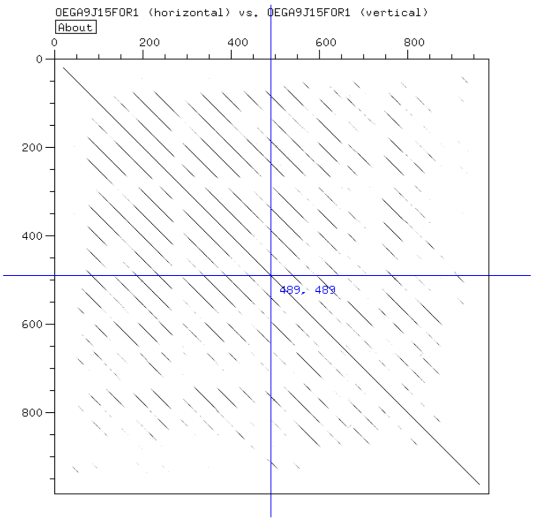

Oe51
