# Supplementary material for: The Peculiar Landscape of Repetitive Sequences in the Olive (Olea europaea L.) Genome
Source: Genome Biol Evol. 2014 Mar 26;6(4):776–91. doi: 10.1093/gbe/evu058 (PMC4007544; doi:10.1093/gbe/evu058)
Supplement: Supplementary Data [file supp_evu058_Barghini_et_al_SupplMat_S4.pdf]

Supplementary materials S4. Alignment of a sample of 20 assembled sequences of the olive whole genome database (numerical codes, below) to Sanger sequences from a small insert library (OEGA codes, above).

```
Query: OEGA1F17REV1
Subject: 152769

OEGA1F17REV1       4 ATTCGTTATATTTATCAATTTCGTCCTCACATTTAAAATTTTGTACTTCA     53
                     |||||||||||||||||||||||||||||||||||||| |||||||||||
152769           644 ATTCGTTATATTTATCAATTTCGTCCTCACATTTAAAA-TTTGTACTTCA    692

OEGA1F17REV1      54 AGATTTTCCTCATTGCTCCCATAACTCTACAATCCTATCTCCACCCCTGC    103
                     ||||||||||||||||||||||||||||||||||||||||||||||||||
152769           693 AGATTTTCCTCATTGCTCCCATAACTCTACAATCCTATCTCCACCCCTGC    742

OEGA1F17REV1     104 TTGGAGTTAACACTGAATGCAAGATTATGCAAAACTATTTACTTCTCTCA    153
                     ||||||||||||||||||||||||||||||||||||||||||||||||||
152769           743 TTGGAGTTAACACTGAATGCAAGATTATGCAAAACTATTTACTTCTCTCA    792

OEGA1F17REV1     154 GGATTTTAAATGATAAATCACTAGAAATATTCGTGAAGAACAATTACAAA    203
                     ||||||||||||||||||||||||||||||||||||||||||||||||||
152769           793 GGATTTTAAATGATAAATCACTAGAAATATTCGTGAAGAACAATTACAAA    842

OEGA1F17REV1     204 TGCTAGAGAACAAAGATTGCAATTGCACCATAAGTTTCTTCCTGGTTTTG    253
                     ||||||||||||||||||||||||||||||||||||||||||||||||||
152769           843 TGCTAGAGAACAAAGATTGCAATTGCACCATAAGTTTCTTCCTGGTTTTG    892

OEGA1F17REV1     254 GTTAGACACTTGTACCGCAGCAAAGATTAATTGTAACCAATTCAATTTTT    303
                     ||||||||||||||||||||||||||||||||||||||||||||||||||
152769           893 GTTAGACACTTGTACCGCAGCAAAGATTAATTGTAACCAATTCAATTTTT    942

OEGA1F17REV1     304 GAATTGTCTGCACCTATGCATAGCTTGAATAAGGGCATCAATCCTCTATA    353
                     ||||||||||||||||||||||||||||||||||||||||||||||||||
152769           943 GAATTGTCTGCACCTATGCATAGCTTGAATAAGGGCATCAATCCTCTATA    992

OEGA1F17REV1     354 GTGTCTCTGCATTTTTACTAGTAATGGTGAGAGTGAGCACTAGCAACACT    403
                     ||||||||||||||||||||||||||||||||||||||||||||||||||
152769           993 GTGTCTCTGCATTTTTACTAGTAATGGTGAGAGTGAGCACTAGCAACACT   1042

OEGA1F17REV1     404 ATTGTCTGTTTAACTGAGAGGTTAACAAATTTTCCCAGACAAAAATAACC    453
                     ||||||||||||||||||||||||||||||||||||||||||||||||||
152769          1043 ATTGTCTGTTTAACTGAGAGGTTAACAAATTTTCCCAGACAAAAATAACC   1092

OEGA1F17REV1     454 TTGTACGATAACTATTTATCATCCTTCACTCCAATAACCAATATTTTATG    503
                     ||||||||||||||||||||||||||||||||||||||||||||||||||
152769          1093 TTGTACGATAACTATTTATCATCCTTCACTCCAATAACCAATATTTTATG   1142

OEGA1F17REV1     504 TTGTACGACAATCATAATTATCATGATGTTTTTA-TTGACTACTGGTTGG    552
                     |||||||||||||||||||||||||||||||||| |||||||||||||||
152769          1143 TTGTACGACAATCATAATTATCATGATGTTTTTATTTGACTACTGGTTGG   1192

OEGA1F17REV1     553 AATTTTGACTTTGATCACTTTGTCACGGTAGGAAACATGCAGATTTTAAT    602
                     ||||||||||||||||||||||||||||||||||||||||||||||||||
152769          1193 AATTTTGACTTTGATCACTTTGTCACGGTAGGAAACATGCAGATTTTAAT   1242

OEGA1F17REV1     603 AGTGAATGTCTAAAAATTCAACAAAGCTCATGGAGTAAAGCTTGGTTTCT    652
                     ||||||||||||||||||||||||||||||||||||||||||||||||||
152769          1243 AGTGAATGTCTAAAAATTCAACAAAGCTCATGGAGTAAAGCTTGGTTTCT   1292
```

```
OEGA1F17REV1      653 GCATTGATTCCTGAACAT-AAAAAACAGAAGACTTTAAGCCTTGCATGTT     701
                      |||||||||||||||||| |||||||||||||||||||||||||||||||
152769           1293 GCATTGATTCCTGAACATAAAAAAACAGAAGACTTTAAGCCTTGCATGTT    1342

OEGA1F17REV1      702 CGAGTACATTCACGACAAACTCTCCTGTTTAAATACACATTCATGTTCTT     751
                      ||||||||||||.|||||||||||||||||||||||||||||||||||||
152769           1343 CGAGTACATTCAGGACAAACTCTCCTGTTTAAATACACATTCATGTTCTT    1392

OEGA1F17REV1      752 AAGTTCATATAAATATCACAAGTATAAAGGAGACACTAAGACCACTTTGT     801
                      ||||||||||||||||||||||||||||||||||||||||||||||||||
152769           1393 AAGTTCATATAAATATCACAAGTATAAAGGAGACACTAAGACCACTTTGT    1442

OEGA1F17REV1      802 ACACAGAACAAAAATCGACTATCCGATCCACCATGT     837
                      ||||||||||||||||||||||||||||||||||||
152769           1443 ACACAGAACAAAAATCGACTATCCGATCCACCATGT    1478
```

Query: OEGA1O09FOR1
Subject: 83744

```
OEGA1O09FOR1        2 GAGCTTGTATCAACACTGATATGGCAAAATATAATCGGACTTTTTTTATT      51
                      ||||||||||||||||||||||||||||||||||||||||||||||||||
83744              60 GAGCTTGTATCAACACTGATATGGCAAAATATAATCGGACTTTTTTTATT     109

OEGA1O09FOR1       52 TCTTTTTTTCCATCTTCCAATGATATCGCGTCATATGTTGTATGTAAGAT     101
                      || |||||||||||||||||||||||||||||||||||||||||||||||
83744             110 TC-TTTTTTCCATCTTCCAATGATATCGCGTCATATGTTGTATGTAAGAT     158

OEGA1O09FOR1      102 TTTTCTATAAAAAAATTCTGTACCTATGACATTGTTCTAATCCTATACAA     151
                      ||||||||||||||||||||||||||||||||||||||||||||||||||
83744             159 TTTTCTATAAAAAAATTCTGTACCTATGACATTGTTCTAATCCTATACAA     208

OEGA1O09FOR1      152 TGCTATATAATCCAGAATTAACAATCACTAAAGCCTATATTCCACTCAGC     201
                      ||||||||||||||||||||||||||||||||||||||||||||||||||
83744             209 TGCTATATAATCCAGAATTAACAATCACTAAAGCCTATATTCCACTCAGC     258

OEGA1O09FOR1      202 TTAGTAGATCCAACAGCTATAAATTGACAATTAGTAGATCCTATTACAAC     251
                      ||||||||||||||||||||||||||||||||||||||||||||||||||
83744             259 TTAGTAGATCCAACAGCTATAAATTGACAATTAGTAGATCCTATTACAAC     308

OEGA1O09FOR1      252 TCTACGACATTATGTTTATTTTGAAATTTGAAGTTTGATTAGGACTAACC     301
                      ||||||||||||||||||||||||||||||||||||||||||||||||||
83744             309 TCTACGACATTATGTTTATTTTGAAATTTGAAGTTTGATTAGGACTAACC     358

OEGA1O09FOR1      302 ATTTTGTACGGATGTAGCTAGGGTTTTTGTAGAAATGTTTTTAGGGTTAT     351
                      ||||||||||||||||||||||||||||||||||||||||||||||||||
83744             359 ATTTTGTACGGATGTAGCTAGGGTTTTTGTAGAAATGTTTTTAGGGTTAT     408

OEGA1O09FOR1      352 TATGAATGTACAGTTCTTACAACCTCCTCACATGAAGGGACCGTCATGCA     401
                      ||||||||||||||||||||||||||||||||||||||||||||||||||
83744             409 TATGAATGTACAGTTCTTACAACCTCCTCACATGAAGGGACCGTCATGCA     458

OEGA1O09FOR1      402 TGCTGTACACAAAGTGTATAGCAAGCATGATTCACGTTTTTATCATATTC     451
                      |||||||||||||||||||||||||||||||||||.||||||||||||||
83744             459 TGCTGTACACAAAGTGTATAGCAAGCATGATTCACNTTTTTATCATATTC     508

OEGA1O09FOR1      452 AGTTTTGCTTCTTGTACGAAGGAAATCTTGCCAAATGTTTTACAGGTGCC     501
                      |.|||||||.||||||||||||||||||||||||||||||||||||||||
83744             509 ANTTTTGCTNCTTGTACGAAGGAAATCTTGCCAAATGTTTTACAGGTGCC     558

OEGA1O09FOR1      502 GATTGGTTACTCTTTTAACCTTTGACGAATGAAAAATTTCCAATTGTATC     551
                      ||||||||||||||||||||||||||||||||||||||||||||||||||
83744             559 GATTGGTTACTCTTTTAACCTTTGACGAATGAAAAATTTCCAATTGTATC     608
```

```
OEGA1O09FOR1      552 TATAAATAATCTAATAGTACTTTATTCTGTACATGTAGTACTGCCCTATC    601
                      ||||||||||||||||||||||||||||||||||||||||||||||||||
83744             609 TATAAATAATCTAATAGTACTTTATTCTGTACATGTAGTACTGCCCTATC    658

OEGA1O09FOR1      602 ATGTTTTGGTCAAACACAAGTTTGTGATCTAATTTATACTTATCGCAGCC    651
                      ||||||||||||||||||||||||||||||||||||||||||||||||||
83744             659 ATGTTTTGGTCAAACACAAGTTTGTGATCTAATTTATACTTATCGCAGCC    708

OEGA1O09FOR1      652 TTCAGTACATAATTCAAAGTGCACGACAAAATGTGATGTATATGTCATGT    701
                      ||||||||||||||||||||||||||||||||||||||||||||||||||
83744             709 TTCAGTACATAATTCAAAGTGCACGACAAAATGTGATGTATATGTCATGT    758

OEGA1O09FOR1      702 CATATCAAATCATTTTTGCACGCCAGACTAGGAGACCAATTTAGCAGCCC    751
                      ||||||||||||||||||||||||||||||||||||||| |||||||||.
83744             759 CATATCAAATCATTTTTGCACGCCAGACTAGGAGACCAA-TTAGCAGCCA    807

OEGA1O09FOR1      752 ATATTTTTGCACTTCTGAAAAACATTCCACAACTTGATTGGATCTTAGAA    801
                      ||||||||||||||||||||||||||||||||||||||||||||||||||
83744             808 ATATTTTTGCACTTCTGAAAAACATTCCACAACTTGATTGGATCTTAGAA    857

OEGA1O09FOR1      802 ACTTGTTT-CACTTTCCACCCAAAAAAACAAAGATTTTTCTCAAATAAAG    850
                      || ||||| |||||||||.||.|||||||||||| |.|||||||||||||
83744             858 AC-TGTTTCCACTTTCCAACCCAAAAAACAAAGA-TATTCTCAAATAAAG    905

OEGA1O09FOR1      851 AGTGATT-CACT-ACTTGTTTAACTTTAAATGATGATG-AAAGATGTGAG    897
                      ||||||| |||| |||||||||||||.||||||||||| |||||||||||
83744             906 AGTGATTCCACTAACTTGTTTAACTTAAAATGATGATGAAAAGATGTGAG    955

OEGA1O09FOR1      898 ATTGTTG    904
                      |||||||
83744             956 ATTGTTG    962


Query: OEGA1P06REV1
Subject: 17720

OEGA1P06REV1        1 GTTGTCTGCATGTGATTTCAATTTATATTTTACGTTTGTGCTTGCTGGTT     50
                      ||||||||.|||||||||||||||||||||||||||||||||||||||||
17720              24 GTTGTCTGAATGTGATTTCAATTTATATTTTACGTTTGTGCTTGCTGGTT     73

OEGA1P06REV1       51 ATACTAGGAGCAGACAAAATGTATGAATGCTAGCTCATGCTATATACAGT    100
                      |||||||||||||||||||||||||||||.||||||||||||||||||||
17720              74 ATACTAGGAGCAGACAAAATGTATGAATGNTAGCTCATGCTATATACAGT    123

OEGA1P06REV1      101 CCAAAAATAAATTTTTCATAGTTGGCATCAGGAAATATTACTTGGTGGAC    150
                      ||||||||||||||||||.||.||||||||||||||||||||||||||||
17720             124 CCAAAAATAAATTTTTCANAGNTGGCATCAGGAAATATTACTTGGTGGAC    173

OEGA1P06REV1      151 TCCTATTTTTCTCATCGACCAATATATATGGCCTCGTACAAAGGTTTAGA    200
                      |||||||||||||||||||||||||||||||||.||||||||||||||||
17720             174 TCCTATTTTTCTCATCGACCAATATATATGGCCNCGTACAAAGGTTTAGA    223

OEGA1P06REV1      201 CGTACTGTATCACTTCCAACTGCTTTTTGACAAACAAATAGGTCGGCGCC    250
                      ||||||.||||||||||||||.|||.||||||||||||||||||||||||
17720             224 CGTACTNTATCACTTCCAACTGNTTTNTGACAAACAAATAGGTCGGCGCC    273

OEGA1P06REV1      251 ATAGATTTAGGAATGCTCTTGAGAGGTTCAACTTCAAAGACTCTTTGTGC    300
                      ||||||||||||||||||||||||||||||||||||||.|||||||||||
17720             274 ATAGATTTAGGAATGCTCTTGAGAGGTTCAACTTCAAANACTCTTTGTGC    323

OEGA1P06REV1      301 AGGAACATGATCGAGCGTGCATTTAGTGTGTTGAAGTAGAGATGGAAAAT    350
                      ||||||||||||||||||||||||||||||||||||||||||||||||||
17720             324 AGGAACATGATCGAGCGTGCATTTAGTGTGTTGAAGTAGAGATGGAAAAT    373
```

```
OEGA1P06REV1      351 CTTGAATCGTATGCCCAGCCACTCTTTTCCAACACAAGTTGCAGTTGTCA    400
                      ||||||||||||||||||||||||||||||||||||||||||||||||||
17720             374 CTTGAATCGTATGCCCAGCCACTCTTTTCCAACACAAGTTGCAGTTGTCA    423

OEGA1P06REV1      401 TAGT---GGGTATTTATAATTTTTGCATCATTCTGGATTTGTTGACAAGA    447
                      ||||   |||||||||||||||||||||||||||||||||||||||||||
17720             424 TAGTGGGGGGTATTTATAATTTTTGCATCATTCTGGATTTGTTGACAAGA    473

OEGA1P06REV1      448 AGTTCGCAAGAGCAGAAATAGATGACAAGGTAGCCAAAGCGGAGCTACCT    497
                      ||||||||||||||||||| ||||||||||||||||||||||||||||||
17720             474 AGTTCGCAAGAGCAGAAAT-GATGACAAGGTAGCCAAAGCGGAGCTACCT    522

OEGA1P06REV1      498 ACTGTTGAGGATGAAATTTAGGCCGAAATGAATGCGTCGGACATACAACA    547
                      ||||||||||||||||||||||||||||||||||||||||||||||||||
17720             523 ACTGTTGAGGATGAAATTTAGGCCGAAATGAATGCGTCGGACATACAACA    572

OEGA1P06REV1      548 AAGTGAGTGGGATAGGTTTCGCGATTACATGGCACAACAACCATGATCAT    597
                      ||||||||||||||||||||||||||||||||||||||||||||||||||
17720             573 AAGTGAGTGGGATAGGTTTCGCGATTACATGGCACAACAACCATGATCAT    622

OEGA1P06REV1      598 CGCTTCTAGGAACCTCGCATCCAGTACCTATGTATTGGTATGAAGGAACT    647
                      ||||||||||||||||||||||||||||.|||||||||||||||||||||
17720             623 CGCTTCTAGGAACCTCGCATCCAGTACCTNTGTATTGGTATGAAGGAACT    672

OEGA1P06REV1      648 TTGCAAGTTTGAGTCAATGTATTTGTACACGTTGCGGTAGCTTTGTAAGT    697
                      .||||||||||||||||||||||||||||||||||||||||||||||.||
17720             673 NTGCAAGTTTGAGTCAATGTATTTGTACACGTTGCGGTAGCTTTGTAGGT    722

OEGA1P06REV1      698 GTGTTTTGATTATGTACGTCCGTGTAACATGAAATAATTGATTATGAAAT    747
                      ||||||||||||||||||||||||||||||||||||||||||||||||||
17720             723 GTGTTTTGATTATGTACGTCCGTGTAACATGAAATAATTGATTATGAAAT    772

OEGA1P06REV1      748 TTGCAGCATGAATTGGGACTTTGGGGACCGACGACGCAGTGACTATTGTA    797
                      ||||||||||||||||||||||||||||||.|||||||||||||||||||
17720             773 TTGCAGCATGAATTGGGACTTTGGGGACCGANGACGCAGTGACTATTGTA    822

OEGA1P06REV1      798 TCATTTTTTTCCCAGTTCGAAGTCTTT    824
                      |||||||||||||||||||||||||||
17720             823 TCATTTTTTTCCCAGTTCGAAGTCTTT    849


Query: OEGA2C17FOR1
Subject: 5818

OEGA2C17FOR1        1 ATTATTGATTTGGACATCATCGATTATGTAACAATTAATTCTTTTGGTAC     50
                      ||||||||||||||||||||||||||||||||||||||||||||||||||
5818               71 ATTATTGATTTGGACATCATCGATTATGTAACAATTAATTCTTTTGGTAC    120

OEGA2C17FOR1       51 TTGCTGAAGATTGTATTTGAAAATGCAATTCTCTTGGATCTTGGATTATA    100
                      ||||||||||||||||||||||||||||||||||||||||||||||||||
5818              121 TTGCTGAAGATTGTATTTGAAAATGCAATTCTCTTGGATCTTGGATTATA    170

OEGA2C17FOR1      101 AATCCTCATTTATCTTAGGACTATTTTGGAATTCTTACCGTGAGATTAAT    150
                      ||||||||||||||||||||||||||||||||||||||||||||||||||
5818              171 AATCCTCATTTATCTTAGGACTATTTTGGAATTCTTACCGTGAGATTAAT    220

OEGA2C17FOR1      151 ACAAGTTTTGTGTTATTCATATGTGCTAATCAAGATTTAGCACGAGCTGT    200
                      ||||||||||||||||||||||||||||||||||||||||||||||||||
5818              221 ACAAGTTTTGTGTTATTCATATGTGCTAATCAAGATTTAGCACGAGCTGT    270

OEGA2C17FOR1      201 GTGACAAAATTCAAATCAGAGTAGAAGTTAGAGGTGCCAAGATGAGTTGA    250
                      ||||||||||||||||||||||||||||||||||||||||||||||||||
5818              271 GTGACAAAATTCAAATCAGAGTAGAAGTTAGAGGTGCCAAGATGAGTTGA    320
```

```
OEGA2C17FOR1     251 ACAGTCTGTGACCTGAGTTCTAACCCTTTAAATAGTTGATAAAATGTGTT    300
                     ||||||||||||||||||||||||||||||||||||||||||||||||.|
5818             321 ACAGTCTGTGACCTGAGTTCTAACCCTTTAAATAGTTGATAAAATGTGNT    370

OEGA2C17FOR1     301 ACTGAATAATTGATTA-AAATTGTAGATTCAATGAAATGTGTGAAAAAAT    349
                     |||||||||||||||| |||||||||||||||||||||||||||||||||
5818             371 ACTGAATAATTGATTAGAAATTGTAGATTCAATGAAATGTGTGAAAAAAT    420

OEGA2C17FOR1     350 GCATTTTTAGAATTGAGGACCAAACAGAAGTTTCAATTTCCATTTCAGAT    399
                     ||||||||||||||||||||||||||||||||||||||||||||||||||
5818             421 GCATTTTTAGAATTGAGGACCAAACAGAAGTTTCAATTTCCATTTCAGAT    470

OEGA2C17FOR1     400 ACATGAGTTAATTGTCTCAATGTTTACTGAACTTGCATCTGTATTCAATT    449
                     ||||||||||||||||||||||||||||||||||||||||||||||||||
5818             471 ACATGAGTTAATTGTCTCAATGTTTACTGAACTTGCATCTGTATTCAATT    520

OEGA2C17FOR1     450 ATTCATTGAAGTTTATGACGTCAAGTTTTAGCCACAAAATTTATCAAAAC    499
                     ||||||||||||||||||||||||||||||||||||||||||||||||||
5818             521 ATTCATTGAAGTTTATGACGTCAAGTTTTAGCCACAAAATTTATCAAAAC    570

OEGA2C17FOR1     500 ATTACTTACCGACATAACAGCCATTTCAAATCGATTTAGTGACGTGACTA    549
                     ||||||||||||||||||||||||||||||||||||||||||||||||||
5818             571 ATTACTTACCGACATAACAGCCATTTCAAATCGATTTAGTGACGTGACTA    620

OEGA2C17FOR1     550 ACGGAATGCACATGTGGCGTCCCATCACGCAATTCACAAATTGTCACATA    599
                     ||||||||||||||||||||||||||||||||||||||||||||||||||
5818             621 ACGGAATGCACATGTGGCGTCCCATCACGCAATTCACAAATTGTCACATA    670

OEGA2C17FOR1     600 TTCAATTGCTCATTAAATTTTGAAAAACATCTTCGATTGCTCATTGAACT    649
                     ||||||||||||||||||||||||||||||||||||||||||||||||||
5818             671 TTCAATTGCTCATTAAATTTTGAAAAACATCTTCGATTGCTCATTGAACT    720

OEGA2C17FOR1     650 TTAAGACGGTCAAATTTTGACCATTGAAAATTTAAAGCATCTTTTGTTGC    699
                     ||||||||||||||||||||||||||||||||||||||||||||||||||
5818             721 TTAAGACGGTCAAATTTTGACCATTGAAAATTTAAAGCATCTTTTGTTGC    770

OEGA2C17FOR1     700 TCACCAAACTTTGAAAACGTCTTTGATTGTTTACTGAACTTTTAAATGAT    749
                     ||||||||||||||||||||||||||||||||||||||||||||||||||
5818             771 TCACCAAACTTTGAAAACGTCTTTGATTGTTTACTGAACTTTTAAATGAT    820

OEGA2C17FOR1     750 CGAACTTTGGTTAATAAACTTATA    773
                     ||||||||||||||||||||||||
5818             821 CGAACTTTGGTTAATAAACTTATA    844


Query: OEGA2E16FOR1
Subject: 105150

OEGA2E16FOR1       1 AAAAAATAATAATATAGAAATCCAAGAATATGAGGGAATAGAAGGTTATG     50
                     |.|||||||||||||||||||||||||||||||||||||||||||.||||
105150           539 AGAAAATAATAATATAGAAATCCAAGAATATGAGGGAATAGAAGGCTATG    588

OEGA2E16FOR1      51 GATTAAAGGTAACTTTTGATAATAATAAATTTAAGAAAATGCAAAAGCTA    100
                     |||||||.||||||||||||||||||||||||||||||||.||||||.||
105150           589 GATTAAAAGTAACTTTTGATAATAATAAATTTAAGAAAATACAAAAGTTA    638

OEGA2E16FOR1     101 TCCTTGGATTTGAAAATAGGAAAATGGGAAATTCTAGATAGAATATGGAG    150
                     |||||.|||||||||||||||||||||||||||.||||||||||||||||
105150           639 TCCTTAGATTTGAAAATAGGAAAATGGGAAATTTTAGATAGAATATGGAG    688

OEGA2E16FOR1     151 AAGAGAAAGAATAGCAATAAATGAATATCATAAGATTGAAAAAACTATAG    200
                     |||||||||||||||||||||||||||.||||||||||||||||||||||
105150           689 AAGAGAAAGAATAGCAATAAATGAATACCATAAGATTGAAAAAACTATAG    738
```

```
OEGA2E16FOR1     201 ATGTTAGAACCACTCAAGGAACACATAAAATAAATCTTTTAACAAAAAGT  250
                     ||||||||||.|||||||||||||||||||||||||||.|||||||||||
105150           739 ATGTTAGAACAACTCAAGGAACACATAAAATAAATCTTCTAACAAAAAGT  788

OEGA2E16FOR1     251 ACAGTATCAGAATTATTATCAAAAATAAGTAAAAAGCATAGTGAAAAGAT  300
                     ||||||||||||||||||||||||||||||||||||||||||||||||||
105150           789 ACAGTATCAGAATTATTATCAAAAATAAGTAAAAAGCATAGTGAAAAGAT  838

OEGA2E16FOR1     301 CCAATATGTTTATTTAGCAGGAATACAAATAATGTTTAAATCATTATTTA  350
                     ||||||||||||||||||||||||||||||||||||||||||||||||||
105150           839 CCAATATGTTTATTTAGCAGGAATACAAATAATGTTTAAATCATTATTTA  888

OEGA2E16FOR1     351 GAGAGGGAATAGATTCACCATTAACCGTAAGCTTACATGATGGAAGAATT  400
                     |||||||||||||||||||||||||.|||||.||||||||||||||||||
105150           889 GAGAGGGAATAGATTCACCATTAACTGTAAGTTTACATGATGGAAGAATT  938

OEGA2E16FOR1     401 ATAAATCTTGAAGATGGTCATTTAGGAACTATAGAAGGAAATTTAGCATA  450
                     ||||||||||||||||||||||||||||||||||||||||||||||||||
105150           939 ATAAATCTTGAAGATGGTCATTTAGGAACTATAGAAGGAAATTTAGCATA  988

OEGA2E16FOR1     451 CCAAAAGCTAATATTTACTTGTTACCCAAAATATTGTGTCTCATTGAAAG  500
                     .||||||.|||||||||||||||||||||||||||||||||||||.||||
105150           989 TCAAAAGTTAATATTTACTTGTTACCCAAAATATTGTGTCTCATTAAAAG 1038

OEGA2E16FOR1     501 ATAAAAACTTTAATGAAGTCTTAGGATTACATTTTAAATTACATAGAAAT  550
                     |||||||.||||||||||||||||||||||||||.|||||||||||.|||
105150          1039 ATAAAAATTTTAATGAAGTCTTAGGATTACATTTCAAATTACATAGGAAT 1088

OEGA2E16FOR1     551 GATATAATGGAACCAGGATACCAAATTATGACTATGGAATATAGGGCATT  600
                     ||||||||||||||||||.|.|||||||||||||||||||||||||||||
105150          1089 GATATAATGGAACCAGGAGATCAAATTATGACTATGGAATATAGGGCATT 1138

OEGA2E16FOR1     601 ATATACATTTACTAATTCTAATTATGAAAATATTTATTCTAATAAACCAT  650
                     ||||||||||||||||||||||||||..|||||||||||.||||||||||
105150          1139 ATATACATTTACTAATTCTAATTATGGGAATATTTATTCCAATAAACCAT 1188

OEGA2E16FOR1     651 ATATAGAAATTCATAAAGAATGTAAGGATATTAGTAAGATTGTGGAACCA  700
                     |.||||||||||||||.||||||||||||||||||||.||.|||.|||||
105150          1189 ACATAGAAATTCATAAGGAATGTAAGGATATTAGTAAAATAGTGNAACCA 1238

OEGA2E16FOR1     701 AACAAACAAATAAACCTTATCCCAGAAG-ATATGAATTTAATATAACTAA  749
                     ||||||||||||||.||||||||||||| |||||||||||||||||||||
105150          1239 AACAAACAAATAAATCTTATCCCAGAAGAATATGAATTTAATATAACTAA 1288

OEGA2E16FOR1     750 AAATAAGCTTGAAATAACAGAG   771
                     ||||||||.|||||||||||||
105150          1289 AAATAAGCCTGAAATAACAGAG  1310
```

Query: OEGA2I17REV1
Subject: 20023

```
OEGA2I17REV1       1 ATTCAGTTGTTATTTGTGAGTTTTTTAGATTACTTCATCGACTATTGTGT   50
                     ||||||||||||||||||||||||||||||||||||||||||||||||||
20023            829 ATTCAGTTGTTATTTGTGAGTTTTTTAGATTACTTCATCGACTATTGTGT  878

OEGA2I17REV1      51 TTATGTTGGCGCTCGTGGTGTATGAGCATTGCTTGTTCCATTGCAGCATA  100
                     ||||||||||||||||||||||||||||||||||||||||||||||||||
20023            879 TTATGTTGGCGCTCGTGGTGTATGAGCATTGCTTGTTCCATTGCAGCATA  928

OEGA2I17REV1     101 TTTATTGTTTATTGGCCCATACTTTGACCTTTAGCTTCTATTTATAGTGA  150
                     ||||||||||||||||||||||||||||||||||||||||||||||||||
20023            929 TTTATTGTTTATTGGCCCATACTTTGACCTTTAGCTTCTATTTATAGTGA  978
```

```
OEGA2I17REV1      151 TTTGCCAACTGTTAGCAATATATGGAATTTGATATACATGTTGAGGTTGA    200
                      ||||||||||||||||||||||||||||||||||||||||||||||||||
20023             979 TTTGCCAACTGTTAGCAATATATGGAATTTGATATACATGTTGAGGTTGA   1028

OEGA2I17REV1      201 GCTGCTGATGTTAGCAAGATGGGCCTTCTTGGCCTCTGAATAATGTATTT    250
                      ||||||||||||||||||||||||||||||||||||||||||||||||||
20023            1029 GCTGCTGATGTTAGCAAGATGGGCCTTCTTGGCCTCTGAATAATGTATTT   1078

OEGA2I17REV1      251 TTCATCCGCATGTTACTGGGAATGATTCATTACAATGAGCTGAATGGTTC    300
                      ||||||||||||||||||||||||||||||||||||||||||||||||||
20023            1079 TTCATCCGCATGTTACTGGGAATGATTCATTACAATGAGCTGAATGGTTC   1128

OEGA2I17REV1      301 AAATGATAATAAGAATTACCCATTTAGGAAGTGCGTTATGATAAATCTTT    350
                      ||||||||||||||||||||||||||||||||||||||||||||||||||
20023            1129 AAATGATAATAAGAATTACCCATTTAGGAAGTGCGTTATGATAAATCTTT   1178

OEGA2I17REV1      351 GATGCTTCTGGTTTTTCTTTTCTATGATTCGTGAATATTGCTGCTTGTTT    400
                      ||||||||||||||||||||||||||||||||||||||||||||||||||
20023            1179 GATGCTTCTGGTTTTTCTTTTCTATGATTCGTGAATATTGCTGCTTGTTT   1228

OEGA2I17REV1      401 GTGAGCTGACTGCATTAAAATTGAAAGTGTATCTTATCCATGAATTTGAA    450
                      ||||||||||||||||||||||||||||||||||||||||||||||||||
20023            1229 GTGAGCTGACTGCATTAAAATTGAAAGTGTATCTTATCCATGAATTTGAA   1278

OEGA2I17REV1      451 GTTGAAGCTACGCCGAAGCCTTTTTAACATAAAGGTGGTGAGGGAGAGGG    500
                      ||||||||||||||||||||||||||||||||||||||||||||||||||
20023            1279 GTTGAAGCTACGCCGAAGCCTTTTTAACATAAAGGTGGTGAGGGAGAGGG   1328

OEGA2I17REV1      501 TTGGAGTTGGTGATAATGAGGAAAAAGAAATGGAGTGAACCAGATTGCAC    550
                      ||||||||||||||||||||||||||||||||||||||||||||||||||
20023            1329 TTGGAGTTGGTGATAATGAGGAAAAAGAAATGGAGTGAACCAGATTGCAC   1378

OEGA2I17REV1      551 TGCTTGGGGGTTGATTGTTCAAGCTAATGAGTTTAGTCACACTGATGGTT    600
                      ||||||||||||||||||||||||||||||||||||||||||||||||||
20023            1379 TGCTTGGGGGTTGATTGTTCAAGCTAATGAGTTTAGTCACACTGATGGTT   1428

OEGA2I17REV1      601 AAAATTCTCTTATGGGCAGCTCATGTTCATGACCCTAATTAAAATTCTCT    650
                      ||||||||||||||||||||||||||||||||||||||||||||||||||
20023            1429 AAAATTCTCTTATGGGCAGCTCATGTTCATGACCCTAATTAAAATTCTCT   1478

OEGA2I17REV1      651 TATGGGCAGCTCTATTTATTATTTCATTTTGAATAACTGCTGCTTCTCTT    700
                      ||||||||||||||||||||||||||||||||||||||||||||||||||
20023            1479 TATGGGCAGCTCTATTTATTATTTCATTTTGAATAACTGCTGCTTCTCTT   1528

OEGA2I17REV1      701 CTCTAGAATGTCC-TTTGTTTCCTGTCATTGTCTCCATGTGCTCATTCAC    749
                      ||||||||||||| ||||||||||||||||||||||||||||||||||||
20023            1529 CTCTAGAATGTCCTTTTGTTTCCTGTCATTGTCTCCATGTGCTCATTCAC   1578

OEGA2I17REV1      750 ATGGACTTAACTTACGGTAAATTTGGGAAATTGCAGATAGCAGCCCAAAG    799
                      |||||||||||||||||||||||||||||||||||||||||||.||||||
20023            1579 ATGGACTTAACTTACGGTAAATTTGGGAAATTGCAGATAGCAGGCCAAAG   1628

OEGA2I17REV1      800 CTGAT-ACA-GATCAGCTGATGGATACTCCGGATATGTGGATGGTAAATT    847
                      ||||| ||| ||||||||||||||||||||||||||||||||||||||||
20023            1629 CTGATAACAGGATCAGCTGATGGATACTCCGGATATGTGGATGGTAAATT   1678

OEGA2I17REV1      848 G-GAGA    852
                      | ||||
20023            1679 GAGAGA   1684
```

```
Query: OEGA2K12FOR1
Subject: 92871


OEGA2K12FOR1        2 TTTGATTAGTTGAACTTTCTCTAGTTTTCTTATAAATTTTTCTCTTGTGG     51
                      ||||||||||||||||||||||||||||||||||||||||||||||||||
92871             393 TTTGATTAGTTGAACTTTCTCTAGTTTTCTTATAAATTTTTCTCTTGTGG    442

OEGA2K12FOR1       52 TGTCACTAATAATTTCTTTTCATTTATTTAGTCTCATGATACGTACATTA    101
                      ||||||||||||||||||||||||||||||||.|||||||||||||||||
92871             443 TGTCACTAATAATTTCTTTTCATTTATTTAGTNTCATGATACGTACATTA    492

OEGA2K12FOR1      102 ACTTTATATCTAGTTTTATTCACATGTTTGTATCTTATTATTGCTTTGGT    151
                      ||||||||||||||||||||||||||||||||||||||||||||||||||
92871             493 ACTTTATATCTAGTTTTATTCACATGTTTGTATCTTATTATTGCTTTGGT    542

OEGA2K12FOR1      152 ATAACTTAATTGCATGTTTTCATTTTTGCTTCTAATTTAGCAACTCTTAT    201
                      ||||||||||||||||||||||||||||||||||||||||||||||||||
92871             543 ATAACTTAATTGCATGTTTTCATTTTTGCTTCTAATTTAGCAACTCTTAT    592

OEGA2K12FOR1      202 GTTAAATTTTGATGTGTATTTGAACTTGTACATGGTGTTGAAGGTACCGA    251
                      ||||||||||||||||||||||||||||||||||||||||||||||||||
92871             593 GTTAAATTTTGATGTGTATTTGAACTTGTACATGGTGTTGAAGGTACCGA    642

OEGA2K12FOR1      252 TTTTACAATTTAGGCTACATGATCATTTTACACTACACTCTTTTCCTTAA    301
                      ||||||||||||||||||||||||||||||||||||||||||||||||||
92871             643 TTTTACAATTTAGGCTACATGATCATTTTACACTACACTCTTTTCCTTAA    692

OEGA2K12FOR1      302 ATAGATTCTTCTTCAAAATCTGTTCAAATTGTTCTTAATGATGCTTATTC    351
                      ||||||||||||||||||||||||||||||||||||||||||||||||||
92871             693 ATAGATTCTTCTTCAAAATCTGTTCAAATTGTTCTTAATGATGCTTATTC    742

OEGA2K12FOR1      352 AATCCCTGAATTGTGATTCTACTCTCAAATTTTATTTATAGATATGAATC    401
                      ||||||||||||||||||||||||||||||||||||||||||||||||||
92871             743 AATCCCTGAATTGTGATTCTACTCTCAAATTTTATTTATAGATATGAATC    792

OEGA2K12FOR1      402 CAGATGTGACAACAGCAGTTTCTATATTATCTCATGTTTGGACATCTACA    451
                      ||||||||||||||||||||||||||||||||||||||||||||||||||
92871             793 CAGATGTGACAACAGCAGTTTCTATATTATCTCATGTTTGGACATCTACA    842

OEGA2K12FOR1      452 TCATTATGGGTTTTGTTAATGCTACTTCTTCGAACAGTAGAGCATAGCAT    501
                      ||||||||||||||||||||||||||||||||||||||||||||||||||
92871             843 TCATTATGGGTTTTGTTAATGCTACTTCTTCGAACAGTAGAGCATAGCAT    892

OEGA2K12FOR1      502 AAATGTTTATTTATGTAAAATTTCAGTTATCAATTTTGATGGTTTATCTA    551
                      ||||||||||||||||||||||||||||||||||||||||||||||||||
92871             893 AAATGTTTATTTATGTAAAATTTCAGTTATCAATTTTGATGGTTTATCTA    942

OEGA2K12FOR1      552 CGTGACAGTAACAGTCTGTTTTGGGTGGTTCCGCCAGGATGCTGAGGAGC    601
                      ||||||||||||||||||||||||||||||||||||||||||||||||||
92871             943 CGTGACAGTAACAGTCTGTTTTGGGTGGTTCCGCCAGGATGCTGAGGAGC    992

OEGA2K12FOR1      602 ATCAAGCACCTATTTCTGGTCTTGGAGAAGTTGGTCCATCCAGTCAGTCC    651
                      ||||||||||||||||||||||||||||||||||||||||||||||||||
92871             993 ATCAAGCACCTATTTCTGGTCTTGGAGAAGTTGGTCCATCCAGTCAGTCC   1042

OEGA2K12FOR1      652 AAATTACTTGTGAACAGGGATGAAAGCCATGTGAGGAATGAAGTAAATCT    701
                      |||||||||||||.||.|||||||.|||||||||||||||||||||||||
92871            1043 AAATTACTTGTGAGCAAGGATGAAGGCCATGTGAGGAATGAAGTAAATCT   1092

OEGA2K12FOR1      702 TAAT-AAAATTCATCAAAAATATTACATGTTGATCAGATCAACCAGAAGA    750
                      |||| |||||||||||||||||||||||||||||||||||||||||||||
92871            1093 TAATAAAAATTCATCAAAAATATTACATGTTGATCAGATCAACCAGAAGA   1142
```

```
OEGA2K12FOR1      751 CAAGTGTAGATGTCACCAAGC-AGGATGTGAAGATGTT-CCTATGTGAAG    798
                      ||||||||||||||||||||| |||||||||||||||| ||||.||||||
92871            1143 CAAGTGTAGATGTCACCAAGCAAGGATGTGAAGATGTTCCCTACGTGAAG   1192

OEGA2K12FOR1      799 AG-ATGGATAGGAGACCCATTTCT-AGCTAAAAGTAGAGGAAGGATA    843
                      || |.||||||||||||||.|||| ||||||.|||||||||.|||||
92871            1193 AGAAGGGATAGGAGACCCAATTCTAAGCTAAGAGTAGAGGAGGGATA   1239


Query: OEGA2N17FOR1
Subject: 161027

OEGA2N17FOR1        1 CCTAGTGTTGACAGTGATTTATTGATATTCGCAGCTTCCTTTAGACGCTC     50
                      |||||||||||||||||||||||||||||||||||.||||||||||||||
161027             20 CCTAGTGTTGACAGTGATTTATTGATATTCGCAGCNTCCTTTAGACGCTC     69

OEGA2N17FOR1       51 ACCCTCAGCACCAGAAGTCTTTTGCCTGTTAAGGGTAATAGCAATGTGTA    100
                      ||||||||||||||||||||||||||||||||||||||||||||||||||
161027             70 ACCCTCAGCACCAGAAGTCTTTTGCCTGTTAAGGGTAATAGCAATGTGTA    119

OEGA2N17FOR1      101 AATAATGTGATAGCACGAGATATCCCATCTATTTCAGAATTTGACTAGAA    150
                      ||||||||||||||||||||||||||||||||||||||||||||||||||
161027            120 AATAATGTGATAGCACGAGATATCCCATCTATTTCAGAATTTGACTAGAA    169

OEGA2N17FOR1      151 TATGATGTAAACCTTTCGGAACCCGCAAGGTCAACAAGGTTCAGTCGAGC    200
                      ||||||||||.|||||||||||||||||||||||||||||||||||||||
161027            170 TATGATGTAANCCTTTCGGAACCCGCAAGGTCAACAAGGTTCAGTCGAGC    219

OEGA2N17FOR1      201 AAAACGGAAGTTAGTGGTTGAATCCTTTTCCCATCTACTCTCAATCACAC    250
                      ||||||||||||||||||||||||.|||||||||||||||||||||||||
161027            220 AAAACGGAAGTTAGTGGTTGAATCNTTTTCCCATCTACTCTCAATCACAC    269

OEGA2N17FOR1      251 ATGTGAAAACACTATGAGAGCGGCTACTTTCTCTGTTCATATTTGTCGCA    300
                      ||||||||||||||||||||||||||||||.|||||||||||||||||||
161027            270 ATGTGAAAACACTATGAGAGCGGCTACTTTNTCTGTTCATATTTGTCGCA    319

OEGA2N17FOR1      301 GCAACTTTCCTATTTGCTGAACCCTGAAGCAAAACTCAAATTTTAATGCA    350
                      ||||||||||||||||||||||||||||||||||||||||||||||||||
161027            320 GCAACTTTCCTATTTGCTGAACCCTGAAGCAAAACTCAAATTTTAATGCA    369

OEGA2N17FOR1      351 TAGAGACATACATTGTGCATGCACACATGCATCTTTGAAGATTGTAATCA    400
                      ||||||||||||||||||||||||||||||||||||||||||||||||||
161027            370 TAGAGACATACATTGTGCATGCACACATGCATCTTTGAAGATTGTAATCA    419

OEGA2N17FOR1      401 GAGTAAACTAAAAGACAGTGAAAATAGTCACCTGACTCAGAAGCTGAAGA    450
                      ||||||||||||||||||||||||||||||||||||||||||||||||||
161027            420 GAGTAAACTAAAAGACAGTGAAAATAGTCACCTGACTCAGAAGCTGAAGA    469

OEGA2N17FOR1      451 ATGTCGCCTACAGTTTGCACCTCAAATTCGGATAGATTTTCAACATACAC    500
                      ||||||||||||||||||||||||||||||||||||||||||||||||||
161027            470 ATGTCGCCTACAGTTTGCACCTCAAATTCGGATAGATTTTCAACATACAC    519

OEGA2N17FOR1      501 GCCTTTTTTGATGTCCTCACGTAGCTAAAAATAGAGATGATGAATATGAG    550
                      ||||||||||||||||||||||||||||||||||||||||||||||||||
161027            520 GCCTTTTTTGATGTCCTCACGTAGCTAAAAATAGAGATGATGAATATGAG    569

OEGA2N17FOR1      551 GAAACAATAATATAATCTTTTAACAATTGAAGCGCCAAAATATCCTATCA    600
                      ||||||||||||||||||||||||||||||||||||||||||||||||||
161027            570 GAAACAATAATATAATCTTTTAACAATTGAAGCGCCAAAATATCCTATCA    619

OEGA2N17FOR1      601 AAAAGGTAAAAGTACCATCAGATTTGTGGATGAAGGATCAAGAAGATCAG    650
                      |||||||||||.||||||||||||||||||||||.|||||||||||||||
161027            620 AAAAGGTAAAANTACCATCAGATTTGTGGATGAANGATCAAGAAGATCAG    669
```

```
OEGA2N17FOR1      651 ATATTTGTTCATTATAAATCTCCAGGAAAGAGCATTTGCAATTGTATATT    700
                      ||||||||||||||||||||||||||||.|||||||||||||||||||||
161027            670 ATATTTGTTCATTATAAATCTCCAGGAAGGAGCATTTGCAATTGTATATT    719

OEGA2N17FOR1      701 AGTCGTTCATCTCTTCTGATTTCCTCCTGAAAAGTAAAACACGCG-A    749
                      |||||||||||||||||||||||||||||||||||||||||||||| |
161027            720 AGTCGTTCATCTCTTCTGATTTCCTCCTGAAAAGTAAAACACGCGAA    769

OEGA2N17FOR1      750 AGAAGTGTCTTATTGCAATGAAAATGCTTATATTAGAA-GTGACTTAGCA    798
                      |||||||||||||||||||||||||||||||||||||| |||||||||||
161027            770 AGAAGTGTCTTATTGCAATGAAAATGCTTATATTAGAAGGTGACTTAGCA    819

OEGA2N17FOR1      799 AATGGAGTCAAAGAGGGTT    817
                      |||||||||||||||||||
161027            820 AATGGAGTCAAAGAGGGTT    838


Query: OEGA3A22REV1
Subject: 124756

OEGA3A22REV1        2 AAA--TCAA-----------GAGATT---CGTC----TACATATATGACT     31
                      |||  ||||           ||.|||   |.||    ||||||||||||.
124756            145 AAAGTTCAATGGCTGCTGATGAAATTTACCCTCTTTTTACATATATGACC    194

OEGA3A22REV1       32 TTGCCAAGGTCACCTTATATAGGACTCCAAATTATGGTGTCTTTTTTGGT     81
                      ||||||.||||||||||.||||||||||||||||||||||||||||||||
124756            195 TTGCCAGGGTCACCTTAAATAGGACTCCAAATTATGGTGTCTTTTTTGGT    244

OEGA3A22REV1       82 TTCCAATGCACCAAAAT-AAAAATAAGCAATCTATTAGTGTCTTTGCAAT    130
                      ||||||||||||||||| ||||||||||||||||||||||||||||||||
124756            245 TTCCAATGCACCAAAATAAAAAATAAGCAATCTATTAGTGTCTTTGCAAT    294

OEGA3A22REV1      131 GTTGATCAAATTAAAAAATAAATGAGTAATGTTATTTAACAACACTAGTA    180
                      |||||.||||||||||||||||||||||||||||||||||||||||||||
124756            295 GTTGAACAAATTAAAAAATAAATGAGTAATGTTATTTAACAACACTAGTA    344

OEGA3A22REV1      181 TTTAACATCAATTTAGATAATAGCACTAATGGTATTAGATTATAGGTTCA    230
                      |||||||||||||||.||||||.||.||||||||||||||||||||||||
124756            345 TTTAACATCAATTTAAATAATAACATTAATGGTATTAGATTATAGGTTCA    394

OEGA3A22REV1      231 ATTAAATACAATTAATTATTGTCCATTTATAAGATATATGATATGCTGGT    280
                      ||||||||||||||||||||||||||||||||||||||||||||||||||
124756            395 ATTAAATACAATTAATTATTGTCCATTTATAAGATATATGATATGCTGGT    444

OEGA3A22REV1      281 ACCTAGATTAATGTTCAATGTCAGTGAAGTGAGAACATTATTCTTATTTA    330
                      ||||||||||||||||||||||||||||||||||||||||||||||||||
124756            445 ACCTAGATTAATGTTCAATGTCAGTGAAGTGAGAACATTATTCTTATTTA    494

OEGA3A22REV1      331 CTAGTGTGTTTGCAATGTTGACCAATCACTATTGGACTATTGTCCAAAAT    380
                      ||||||||||||||||||||||||||||||||||||||||||||||||||
124756            495 CTAGTGTGTTTGCAATGTTGACCAATCACTATTGGACTATTGTCCAAAAT    544

OEGA3A22REV1      381 TGTAGACATTGTGACCTAACTCTTAAAATCAACCTACAATAGAAGAAGAC    430
                      ||||||||||||||||||||||||||||||||||||||||||||||||||
124756            545 TGTAGACATTGTGACCTAACTCTTAAAATCAACCTACAATAGAAGAAGAC    594

OEGA3A22REV1      431 ATATCTGAAGACGTCAATGGTCCCACCGTCCAAGCTAAGTTCATCTCAGC    480
                      ||||||||||||||||||||||||||||||||||||||||||||||||||
124756            595 ATATCTGAAGACGTCAATGGTCCCACCGTCCAAGCTAAGTTCATCTCAGC    644

OEGA3A22REV1      481 AAGATTCAAGACCTTTATCTGAAATTTAGCCCTTGTTCGAGTCCTATTTT    530
                      ||||||||||||||||||||||||||||||||||||||||||||||||||
124756            645 AAGATTCAAGACCTTTATCTGAAATTTAGCCCTTGTTCGAGTCCTATTTT    694
```

```
OEGA3A22REV1     531 TTATTAAAATATATTCTTCAACAGTTATTTGATCTAATTTCAATCACATA    580
                     ||||||||||||||||||||||||||||||||||||||||||||||||||
124756           695 TTATTAAAATATATTCTTCAACAGTTATTTGATCTAATTTCAATCACATA    744

OEGA3A22REV1     581 TTTTTTAATAAAATAATATTATACATAAATTTAAAATTTTTTCCGTATTT    630
                     ||||||||||||||||||||||||||||||||||||||||||||||||||
124756           745 TTTTTTAATAAAATAATATTATACATAAATTTAAAATTTTTTCCGTATTT    794

OEGA3A22REV1     631 TTTAATCACACGTTCTCCATTATAGTACCATTGCAAAATGCATGTTTTTA    680
                     |||||||||||||||||||||||||||||||||||||||||||| ||||.
124756           795 TTTAATCACACGTTCTCCATTATAGTACCATTGCAAAATGCATG-TTTTN    843

OEGA3A22REV1     681 AAAGGGGAAGGGGGGGG    697
                     .||||||||||||||||
124756           844 NAAGGGGAAGGGGGGGG    860


Query: OEGA3F20FOR1
Subject: 127667

OEGA3F20FOR1       1 ACCTAAAGTTTCCTCGTGAAATAACATTTTTGGCCTAATTAATCATTGGT     50
                     ||||||||||||||||||||||||||||||||||||||||||||||||||
127667           818 ACCTAAAGTTTCCTCGTGAAATAACATTTTTGGCCTAATTAATCATTGGT    867

OEGA3F20FOR1      51 TAAACATTACATGAAATATCACCAATAAAGTTAATATTATTCATCTCCCA    100
                     ||||||||||||||||||||||||||||||||||||||||||||||.|||
127667           868 TAAACATTACATGAAATATCACCAATAAAGTTAATATTATTCATCTNCCA    917

OEGA3F20FOR1     101 AGTGGATTTGAAAATTAAATTTAAGCTCCAGCTATTTATTAACTCATATT    150
                     ||||||||||||||||||||||.|||||||||||||||||||||||.|||
127667           918 AGTGGATTTGAAAATTAAATTTCAGCTCCAGCTATTTATTAACTCACATT    967

OEGA3F20FOR1     151 CAGATTCCTTTCTCAAACAACGAATTGAGGTACCTCTTGCATTATTAGTT    200
                     ||||||||||||||||||||||||||||||||||||||||||||||||||
127667           968 CAGATTCCTTTCTCAAACAACGAATTGAGGTACCTCTTGCATTATTAGTT   1017

OEGA3F20FOR1     201 GAGCCTGAATTGGAACATTTTGAATTTCGCTACTTTATAAAACATTTAAT    250
                     ||||||||||||||||||||||||||||||||||.|||||||||||||||
127667          1018 GAGCCTGAATTGGAACATTTTGAATTTCGCTACTNTATAAAACATTTAAT   1067

OEGA3F20FOR1     251 CCTTAACGTTGTCCCTCAGGAGTCTTCTGATTAATTCTTTCACATGCTAC    300
                     ||||||||||||||||||||||||||||||||||||||||||||||||||
127667          1068 CCTTAACGTTGTCCCTCAGGAGTCTTCTGATTAATTCTTTCACATGCTAC   1117

OEGA3F20FOR1     301 CAATTTCTCATTTATGTTTCAGTGAAGGCAGATGAATTTCATCAACGATT    350
                     ||||||||||||||||||||||||||||||||||||||||||||||||||
127667          1118 CAATTTCTCATTTATGTTTCAGTGAAGGCAGATGAATTTCATCAACGATT   1167

OEGA3F20FOR1     351 AGTTTCGAATAAGAAATAAAAAGTTCATCTATTCATGTGGATGTTGCTTG    400
                     ||||||||||||||||||||||||||||||||||||||||||||||||||
127667          1168 AGTTTCGAATAAGAAATAAAAAGTTCATCTATTCATGTGGATGTTGCTTG   1217

OEGA3F20FOR1     401 TTATGGCGTCTTTTAACCTTCTTCTCCTCTTTCCTTTCTGGTGAAAATGA    450
                     ||||||||||||||||||||||||||||||||||||||||||||||||||
127667          1218 TTATGGCGTCTTTTAACCTTCTTCTCCTCTTTCCTTTCTGGTGAAAATGA   1267

OEGA3F20FOR1     451 TAATCTTTTACAAATGTGGAACTATTTCAGGAAAATATGGATCTTCTAGA    500
                     ||||||||||||||||||||||||||||||||||||||||||||||||||
127667          1268 TAATCTTTTACAAATGTGGAACTATTTCAGGAAAATATGGATCTTCTAGA   1317

OEGA3F20FOR1     501 AGATGGATATAGAGGTGATGTTATTGGAGAAGCGACGCCTGATGTTCTTG    550
                     ||||||||||||||||||||||||||||||||||||||||||||||||||
127667          1318 AGATGGATATAGAGGTGATGTTATTGGAGAAGCGACGCCTGATGTTCTTG   1367
```

```
OEGA3F20FOR1      551 TCAGTGAATTTGTATGGTCCTACTCCTACTTAATATCTCTTGTTGTCACA    600
                      ||||||||||||||||||||||||||||||||||||||||||||||||||
127667           1368 TCAGTGAATTTGTATGGTCCTACTCCTACTTAATATCTCTTGTTGTCACA   1417

OEGA3F20FOR1      601 AATGTCCTTACCTCTCAGATTTTCCTTTGCTTTTCAGATAATATTTGCAC    650
                      ||||||||||||||||||||||||||||||||||||||||||||||||||
127667           1418 AATGTCCTTACCTCTCAGATTTTCCTTTGCTTTTCAGATAATATTTGCAC   1467

OEGA3F20FOR1      651 AAAAGTTTAATTAAATGTTCAAGTCAACATATTGATGCCCTTTGATTTCC    700
                      ||||||||||||||||||||||||||||||||||||||||||||||||||
127667           1468 AAAAGTTTAATTAAATGTTCAAGTCAACATATTGATGCCCTTTGATTTCC   1517

OEGA3F20FOR1      701 TGTTAATAAAATATTCTTCTGTGTGCTAAAATTACATTAATCAAGTATGG    750
                      ||||||||||||||||||||||||||||||||||||||||||||||||||
127667           1518 TGTTAATAAAATATTCTTCTGTGTGCTAAAATTACATTAATCAAGTATGG   1567

OEGA3F20FOR1      751 ACCTACACCAAGAAATCCAGGTCGACAGCATGA--TATTTTTTGCATCTT    798
                      |||||||||||||||||||||||||||||||||  .|||||||||||| |
127667           1568 ACCTACACCAAGAAATCCAGGTCGACAGCATGAATAATTTTTTGCATC-T   1616

OEGA3F20FOR1      799 CATTCAATCCCTTCAGTA    816
                      ||||||||||||||||||
127667           1617 CATTCAATCCCTTCAGTA   1634


Query: OEGA3K14FOR1
Subject: 104867

OEGA3K14FOR1        1 TCATGTGCCATAACATCGAGCCTATATCCCACAGAACAACATTTACAAAT     50
                      ||||||||||||||||||||||||||||||||||||||||||||||||||
104867            331 TCATGTGCCATAACATCGAGCCTATATCCCACAGAACAACATTTACAAAT    380

OEGA3K14FOR1       51 CCAAGGGCCGGATTCATATACCATTTGGTTAGAGGGAACAAGATTGATCT    100
                      ||||||||||||||||||||||||||||||||||||||||||||||||||
104867            381 CCAAGGGCCGGATTCATATACCATTTGGTTAGAGGGAACAAGATTGATCT    430

OEGA3K14FOR1      101 AGCCACGTACATCTACAATCAAATTCTTGGTACAGGCACTTGGGGTGATA    150
                      ||||||||||||||||||||||||||||||.|||||||||||||||||||
104867            431 AGCCACGTACATCTACAATCAAATTCTTGGNACAGGCACTTGGGGTGATA    480

OEGA3K14FOR1      151 GGCACTACTCCCTAATTTTTCCCAGTCTGATATGTGGAATATGTAGAGCA    200
                      ||||||||||||||||||||||||||||||||.|||||||||||||||||
104867            481 GGCACTACTCCCTAATTTTTCCCAGTCTGATANGTGGAATATGTAGAGCA    530

OEGA3K14FOR1      201 ACAGGAATACAAATCCAACCTGGGGAAGCACCGGAAAAGCCAAGTCCCTT    250
                      .|.||.||||||||||||||||||||||||||.|||||||||||.|||||
104867            531 GCNGGGATACAAATCCAACCTGGGGAAGCACCNGAAAAGCCAAGNCCCTT    580

OEGA3K14FOR1      251 TATCAAGCAATCCACACTAGAGGCCCAAGACCGAGCAAGACTGAAGCACC    300
                      ||||||||||||||||||||||||||||||||||||||||||||||||||
104867            581 TATCAAGCAATCCACACTAGAGGCCCAAGACCGAGCAAGACTGAAGCACC    630

OEGA3K14FOR1      301 AACAGAAATTAGAGAACGCACGGCGACTACGGCAATTGAACGAGGAAGGT    350
                      |||||||||||||.||||||||.||||.||||||||||||.|||||||||
104867            631 AACAGAAATTAGAAAACGCACGACGACGACGGCAATTGAATGAGGAAGGT    680

OEGA3K14FOR1      351 CAAGCAGATGCAGACCTTGACATGCCTCAAATTCCTCCACCACCACCCCA    400
                      ||||||||||||||||||||||||||||||||||||||||||||||||||
104867            681 CAAGCAGATGCAGACCTTGACATGCCTCAAATTCCTCCACCACCACCCCA    730

OEGA3K14FOR1      401 AGCTGATATGGACAATCAGGTTTTGAGGCAGATTTTCACCTCCACCACAG    450
                      ||||||||||||||||||||||||||||||||||||||||.|||||||||
104867            731 AGCTGATATGGACAATCAGGTTTTGAGGCAGATTTTCACCACCACCACAG    780
```

```
OEGA3K14FOR1      451 GGATCAGCCGCGATGTGCAGAGTCTCCAGCAGGCTATGGGAATTTTGACG    500
                      ||||||||||||||||||||||||||||||||||||||||||.|||||||
104867            781 GGATCAGCCGCGATGTGCAGAGTCTCCAGCAGGCTATGGGAACTTTGACG    830

OEGA3K14FOR1      501 ACTGAGGTACATACAACCCACCGTGACGTCCGCATTGCCTCTGATATGTC    550
                      ||||||||.||||||.|||||||||||||||||||||||.||||||||||
104867            831 ACTGAGGTGCATACAGCCCACCGTGACGTCCGCATTGCCACTGATATGTC    880

OEGA3K14FOR1      551 ACGTGCCGCCCGCCAGGATGCCCAGGCAGCTAGGAGGGAGATGGACACTA    600
                      |||||||||.||||||||||||||||||||||||||||||||||||||||
104867            881 ACGTGCCGCTCGCCAGGATGCCCAGGCAGCTAGGAGGGAGATGGACACTA    930

OEGA3K14FOR1      601 TGAATGGTCGACTAAGTGTGTACGTGGGCA-GGTGGATGGCATCCAGCTG    649
                      |||||||||||||||||||||||||||||| |||||||||||||||||||
104867            931 TGAATGGTCGACTAAGTGTGTACGTGGGCAGGGTGGATGGCATCCAGCTG    980

OEGA3K14FOR1      650 AGAGTTAATGAAACTTGGGAGCGTTTA-GCCGTTTGAAGACACGTGTTGA    698
                      ||.||.||||||||||||||||||||| ||||||||.|||||||.|||||
104867            981 AGGGTAAATGAAACTTGGGAGCGTTTAGGCCGTTTGGAGACACGGGTTGA   1030

OEGA3K14FOR1      699 GGGGATCCACGAGGAAATGACACAGCAGTCAGCACTGATGAGAGAAATCA    748
                      ||||||||||||.|||||||||||||||||||||||||||||||||||||
104867           1031 GGGGATCCACGATGAAATGACACAGCAGTCAGCACTGATGAGAGAAATCA   1080

OEGA3K14FOR1      749 TATCTCGCCTTC    760
                      ||||||||||||
104867           1081 TATCTCGCCTTC   1092


Query: OEGA3N17REV1
Subject: 105766

OEGA3N17REV1        1 AGAGGCGCTGCTGCACTTCTGCAAGAGGCGTTGCACGGTGGCGGTCGAAG     50
                      ||||||||||||||||||||.||||||||||||||||.|||||.||.|||
105766            182 AGAGGCGCTGCTGCACTTCTACAAGAGGCGTTGCACGATGGCGCTCTAAG    231

OEGA3N17REV1       51 TGGATCTCTCAGCGTCGACGCCGCTGCGAACAGTTTCTCTGTGTAGGTGG    100
                      ||||||||||||||||.|.|||||||.|||||||||||||||..||||||
105766            232 TGGATCTCTCAGCGTCAATGCCGCTGTGAACAGTTTCTCTGTACAGGTGG    281

OEGA3N17REV1      101 ACGTGGGTGGTTTTGGTGAC--------------GGTGGTGCGGCGGATCT    137
                      ||||||||||||||||||||              ||||||||||.||||||
105766            282 ACGTGGGTGGTTTTGGTGACGGTGTGGAGTGATGGTGGTGCGGTGGATCT    331

OEGA3N17REV1      138 AGGTAGGACGACAGTGGTGGTAATGGTAGCGACGGTCTCCTGTTAGCAGT    187
                      ||||||||.|||.||||||||.|||||||||.||||||||||||||||||
105766            332 AGGTAGGATGACGGTGGTGGTGATGGTAGCGGCGGTCTCCTGTTAGCAGT    381

OEGA3N17REV1      188 GGGGATTTGGGTGGTGTTGGTGAGGTTGGGAACGGTGTATTGTTAGGTGT    237
                      .|||||||||||||||||||||||||||||||||||||||||||||||||
105766            382 AGGGATTTGGGTGGTGTTGGTGAGGTTGGGAACGGTGTATTGTTAGGTGT    431

OEGA3N17REV1      238 GTTTGGTGGTGGTTCTTGTTATGGTTCTCGCCCTGGTGGCTGTGGTGGTT    287
                      ||||||||||||||||||||.|||||.|||.|||||||||||||||||||
105766            432 GTTTGGTGGTGGTTCTTGTTGTGGTTGTCGTCCTGGTGGCTGTGGTGGTT    481

OEGA3N17REV1      288 TTTGGCGATGGTCGTGTTGGCGGTGGAGAAGGTAGTGAGTTTGTGGGTTG    337
                      ||||||||||||||||||||||||||||||||||||||||||||||||||
105766            482 TTTGGCGATGGTCGTGTTGGCGGTGGAGAAGGTAGTGAGTTTGTGGGTTG    531

OEGA3N17REV1      338 GGTATTTTTGGTGTGTTTGGTGGTGGCGGTTGTGGTGGTTTTTGGCAGCG    387
                      ||||||||||||||||||||||||||||||||||||||||||||.|||||
105766            532 GGTATTTTTGGTGTGTTTGGTGGTGGCGGTTGTGGTGGTTTTTGCCAGCG    581
```

```
OEGA3N17REV1      388 ACGAAGGTGATTGTTCTTGGCAACCATTTTGTTGGCTGTGATGTCGTGGA    437
                      |||||||||||||||||||||..|||||||||||||||||||||||||||
105766            582 ACGAAGGTGATTGTTCTTGGCNNCCATTTTGTTGGCTGTGATGTCGTGGA    631

OEGA3N17REV1      438 GTTGGTGTTAGTGGTGGTTGT    458
                      |||||||||.||||.||||||
105766            632 GTTGGTGTTGGTGGCGGTTGT    652
```

Query: OEGA5M07FOR1
Subject: 1206

```
OEGA5M07FOR1        1 AACGTGTTTTACTGAAATTCATTAAATTCATTCAAAAAATAATTATCTCC     50
                      ||||||||||.|||||||||||||||||||||..||||||||||||||||
1206              246 AACGTGTTTTTCTGAAATTCATTAAATTCATTAGAAAAATAATTATCTCC    295

OEGA5M07FOR1       51 CAAAGCAATTCATAATTGAGTTTTCAGGCGAGATTTTTAATAATTTAGT    100
                      ||||||||||||||||||||||||||||||||||||||||||||||||||
1206              296 CAAAGCAATTCATAATTGAGTTTTCAGGCGAGATTTTTAATAATTTAGT    345

OEGA5M07FOR1      101 TCTTAATTGTGTATTTACAAGAATAATATACTGTTTTTCCATTACATTTT    150
                      |||||||||||||||||.||.||||||||||||||||||.||||.|||||
1206              346 TCTTAATTGTGTATTTAGAAAAATAATATACTGTTTTTCTATTAGATTTT    395

OEGA5M07FOR1      151 TTCCATGTAGTTTTTCCTATTTAGCTTTTTTATGTTTTGAAGGTTACGAA    200
                      ||||||||||.||||||||||||||||||||||.||.|||||||||||||
1206              396 TTCCATGTAGCTTTTCCTATTTAGCTTTTTTATATTCTGAAGGTTACGAA    445

OEGA5M07FOR1      201 TTCCATAACAATGTGAGTTATAATTTTGTGATTCA-TTTTTTCCTCCTAT    249
                      |.|||||||||.||||||||||||||||.|||||| |||||||||.||||
1206              446 TACCATAACAANGTGAGTTATAATTTTGNGATTCATTTTTTTCCTNCTAT    495

OEGA5M07FOR1      250 TAGTGTAGCGCATCCATAGGTGAACCCACATGGGTTCATGTTGGCGGGGT    299
                      ||||||||.|.|||||||||.||||||||||||||||||||.||.|||||
1206              496 TAGTGTAGNGNATCCATAGGNGAACCCACATGGGTTCATGTNGGNGGGGT    545

OEGA5M07FOR1      300 CAAGTGACCCCACATATTTTTTTTTGAAAAATCCAT-ATTGTTTGATGTC    348
                      |||||||||||||||..||||||||| ||||||.|| |||||||||||||
1206              546 CAAGTGACCCCACATTNTTTTTTTTG-AAAATCTATAATTGTTTGATGTC    594

OEGA5M07FOR1      349 AAAAAAAATTGAAA-ATTGTTATTTTTAATCCCATTTCAA---GCCTCAT    394
                      |||.|||||||||| ||| .||||.|||||||||||||||   | |||||
1206              595 AAATAAAATTGAAATATT-NTATTCTTAATCCCATTTCAAAGTG-CTCAT    642

OEGA5M07FOR1      395 AACCTA-TTTTTGCTATTTAATGGCCCTTAAAAT-GTTAACTATTGAAAA    442
                      .||||| ||||||||.|||||||||.|.|.||.| |||||||||||.|||
1206              643 GACCTATTTTTTGCTTTTTAATGGCACATGAAGTGGTTAACTATTGTAAA    692
```

Query: OEGA6E01FOR1
Subject: 100173

```
OEGA6E01FOR1        1 GTACCGTCATTTGGCATTATTTAATTTCTCACTGAAATAAGTAATAGGGT     50
                      |||||.|.|.|||||||.|||||.|||||||.|||||||||.||||||||
100173            629 GTACCTTAACTTGGCATCATTTAGTTTCTCATTGAAATAAGCAATAGGGT    678

OEGA6E01FOR1       51 GGCGCTCTTAGCTCAAAACTCCACCTATCCCTACCGCAGAGGCATCGCAT    100
                      |||..||.|||||| ||||||||||||..||||||.|.||||||||.|||
100173            679 GGCTTTCATAGCTC-AAACTCCACCTACTCCTACCTCGGAGGCATCACAT    727

OEGA6E01FOR1      101 TCTACCTC-AAAAACTTTGAAAAAATCGGGAAGTCGCATGACTGGAGCTT    149
                      ||.||||| ||||||||||.|.|||||.|||||||||||||.||||||||
100173            728 TCGACCTCAAAAAAACTTTGGAGAAATCAGGAAGTCGCATGATTGGAGCTT    777
```

```
OEGA6E01FOR1     150 CTGTCATTCACCTCTTAACCAACTTAAATGCTTTGGTCGCTTCATGCGAC    199
                     |.||||||..|||.|||||||.|||.||||||||||...|||..||.|.|
100173           778 CCGTCATTTGCCTTTTAACCATCTTTAATGCTTTGGCTACTTTGTGTGTC    827

OEGA6E01FOR1     200 CAATAAAATTTCCCTTTTTTCATGCACTCCGTAATAGGAGCCATGATCGT    249
                     |||||||||||.||  |.||||||.||||.|||||||||||.||||||||
100173           828 CAATAAAATTTTCC--TCTTCATGAACTCTGTAATAGGAGCTATGATCGT    875

OEGA6E01FOR1     250 GCTAAACCCTTTAATGAAACGACGATAAAAGGTAGCGAAACCATG-AAAC    298
                     ||                       |||||||||||.|||||||| |||.
100173           876 GC-----------------------TAAAAGGTAGCTAAACCATGAAAAT    902

OEGA6E01FOR1     299 TCCGAACGTTATGTATAGTCTTGGTCTCAAGCCAACCAACAATGGCCTGA    348
                     |||.|||||.||.||||||||||||.||||.|||||||||.||||||||||
100173           903 TCCTAACGTCATATATAGTCTTGGGCTCAGGCCAACCAATAATGGCCTGA    952

OEGA6E01FOR1     349 ATTTTTGTAGGGTCAGCAGACACTCCTGTCGCGGAGACAATAAAACCTAG    398
                     ||.||||||||||.|||.||.|.||||||||||||.|||||.||.|||||
100173           953 ATCTTTGTAGGGTTAGCTGATATTCCTGTCGCGGAAACAATGAAGCCTAG   1002

OEGA6E01FOR1     399 AAATACAACTTTTATCTGTGAAGAAAAGAACATTTCTTCACATTAGCATA    448
                     .||.||.|| .|||||||||||| |||||||||||||.||||||||||||
100173          1003 GAAGACGAC-ATTATCTGTGAAG-AAAGAACATTTCTNCACATTAGCATA   1050

OEGA6E01FOR1     449 CAAACTTGTAGCGCCGAAAGGTAGAACAAACCCTGCATGAGATGATCTCA    498
                     |||.|||||.|| .||||.||||||||||| ||||..|||.||||||||.
100173          1051 CAAGCTTGTTGC-ACGAAGGGTAGAACAAA-CCTGTGTGAAATGATCTCG   1098

OEGA6E01FOR1     499 ACGTCGTTCCCTTAGTTTTTACAGTACACGAGAAATGTCATCAAGGGAG    547
                     |.||.|||||| ||| ||||||.|||.||.|| |||||||||||.|.||
100173          1099 ATGTTGTTCCC-TAG-TTTTACTGTAAACAAG-AATGTCATCAAAGTAG   1144


Query: OEGA7A16FOR1
Subject: 158805

OEGA7A16FOR1       1 ATCAAGCAAGAAATCTTCTTTGCCATTGACACCAGGCCACAGGCTTGCAA     50
                     ||||||||||||||||||||||||||||||||||||||||||||||||||
158805           377 ATCAAGCAAGAAATCTTCTTTGCCATTGACACCAGGCCACAGGCTTGCAA    426

OEGA7A16FOR1      51 TGTCTGAATGTGGCTGTGATTCAAGAAGAGAATTCAATGG-TTTTGAGGC     99
                     |||||||||||||||||||||||||||||||||||||||| |||||||||
158805           427 TGTCTGAATGTGGCTGTGATTCAAGAAGAGAATTCAATGGTTTTTGAGGC    476

OEGA7A16FOR1     100 ATTTAGGAATCTTGAAAATTCTGATGGCAATGATAGAAAGGTGATCGGAA    149
                     ||||||||||||||||||||||||||||||||||||||||||||||||||
158805           477 ATTTAGGAATCTTGAAAATTCTGATGGCAATGATAGAAAGGTGATCGGAA    526

OEGA7A16FOR1     150 AAAGTAAGAGGGATAGTTTTTCAGTGTCTAAAATTTGGCTAAGGGGAAAG    199
                     ||||||||||||||||||||||||||||||||||||||||||||||||||
158805           527 AAAGTAAGAGGGATAGTTTTTCAGTGTCTAAAATTTGGCTAAGGGGAAAG    576

OEGA7A16FOR1     200 AAGTATAAACCCAATTCAGCCACTGCTGAGGCATCCAGACGAGCATTTTC    249
                     ||||||||||||||||||||||||||||||||||||||||||||||||||
158805           577 AAGTATAAACCCAATTCAGCCACTGCTGAGGCATCCAGACGAGCATTTTC    626

OEGA7A16FOR1     250 TTTCCGATTTCCAGTTCACTGGAATGCTGTGGATGAGGCCAAGGCTGGTG    299
                     ||||||||||||||||||||||||||||||||||||||||||||||||||
158805           627 TTTCCGATTTCCAGTTCACTGGAATGCTGTGGATGAGGCCAAGGCTGGTG    676

OEGA7A16FOR1     300 GCGGAAGTGGTGGTGCTAGGAGGGCTGTATCTGAAATTGATATTAGTAGG    349
                     ||||||||||||||||||||||||||||||||||||||||||||||||||
158805           677 GCGGAAGTGGTGGTGCTAGGAGGGCTGTATCTGAAATTGATATTAGTAGG    726
```

```
OEGA7A16FOR1      350 TGGGAGAATGGAGGGAGTGAATTTGGGCTGTGATGAGGAAATCCGAAGCT    399
                      ||||||||||||||||||||| ||||||||||||||||||||||||||||
158805            727 TGGGAGAATGGAGGGAGTGAA-TTGGGCTGTGATGAGGAAATCCGAAGCT    775

OEGA7A16FOR1      400 GTAACAGTCTTGATTCTCAAGCAAATCAGCCCATCTTTTGCAAAGAGGAC    449
                      ||||||||||.|||||||||||||||||| ||||||||||||||||||||
158805            776 GTAACAGTCTNGATTCTCAAGCAAATCAG-CCATCTTTTGCAAAGAGGAC    824

OEGA7A16FOR1      450 TCTACTTTGGCTTATGGGGACACAAAAT-AGTTGGTTCATTCATCTTTTT    498
                      |||||||||||||||||||||||||||| ||||||||||.||||||||||
158805            825 TCTACTTTGGCTTATGGGGACACAAAATAAGTTGGTTCAATCATCTTTTT    874

OEGA7A16FOR1      499 CATACAATGTTTAGTTTTATCTTATCAATGGGACTTCA-AAATAGGGGAA    547
                      |||||||||||||||||||||||||||||||||||||| |||||||||||
158805            875 CATACAATGTTTAGTTTTATCTTATCAATGGGACTTCAGAAATAGGGGAA    924

OEGA7A16FOR1      548 ACTAGAAACTAGAAAAG-ATAGATTATC-GTACCAAATAATAT    588
                      ||||||||||||||||| ||||| |||| ||||.|||||||||
158805            925 ACTAGAAACTAGAAAAGAATAGA-TATCAGTACAAAATAATAT    966


Query: OEGA7C17REV1
Subject: 104811

OEGA7C17REV1        1 GTAGTTAAAAAGAGTGTAGGTTTTCATTGGGGTGATGAACAGGAACTTGC     50
                      |||||||||||||||||||||||||||||||||||||||||.||||||||
104811            199 GTAGTTAAAAAGAGTGTAGGTTTTCATTGGGGTGATGAACANGAACTTGC    248

OEGA7C17REV1       51 ATTTAATACTGTTAAAGGTAGATTGTGTGAAGCACCTGTTTTGGCATTAC    100
                      ||||||||||.|||||||||||||||||||||||||||||||||||||||
104811            249 ATTTAATACTATTAAAGGTAGATTGTGTGAAGCACCTGTTTTGGCATTAC    298

OEGA7C17REV1      101 CTAACTTTGATAAAACAGTTGAGATTGAGTGTGATGCATCAGGAATAGGG    150
                      ||||||||||.||||||.|.||||||||||||||||||||||||||||||
104811            299 CTAACTTTGANAAAACATTCGAGATTGAGTGTGATGCATCAGGAATAGGG    348

OEGA7C17REV1      151 ATTGGAGCAGTTTTAATGTAGGATCAGAGGCCTATTGCATACTTTAGCGA    200
                      ||||||||||||||||||.||||||.||||||||||||||||||||||||
104811            349 ATTGGAGCAGTTTTAATGCAGGATCGGAGGCCTATTGCATACTTTAGCGA    398

OEGA7C17REV1      201 GAAATTGACTGGAGCTGCTTTAAATTATCCTACATATGACAAAGAACTTT    250
                      ||||||||.|||||||||||||||||||||||||||||||||||||||||
104811            399 GAAATTGAGTGGAGCTGCTTTAAATTATCCTACATATGACAAAGAACTTT    448

OEGA7C17REV1      251 ATGCACTTGTTCGAGCTTTGGAGACTTGGCAACATTATCTATGGCCGAAG    300
                      ||||||||||||||||||||||||||||||||||||||||||||||||||
104811            449 ATGCACTTGTTCGAGCTTTGGAGACTTGGCAACATTATCTATGGCCGAAG    498

OEGA7C17REV1      301 GAGTTTGTAATACATACCGATCATGAGTCACTAAAGCACTTGTGGGGACA    350
                      |||||.||||||||.|||||||||||||||||||||||||||.|||||||
104811            499 GAGTTCGTAATACACACCGATCATGAGTCACTAAAGCACTTGCGGGGACA    548

OEGA7C17REV1      351 AGGTAAATTGAACAAGAGGCATGCACAGTGGATGAAATTCATTGAGACCT    400
                      ||||||||||||||||||||||||||.|||||||.|||||||||||||||
104811            549 AGGTAAATTGAACAAGAGGCATGCACGGTGGATGGAATTCATTGAGACCT    598

OEGA7C17REV1      401 TTCCATACGTCATTCGGTATAAACAAGTAAAGGAAAATGTGGTGGCGGAT    450
                      ||||||||||||||||||||||.|.||..||||||||||||.|.||||||
104811            599 TTCCATACGTCATTCGGTATAANCNAGGTAAGGAAAATGTGNTNGCGGAT    648

OEGA7C17REV1      451 GCGTTGTCGTGAAAGTACGTATTGCTCACTACTCTTGATTCTAAGTTACT    500
                      |||||||||.|||.||||..||||||.|||||||||.|||||||||||||
104811            649 GCGTTGTCGCGAAGGTACNNATTGCTAACTACTCTTNATTCTAAGTTACT    698
```

```
OEGA7C17REV1      501 TGGATTTGAACATATT-AGGAATTGTATG-AAAAGATGTTGAATTTTTGA    548
                      |||.|||||||||||| |||||||||||| |||||||||||||||||.|.
104811            699 TGGNTTTGAACATATTAAGGAATTGTATGAAAAAGATGTTGAATTTTNGG    748

OEGA7C17REV1      549 ATATTTATTTGTCATGCAT-AAAGGGGCGGTTGATA-GTTCTTTA-GCAT    595
                      ||||||||||||||||||| |||||||.|||||||| |||||||| ||||
104811            749 ATATTTATTTGTCATGCATAAAAGGGGTGGTTGATAGGTTCTTTAGGCAT    798

OEGA7C17REV1      596 GAGGGTTATTTATTT--GGAAAATAGGTTATGGTGTTCCTAATTGT    639
                      |||||.|||||||||  |||||||||||||| ||||||.|||||||
104811            799 GAGGGGTATTTATTTCGGGAAAATAGGTTAT-GTGTTCNTAATTGT    843


Query: OEGA8A14REV1
Subject: 37000

OEGA8A14REV1        1 ATCTAATTTTTCTGTATTTTGTGG-AAAGATTTGGCCTTGTAATGGAAGA     49
                      |||||||||||||||||||||||| |||||||||||||||||||||||||
37000             848 ATCTAATTTTTCTGTATTTTGTGGAAAAGATTTGGCCTTGTAATGGAAGA    897

OEGA8A14REV1       50 GATATTGTGGAATCTTTGAAGCTATATGGGTTGTCTTTATTTGCTATTTT     99
                      ||||||||||||||||||||||||||||||||||||||||||||||||||
37000             898 GATATTGTGGAATCTTTGAAGCTATATGGGTTGTCTTTATTTGCTATTTT    947

OEGA8A14REV1      100 AGCTGATGTTTAGAACTGGGTTTGAGCTATTTTGTTATACTTTGACGTTT    149
                      ||||||||||||||||||||||||||||||||||||||||||||||||||
37000             948 AGCTGATGTTTAGAACTGGGTTTGAGCTATTTTGTTATACTTTGACGTTT    997

OEGA8A14REV1      150 TTTGACTAGAACTTAGCTCTTGGAGTGGAAGAGATATTTTGTTTTCTTTT    199
                      ||||||||||||||||||||||||||||||||||||||||||||||||||
37000             998 TTTGACTAGAACTTAGCTCTTGGAGTGGAAGAGATATTTTGTTTTCTTTT   1047

OEGA8A14REV1      200 GCTCTATTTGGGTTTTCTACAGAATGGCTGAAAAGTACTTTTCTGTTTTT    249
                      ||||||||||||||||||||||||||||||||||||||||||||||||||
37000            1048 GCTCTATTTGGGTTTTCTACAGAATGGCTGAAAAGTACTTTTCTGTTTTT   1097

OEGA8A14REV1      250 TTTTCCTACATTTGACTAATTTCTGAAATGATTTGAGTAATTTTAGGTGA    299
                      ||||||||||||||||||||||||||||||||||||||||||||||||||
37000            1098 TTTTCCTACATTTGACTAATTTCTGAAATGATTTGAGTAATTTTAGGTGA   1147

OEGA8A14REV1      300 TTCTTGGGGCTGGAGGAAATAATACTGGAAGTGAAGGGTTATTTGGTAAA    349
                      ||||||||||||||||||||||||||||||||||||||||||||||||||
37000            1148 TTCTTGGGGCTGGAGGAAATAATACTGGAAGTGAAGGGTTATTTGGTAAA   1197

OEGA8A14REV1      350 GGTTTTTCCAGGTGGAGGGAGGTGGGCTAGGATTTTGGGTTTTGTAAAAC    399
                      ||||||||||||||||||||||||||||||||||||||||||||||||||
37000            1198 GGTTTTTCCAGGTGGAGGGAGGTGGGCTAGGATTTTGGGTTTTGTAAAAC   1247

OEGA8A14REV1      400 ATGGGATTAGGTGGTGATGCATTGAAGAAGGAGTCTTGGGATGTGGGGAA    449
                      ||||||||||||||||||||||||||||||||||||||||||||||||||
37000            1248 ATGGGATTAGGTGGTGATGCATTGAAGAAGGAGTCTTGGGATGTGGGGAA   1297

OEGA8A14REV1      450 ATCAAAGGGGAGAAAGAAGAAAGATGGTGACTCTGAGGAGGAGACCGGGT    499
                      ||||||||||||||||||||||||||||||||||||||||||||||||||
37000            1298 ATCAAAGGGGAGAAAGAAGAAAGATGGTGACTCTGAGGAGGAGACCGGGT   1347

OEGA8A14REV1      500 GTTGGATTAAATTGAGGTTTATTGGCAGCTGTATGTCATCAAGATCTAAA    549
                      ||||||||||||||||||||||||||||||||||||||||||||||||||
37000            1348 GTTGGATTAAATTGAGGTTTATTGGCAGCTGTATGTCATCAAGATCTAAA   1397

OEGA8A14REV1      550 GTTGATAGCTCCATTAGTAGCATCAGCACTCACTGTGGTAATG-CTTTAA    598
                      ||||||||||||||||||||||||||||||||||||||||||| ||||||
37000            1398 GTTGATAGCTCCATTAGTAGCATCAGCACTCACTGTGGTAATGTCTTTAA   1447
```

```
OEGA8A14REV1     599 CTTGTAGC-TTCAA-TTTTTATTTATTTATTTTTTTATATCTCGACTGGT    646
                     |||||||| ||||| |||||||||||||| ||||||||||||||||||||
37000           1448 CTTGTAGCTTTCAATTTTTTATTTATTTA-TTTTTTATATCTCGACTGGT   1496

OEGA8A14REV1     647 ATGAAGTTTTGCTTAATAGTA    667
                     ||||   .||||||||||||||
37000           1497 ATGA--GTTTGCTTAATAGTA   1515


Query: OEGA8J23REV1
Subject: 110847

OEGA8J23REV1       1 ATTCGAATTGAACCATATATCCTTTTG-AACCTAAACAAATAAGCAAAAT     49
                     ||||||||||||||||||||||||||| ||||||||||||||||||||||
110847          1249 ATTCGAATTGAACCATATATCCTTTTGAAACCTAAACAAATAAGCAAAAT   1298

OEGA8J23REV1      50 GAGCCTGTGGACTACAAACCATTCAAAAAACTAGAAGTAAACTGGCATTC     99
                     ||||||||||||||||||||||||||||||||||||||||||||||||||
110847          1299 GAGCCTGTGGACTACAAACCATTCAAAAAACTAGAAGTAAACTGGCATTC   1348

OEGA8J23REV1     100 GCTAGATAGTTATAAGTTCATCCTAGTTTAGATCGAATAGACATTAATGT    149
                     ||||||||||||||||||||||||||||||||||||||||||||||||||
110847          1349 GCTAGATAGTTATAAGTTCATCCTAGTTTAGATCGAATAGACATTAATGT   1398

OEGA8J23REV1     150 AAAGTAGGATAATTAGAAACTTTATTAAAGAGAATACAAATTGAAAGATT    199
                     ||||||||||||||||||||||||||||||||||||||||||||.|||||
110847          1399 AAAGTAGGATAATTAGAAACTTTATTAAAGAGAATACAAATTGANAGATT   1448

OEGA8J23REV1     200 AAAGGTACAATAGAAGAAGCTTCAACCCATTCCATGACAAATTCAGTTGA    249
                     ||||||||||||||||||||||||||||||||||||||||||||||||||
110847          1449 AAAGGTACAATAGAAGAAGCTTCAACCCATTCCATGACAAATTCAGTTGA   1498

OEGA8J23REV1     250 AAAATAGTTTTATCATTGATTCAGTTTTAACGAATTAAAAATCCTTGCCG    299
                     ||||||||||||||||||||||||||||||||||||||||||||||||||
110847          1499 AAAATAGTTTTATCATTGATTCAGTTTTAACGAATTAAAAATCCTTGCCG   1548

OEGA8J23REV1     300 GAACGGTTGATGCATATATTGGGTAATTAGCATTTTAACTGAATATAAAG    349
                     ||||||||||||||||||||||||||||||||||||||||||||||||||
110847          1549 GAACGGTTGATGCATATATTGGGTAATTAGCATTTTAACTGAATATAAAG   1598

OEGA8J23REV1     350 ATTTTCGTTCTTCCCATCATCATTTTTAACATAGATCTCCATTTGATCAC    399
                     ||||||||||||||||||||||||||||||||||||||||||||||||||
110847          1599 ATTTTCGTTCTTCCCATCATCATTTTTAACATAGATCTCCATTTGATCAC   1648

OEGA8J23REV1     400 TCCTGATACTTCCTCCACCATTGTTAATTTTATTATATTTAATGAATATC    449
                     ||||||||||||||||||||||||||||||||||||||||||||||||||
110847          1649 TCCTGATACTTCCTCCACCATTGTTAATTTTATTATATTTAATGAATATC   1698

OEGA8J23REV1     450 CTAATATTAAAGTCTACTTTGAGAAGTACAAAAATCTAATTTTCTGATTC    499
                     ||||||||||||||||||||||||||||||||||||||||||||||||||
110847          1699 CTAATATTAAAGTCTACTTTGAGAAGTACAAAAATCTAATTTTCTGATTC   1748

OEGA8J23REV1     500 TACAATAATCTTTTGAAGTGAACAGATTAATAAGGATAAAATTACAACAT    549
                     ||.||||||||||||||||||||||||||||||||.||||||||||||||
110847          1749 TATAATAATCTTTTGAAGTGAACAGATTAATAAGGANAAAATTACAACAT   1798

OEGA8J23REV1     550 CATCAAGAATATTAAATTTATCTAGCAAGTAGAAATCTTTGGAATGAAAG    599
                     ||||||||||||||||||||||||||||||||||||||||||||||||||
110847          1799 CATCAAGAATATTAAATTTATCTAGCAAGTAGAAATCTTTGGAATGAAAG   1848

OEGA8J23REV1     600 AGTTGAATTACAGTACTTCATCAAGAATAAACGAGTGCT-CTCGTAAAAA    648
                     ||||||||||||||||.||||||||||||||||||||.| ||||||||||
110847          1849 AGTTGAATTACAGTACTACATCAAGAATAAACGAGTGGTACTCGTAAAAA   1898
```

```
OEGA8J23REV1     649 AAAAAAATTGAGAAAGGTTTATGGTCTTAATGGAATATAAAGGGATAGTA   698
                     |||.|||||||||||||||||||||||||||||||||| |||||||||||
110847          1899 AAATAAATTGAGAAAGGTTTATGGTCTTAATGGAATAT-AAGGGATAGTA  1947

OEGA8J23REV1     699 GCAATGATGCTAAC-AGTCTTTCACATAGATGTCCTTGAATTCCGCGATT   747
                     |||||||||||||| |||||||||||||||||||||||||||||||||||
110847          1948 GCAATGATGCTAACAAGTCTTTCACATAGATGTCCTTGAATTCCGCGATT  1997

OEGA8J23REV1     748 GTGTGATAAA-GTTTTCGAGCCAAAATTTTTAAAACAATTTCCTACGTCT   796
                     |||||||||| ||||||||||.||||||||||.||| ||||||||.||||
110847          1998 GTGTGATAAAGGTTTTCGAGCTAAAATTTTTACAAC-ATTTCCTATGTCT  2046

OEGA8J23REV1     797 GTTGTT    802
                     ||||||
110847          2047 GTTGTT   2052


Query: OEGA8M11FOR1
Subject: 132300

OEGA8M11FOR1       1 ATTTTATGAGATATGGAAGAATTAAAGAGATTGTTTAATATTAAAGAATC    50
                     ||||||||||||||||||||||||||||||||||||||||||||||||||
132300           191 ATTTTATGAGATATGGAAGAATTAAAGAGATTGTTTAATATTAAAGAATC   240

OEGA8M11FOR1      51 AATACTTGCCTGAAATCCATATAACATAATCAATTAAGTGATAATATACT   100
                     ||||||||||||||||||.|||||||||||||||||||||||||||||||
132300           241 AATACTTGCCTGAAATCCGTATAACATAATCAATTAAGTGATAATATACT   290

OEGA8M11FOR1     101 TCAATATAAGTAAACAAGAAAACACATGGAATTAGAATTAGAAGTAGCAT   150
                     |||||||||||||||||||||.|||||||||||||||||||||..|||||
132300           291 TCAATATAAGTAAACAAGAAAGCACATGGAATTAGAATTAGAAANAGCAT   340

OEGA8M11FOR1     151 GAATTAATTATCAACGAAAATCTAGTTATGAATTTTTAGCATATAGGGAA   200
                     ||||.|||||.|||||||.|||||||.||.||||||||||||||||||||
132300           341 GAATAAATTAACAACGAAGATCTAGTGATAAATTTTTAGCATATAGGGAA   390

OEGA8M11FOR1     201 AGAGAATTGTTTCATATTAGCCAAACAGAAAAGATACAATTGAAATTTAA   250
                     ||||||||||||||||||||||||||||||||||||||||||||||||||
132300           391 AGAGAATTGTTTCATATTAGCCAAACAGAAAAGATACAATTGAAATTTAA   440

OEGA8M11FOR1     251 TAGATCATAGATAAGTCAAGAGTCCCATCAGTGTAGCCTTTTTTGTAAAC   300
                     ||||||||||||||||||||||||||||||||||||||||||||||||||
132300           441 TAGATCATAGATAAGTCAAGAGTCCCATCAGTGTAGCCTTTTTTGTAAAC   490

OEGA8M11FOR1     301 CGTAAACATTGAGAGTTCATTATTTAGTAGTTCAAACACACGTGTCATCC   350
                     .||||||||||||||||||||||||||||||||||||||||||||||.||
132300           491 NGTAAACATTGAGAGTTCATTATTTAGTAGTTCAAACACACGTGTCACCC   540

OEGA8M11FOR1     351 ATTTGTAGTTGTTTCTCTCTAACATAATCTGTCAATCTATGGTGAGACAT   400
                     ||||||||||||||||||||||.||.|||||||||.||||||||||||||
132300           541 ATTTGTAGTTGTTTCTCTCTAAAATCATCTGTCAACCTATGGTGAGACAT   590

OEGA8M11FOR1     401 CGTATTACACCATTTGGTCTTAAAGTTTGTAGTAACATTCCAATTGCGAT   450
                     |||||||||||||||||||||||||||||||||.||||||||||||||||
132300           591 CGTATTACACCATTTGGTCTTAAAGTTTGTAGTNACATTCCAATTGCGAT   640

OEGA8M11FOR1     451 ATGAAGAGTCGTGAATTTGAACATTGCTTACAAATTTCAGCAAATGTTGT   500
                     ||||||||||||||||||||||||||||||||||||||||||||||||||
132300           641 ATGAAGAGTCGTGAATTTGAACATTGCTTACAAATTTCAGCAAATGTTGT   690

OEGA8M11FOR1     501 CTAGCATATCTTCCAAGTATATACTGCAAAGTAAAAAGATAAATCAATGA   550
                     ||||||||||||||||||||||||||||||||||||.|||||||||||||
132300           691 CTAGCATATCTTCCAAGTATATACTGCAAAGTAAAANGATAAATCAATGA   740
```

```
OEGA8M11FOR1     551 ATTATCGAATTTGAAATTATGTGGAAAAATGTGAATATGGAATGACATTT    600
                     ||||||||||||.||||||||||.||||..||||||||||||||||||||
132300           741 ATTATCGAATTTNAAATTATGTGNAAAANNGTGAATATGGAATGACATTT    790

OEGA8M11FOR1     601 TATACATTTAAGTAAAGAAAGATACAAGTGATAAACAATCGAAGTTAAAG    650
                     ||||||||||||||||||||||||||||||||||||||||||||||||||
132300           791 TATACATTTAAGTAAAGAAAGATACAAGTGATAAACAATCGAAGTTAAAG    840

OEGA8M11FOR1     651 AGAACCTTACAATTTTTCGATGACCGTTGTGATTAGCTGTTAATGTTACT    700
                     |||||||||||||||||||||||.|||||||||||||||.||||||||||
132300           841 AGAACCTTACAATTTTTCGATGANCGTTGTGATTAGCTGNTAATGTTACT    890

OEGA8M11FOR1     701 TCAAAGACAAGATTACCTGTAAGTAAAGTGTCTTGTTCA-GAATATCATG    749
                     ||||||||||||||||||.|||||||||||||||||||| ||||||||..
132300           891 TCAAAGACAAGATTACCTNTAAGTAAAGTGTCTTGTTCAGGAATATCANN    940

OEGA8M11FOR1     750 GTCGTGTATTACCTCTAGCCCCGAACGGTATAGTATTGTGACGTTGAATG    799
                     ||.||.||||||||||.|||||||||||||||||||||||||||||||||
132300           941 GTNGTNTATTACCTCTNGCCCCGAACGGTATAGTATTGTGACGTTGAATG    990

OEGA8M11FOR1     800 TAGAATTCTCGTTAATATTCAAACACAAG-AGATAACCATCTTGT-AGGT    847
                     ||||||||||||| ||||||||||||||| ||||||||||||||| ||.|
132300           991 TAGAATTCTCGTT-ATATTCAAACACAAGAAGATAACCATCTTGTAAGTT   1039

OEGA8M11FOR1     848 ATGATGGTAAAAAAA    862
                     |||||||||||||||
132300          1040 ATGATGGTAAAAAAA   1054


Query: OEGA9O02FOR1
Subject: 142205

OEGA9O02FOR1       1 TTATCTATTGTAAGGAGATAAGAAAAATTATGGGAAGGATGAAAAAAGGC     50
                     ||||||||||||||||||||||||||||||||||||||||||||||||||
142205           877 TTATCTATTGTAAGGAGATAAGAAAAATTATGGGAAGGATGAAAAAAGGC    926

OEGA9O02FOR1      51 AACGTGTCTCCAAACGACAGTGCAGAAGTCAGCTTTCCTAAAAGGCTATA    100
                     ||||||||||||||||||||||||||||||||||||||||||||||||||
142205           927 AACGTGTCTCCAAACGACAGTGCAGAAGTCAGCTTTCCTAAAAGGCTATA    976

OEGA9O02FOR1     101 AGGTAGTCATCATCGATTAGCATTAGAAGAGCAAAACATCTCCAAAAGTC    150
                     ||||||||||||||||||||||||||||||||||||||||||||||||||
142205           977 AGGTAGTCATCATCGATTAGCATTAGAAGAGCAAAACATCTCCAAAAGTC   1026

OEGA9O02FOR1     151 CATTCCTTGTGTAGGACCCATGCACCAATCACAGTTCAATTTATTTTGTC    200
                     ||||||||||||||||||||||||||||||||||||||||||||||||||
142205          1027 CATTCCTTGTGTAGGACCCATGCACCAATCACAGTTCAATTTATTTTGTC   1076

OEGA9O02FOR1     201 ATAATACCACAATGACATTAGCGATTCCAATTTATAAATAAGATAGCATT    250
                     ||||||||||||||||||||||||||||||||||||||||||||||||||
142205          1077 ATAATACCACAATGACATTAGCGATTCCAATTTATAAATAAGATAGCATT   1126

OEGA9O02FOR1     251 TTCGTAAATTCCATAATTAAATTTTTACTCATTGATGACACTCAGAAGTC    300
                     ||||||||||||||||||||||||||||||||||||||||||||||||||
142205          1127 TTCGTAAATTCCATAATTAAATTTTTACTCATTGATGACACTCAGAAGTC   1176

OEGA9O02FOR1     301 TTAATCAGTCAATCAAAATACGTATGTACTAAATACACTAAAGTACAAAT    350
                     ||||||||||||||||||||||||||||||||||||||||||||||||||
142205          1177 TTAATCAGTCAATCAAAATACGTATGTACTAAATACACTAAAGTACAAAT   1226

OEGA9O02FOR1     351 TATCCATAACATAATATATGAAAAAAGTCAAACTTAGTTTACTCGGAAGG    400
                     ||||||||||||||||||||||||||||||||||||||||||||||||||
142205          1227 TATCCATAACATAATATATGAAAAAAGTCAAACTTAGTTTACTCGGAAGG   1276
```

```
OEGA9O02FOR1     401 AAGAACCGTAAGAGTAAAGGACACTCCTCGTGATCAACCTCAACTTTCTC    450
                     ||||||||||||||||||||||||||||||||||||||||||||||||||
142205          1277 AAGAACCGTAAGAGTAAAGGACACTCCTCGTGATCAACCTCAACTTTCTC   1326

OEGA9O02FOR1     451 TTTATTTCCTCTTGCAATGCTCCAAAAGTGTGGAACTAGGACACTCATCA    500
                     ||||||||||||||||||||||||||||||||||||||||||||||||||
142205          1327 TTTATTTCCTCTTGCAATGCTCCAAAAGTGTGGAACTAGGACACTCATCA   1376

OEGA9O02FOR1     501 AAGACTAAACAATGATAGAGGTCGTGATGTACACACATCGTACTTAACAA    550
                     ||||||||||||||||||||||||||||||||||||||||||||||||||
142205          1377 AAGACTAAACAATGATAGAGGTCGTGATGTACACACATCGTACTTAACAA   1426

OEGA9O02FOR1     551 GTGATTTTTAAAATTTACATCTTTGTTAGTGTTTTGCTTTTCCACGTCTT    600
                     ||||||||||||||||||||||||||||||||||||||||||||||||||
142205          1427 GTGATTTTTAAAATTTACATCTTTGTTAGTGTTTTGCTTTTCCACGTCTT   1476

OEGA9O02FOR1     601 TTATAATTAACATATGAATACTGTGGAAAAATTTCATAACTACAATATAA    650
                     ||||||||||||||||||||||||||||||||||||||||||||||||||
142205          1477 TTATAATTAACATATGAATACTGTGGAAAAATTTCATAACTACAATATAA   1526

OEGA9O02FOR1     651 TTTTAAAATTCATAGATTTGTCAAAAAACTTTCAAAAACTAAATAGCATG    700
                     ||||||||||||||||||||||||||||||||||||||||||||||||||
142205          1527 TTTTAAAATTCATAGATTTGTCAAAAAACTTTCAAAAACTAAATAGCATG   1576

OEGA9O02FOR1     701 TAATGGTGGCCTTTTCCAATAAATGATCGTAACCCAT-AATTTTATGATT    749
                     ||||||||||||||||||||||||||||||||||||| ||||||||||||
142205          1577 TAATGGTGGCCTTTTCCAATAAATGATCGTAACCCATAAATTTTATGATT   1626

OEGA9O02FOR1     750 GTGACATATTATATGATGTCTAATTGTGAAATAATTTATATGTGACATAT    799
                     ||||||||||||||||||||||||||||||||||||||||||||||||||
142205          1627 GTGACATATTATATGATGTCTAATTGTGAAATAATTTATATGTGACATAT   1676

OEGA9O02FOR1     800 TATTTGTTAGTACGCGACT    818
                     |||||||||||||||||||
142205          1677 TATTTGTTAGTACGCGACT   1695
```
