# Supplementary material for: The Peculiar Landscape of Repetitive Sequences in the Olive (Olea europaea L.) Genome
Source: Genome Biol Evol. 2014 Mar 26;6(4):776–91. doi: 10.1093/gbe/evu058 (PMC4007544; doi:10.1093/gbe/evu058)
Supplement: Supplementary Data [file supp_evu058_Barghini_et_al_SupplMat_S5.pdf]

Supplementary materials S5.

Structural domains in 26 putative LTR-retrotransposons re-constructed from 26 clusters produced by RepeatExplorer starting from 454 reads. For the procedure used for reconstruction see Materials and Methods. The structure was determined by dot plot analysis (to survey the occurrence of direct repeats, corresponding to putative LTRs), by BLAST against NCBI non-redundant database and analyzing sequences at Pfam (http://pfam.sanger.ac.uk/search) website (to identify retrotransposon domains). LTR: Long Terminal Repeat; GAG: capsid protein; INT: integrase; RT: reverse transcriptase; RH: RNAse H; AP: aspartic protease; CHR: chromodomain.

Cluster	Superfamily	5’-LTR	GAG	INT	RT	RH	3’-LTR	Sequence Length	LTR Length*
24	Copia	YES	YES	YES	YES	YES	YES	6,812	889
39	Copia	YES	YES	YES	YES	YES	YES	5,069	806
48	Copia	NO	NO	YES	YES	YES	YES	4,650	1,545
63	Copia	NO	YES	YES	YES	YES	YES	8,624	1,267
66	Copia	YES	YES	YES	YES	YES	YES	5,326	799
72	Copia	YES	YES	YES	YES	YES	YES	7,008	1,589
86	Copia	YES	YES	YES	YES	YES	YES	6,049	1,375
90	Copia	YES	YES	YES	NO	NO	NO	3,506	1,667
102	Copia	NO	YES	YES	YES	YES	YES	5,465	1,626
108	Copia	NO	YES	YES	YES	YES	YES	6,441	1,633
112	Copia	YES	YES	YES	YES	YES	YES	6,689	1,746
114	Copia	NO	YES	YES	YES	YES	YES	6,587	1,818
142	Copia	YES	YES	YES	YES	YES	YES	5,197	789
165	Copia	NO	YES	YES	YES	YES	YES	5,268	798
172	Copia	NO	YES	YES	YES	YES	YES	5,222	1,575
178	Copia	YES	YES	YES	YES	YES	YES	4,712	322
212	Copia	YES	YES	YES	YES	YES	YES	4,688	294
213	Copia	YES	YES	YES	YES	YES	YES	4,906	419
239	Copia	YES	YES	YES	YES	YES	YES	5,028	541
262	Copia	NO	YES	YES	YES	YES	YES	6,442	1,616

		5’-LTR	GAG	AP	RT	RH	INT	CHR	3’-LTR	Sequence Length	LTR Length*
45	Gypsy	YES	YES	YES	YES	YES	YES	YES	YES	5,879	635
69	Gypsy	NO	YES	YES	YES	YES	YES	NO	YES	5,813	1,031
146	Gypsy	YES	YES	NO	YES	YES	YES	NO	YES	5,608	782
149	Gypsy	YES	YES	NO	YES	YES	YES	NO	YES	6,483	581
157	Gypsy	NO	YES	YES	YES	YES	YES	NO	YES	5,253	1,072
180	Gypsy	NO	YES	YES	YES	YES	YES	YES	YES	6,098	622

*The longest putative LTR is reported.
